# Scalable Engineering of Bio‐Manufactured Extracellular Vesicles for Selective Delivery in Ovarian Cancer Patient‐Derived Models

**DOI:** 10.1002/advs.75415

**Published:** 2026-04-23

**Authors:** Nihar Godbole, Andrew Lai, Alexander Quinn, Marianne Gillard, Dominic Guanzon, Katherin Scholz‐Romero, Alexis Salas‐Burgos, Bastián Lillo Dapremont, Rohan Lourie, Naven Chetty, Richard Lobb, Kate Beecher, Amy E. McCart Reed, Kaltin Ferguson, Biao Sun, Yaowu He, John D. Hooper, Carlos Salomon

**Affiliations:** ^1^ Translational Extracellular Vesicles in Obstetrics and Gynae‐Oncology Group Frazer Institute Faculty of Health Medicine and Behavioural Sciences Royal Brisbane and Women's Hospital The University of Queensland Herston Queensland Australia; ^2^ UQ Centre for Extracellular vesicle Nanomedicine Faculty of Health Medicine and Behavioural Sciences The University of Queensland Herston Queensland Australia; ^3^ Commonwealth Scientific and Industrial Research Organisation, Agriculture & Food Saint Lucia Queensland Australia; ^4^ Frazer Institute Faculty of Health, Medicine and Behavioural Sciences The University of Queensland Herston Queensland Australia; ^5^ Australian Institute for Bioengineering and Nanotechnology (AIBN) The University of Queensland Brisbane Queensland 4072 Australia; ^6^ Oncology Progression Laboratory Pharmacology Department Biological Science Faculty Concepcion University Concepción Chile; ^7^ Centro Territorial de Investigación Oncológica Integrativa (CTIOi) Santiago Chile; ^8^ Mater Health Services South Brisbane Queensland Australia; ^9^ Mater Research Institute Translational Research Institute University of Queensland Woolloongabba Queensland Australia

## Abstract

Ovarian cancer remains challenging to treat because of late diagnosis, heterogeneity, and poor response to existing therapies. Here, we present a streamlined and scalable extracellular vesicle (EV) engineering approach that enables efficient cargo packaging and enhances targeted cargo delivery to ovarian tumors in vivo. Systematic comparison of vesicle‐anchoring domains identifies an optimized scaffold for loading bioluminescent cargo into EVs. We characterized EV production from five cell lines commonly used for biopharmaceutical manufacturing and selected and stably engineered ExpiCHO cells as a robust, large‐scale source of engineered EVs (eEVs). To achieve molecular targeting, the transmembrane scaffold Δ688 PTGFRN is modified to display tissue‐targeting ligand Ephrin‐B2 (EB2) on the EV surface, exploiting its high‐affinity interaction with the Eph receptor B4 (EphB4), which is overexpressed in advanced ovarian carcinoma. Following systemic administration, these eEVs carrying bioluminescent cargo preferentially accumulate in EphB4‐positive cells in vivo and permit non‐invasive, spatiotemporal tracking via cargo‐mediated bioluminescence resonance energy transfer. In patient‐derived xenograft models with differential EphB4 expression, Ephrin‐B2–displaying eEVs show selective localization to EphB4‐positive tumors and report intratumoral cargo distribution. This work establishes a translational strategy for large‐scale eEV production, advancing EV‐based delivery platforms for precision targeting of EphB4‐expressing ovarian cancer.

## Graphical abstract

Engineered extracellular vesicles displaying Ephrin‐B2 selectively target Ephrin‐B4–expressing ovarian cancer cells, enabling precise delivery in patient‐derived models. This scalable bio‐manufacturing platform reveals a versatile strategy to exploit Ephrin signaling for highly specific therapeutic payload delivery and motivates exploration of tailored EV‐based interventions in heterogeneous ovarian tumours.

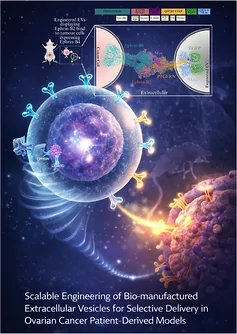

## 1 Introduction

Ovarian cancer remains the sixth leading cause of cancer‐related mortality among women worldwide, primarily due to its late detection, recurrence, and treatment resistance [1, 2]. Conventional therapies have improved survival outcomes in patients with early‐stage disease; however, their effectiveness remains limited once tumors progress to advanced metastatic stages [3]. Although immunotherapies represent a major advance in the treatment of advanced disease, their clinical impact has been constrained by lower response rates across patient populations [4]. Moreover, despite advances in systemic therapies, the effective delivery of therapeutic agents to advanced tumors remains a major challenge, frequently hindered by off‐target toxicity, rapid systemic clearance, and inadequate accumulation at the disease site [5, 6]. Accordingly, receptor‐mediated delivery systems that leverage disease‐specific molecular signatures may provide a promising strategy for improving therapeutic precision and advancing personalized treatment approaches [7, 8, 9].

One receptor that has attracted increasing attention in ovarian cancer is Eph receptor‐B4 (EphB4), a receptor tyrosine kinase reported to have increased expression in various cancer types, including ovarian cancer [10, 11, 12]. Its overexpression in ovarian tumors is associated with more advanced disease stage, where it plays an important role in tumor growth and metastatic dissemination by promoting angiogenesis and is often linked to poor prognosis [12]. EphB4 is also instrumental in multiple embryonic development processes, including cell aggregation, migration, vascular remodeling, and neural development [12, 13, 14]. Unlike other Eph receptors, EphB4 is the sole member of the receptor family that preferentially interacts with only one ligand, Ephrin‐B2 (EB2) [15, 16]. Compared with other commonly explored ovarian cancer‐associated receptors such as FRα, HER2, or EpCAM, EphB4 represents a biologically active receptor involved in tumor vascular signaling and cellular communication within the tumor microenvironment [17, 18, 19]. Therefore, EphB4 represents an opportunity to enhance the specificity of therapeutics to ovarian tumors by developing ligand‐directed delivery systems that address both safety and efficacy.

Advances in molecular medicine have stimulated considerable interest in the development of nanoscale drug delivery platforms. Liposomes, polymeric nanoparticles, and inorganic nanocarriers such as gold and silica have been explored for ovarian cancer therapy [20, 21, 22, 23, 24, 25]. These systems can be functionalized with ligands to promote receptor‐mediated delivery to tumor cells. Among these, liposomal nanocarriers have been extensively investigated due to their ability to encapsulate chemotherapeutic agents and improve drug stability [20, 21]. Gold nanoparticles have been investigated due to their tunable surface chemistry, which enables efficient conjugation of therapeutic agents and targeting ligands [22]. Similarly, mesoporous silica nanoparticles have been investigated due to their high surface area and large pore volume, allowing efficient loading and controlled release of therapeutic cargo [23]. However, many of these platforms remain limited by poor tumor penetration, rapid systemic clearance, and off‐target accumulation [26]. These limitations have motivated interest in biologically derived delivery systems such as extracellular vesicles that can preferentially deliver therapeutic cargo to anatomically challenging sites in vivo [26, 27, 28, 29]. EVs are membranous nanoparticles that are naturally secreted by the cells into extracellular spaces [30]. They encapsulate bioactive molecules from the parent cells and facilitate intercellular communication by delivering that cargo to recipient cells that internalize the EVs [30, 31]. The ability of EVs to shield their contents from degradation [32], combined with high biocompatibility [33], low immunogenicity [34], and remarkable delivery potential [31, 35], makes EVs a promising platform for delivering diverse molecular cargoes to target cells.

Engineering EVs to efficiently incorporate and deliver functional cargo can enhance their therapeutic potential [26, 29, 36, 37]. However, efficient packaging of EVs is a challenge. Multiple studies have demonstrated a clinically viable approach of endogenously packaging cargo proteins through genetically modifying EV‐producer cells, achieving uniform loading without compromising EV structure or morphology [26, 28, 29, 36, 38, 39, 40, 41]. Cells can be transiently or stably engineered to express cargo proteins fused either to transmembrane proteins [28, 29, 36, 38] naturally incorporated into EVs or to membrane‐homing peptides [26, 40, 41] that bind to the intraluminal membrane surface. These vesicle‐anchoring domains (VADs) are recognized by cellular machinery and direct cargo sorting into the EV lumen via endosomal or non‐endosomal pathways [28, 29]. Endogenous engineering can also be used to direct cargo proteins to the EV surface or lumen, depending on whether they function as targeting ligands or therapeutic payloads [26, 29, 37, 42]. Native EV‐transmembrane proteins such as CD63, LAMP2B, and PTGFRN exhibit dual functionality, as their extracellular domains allow fused cargo to be displayed on the EV surface and their intracellular domains enable fusion of cargo protein for intraluminal packaging [28, 38, 42]. For instance, the dual functionality of PTGFRN was initially demonstrated by Dooley et al. [28]. Engineering its intraluminal C‐terminal domain enabled significantly enhanced cargo packaging into EVs, while modification of its extraluminal N‐terminal domain led to enriched expression of functional proteins on the EV surface [28]. Similarly, CD63 has also been shown to exhibit dual functionality by supporting the expression of cargo protein on both the EV surface and within its lumen [38, 39]. In another instance, LAMP2B was shown to enrich a short glycoprotein peptide on the EV surface, facilitating passage across the blood‐brain barrier in vivo. Accordingly, Alharbi et al. [42] demonstrated its dual functionality in mediating EphB4 cargo display on EV surface and facilitating cargo enrichment within the lumen. Alternative strategies for intraluminal packaging involve the addition of lipidation tags to cargo molecules via co‐ and post‐translational modifications such as myristoylation, palmitoylation, and prenylation to the N‐terminus of the target proteins; the lipid tags bind the cargo proteins to the internal EV lipid membrane, thereby improving the efficiency of cargo loading [26, 40, 41, 43]. For instance, protein N‐myristoylation driven by a consensus sequence Met‐Gly‐X‐X‐X‐Ser/Thr involves covalent attachment of a myristoyl group (lipidation) to the second glycine residue, thereby facilitating its membrane association. Peptides derived from SRC family kinases (8‐amino acids) and charged multivesicular body 6 (CHMP6) (11‐amino acids) have been shown to enhance the loading of exogenous cargo proteins inside the EVs [40, 41].

Scalable production of regulatory‐compliant EVs is a major bottleneck for their therapeutic translation. Mesenchymal stem cells have typically been used to harvest EVs for therapeutic applications, but their limited expansion potential and senescence are an impediment to scalability [44, 45]. In contrast, bio‐pharmaceutically compatible cell lines such as Chinese Hamster Ovary (CHO) cells and Human Embryonic Kidney 293 (HEK293) cells offer scalable and consistent EV production [46, 47, 48]. Furthermore, these cells can be readily engineered, making them ideal platforms for bio‐manufacturing engineered EVs (eEVs) [42, 46, 49]. Over the years, efforts to isolate EVs from HEK293 cells have largely focused on improving vesicle yield, enhancing downstream recovery, and enabling therapeutic engineering under scalable, GMP‐compatible conditions [50, 51, 52, 53]. However, the potential of other industrially relevant production hosts has received comparatively less attention. CHO cells represent the predominant mammalian production platform for large‐scale manufacturing of recombinant therapeutic proteins, with industrial processes routinely achieving markedly high yields [54, 55]. These cells exhibit robust growth in suspension cultures using chemically defined serum‐free media, a feature that also facilitates efficient harvesting of EVs from conditioned media. Notably, CHO cells exhibit well‐characterized post‐translational modifications that are broadly compatible with clinical applications [55]. Recent studies have shown that CHO‐derived EVs contain defined protein, RNA, and lipid cargo and that their secretion and composition can vary with culture conditions and recombinant protein expression [56, 57, 58]. In addition, EVs released from CHO production cell lines have been reported to influence culture performance and have been explored as potential carriers for therapeutic proteins [59, 60, 61]. Although these cells are well‐suited for large‐scale production, early‐stage studies often rely on transient expression systems, which limit their suitability for translation development due to batch variability. As a result, a significant limitation hindering the downstream application of eEVs in vivo is the challenge of scalable production consistently ensuring high yield and purity [47, 62]. Stable cell lines, though more challenging to develop, offer a consistent and translationally viable solution for generating eEVs with uniform cargo loading [63].

Another major limitation of EV‐based therapeutic delivery is their short half‐life and limited targeting ability [31]. After systemic administration, EVs often accumulate in clearance organs such as the liver, kidney, and spleen, limiting their bioavailability at disease sites [31, 49]. One way to improve the accumulation of EVs at the tissue of interest is to apply the EVs harvested from the cells derived from the same tissue [64, 65]. However, using EVs derived from diseased cells for targeted therapeutics can pose a risk of inducing unintended consequences. For example, while tumor‐derived EVs exhibit organotropism, they have also been shown to orchestrate a metastatic cascade in the recipient organs [65, 66]. An alternative approach to enhance tissue targeting, which avoids the biological risks associated with vesicles derived from diseased or pathologically altered cells, is to functionalize the EV surface by engineering them to express tissue‐specific ligands [35, 36, 37]. For instance, EVs displaying a neuron‐specific rabies viral glycoprotein peptide were shown to traverse the blood‐brain barrier and deliver siRNA cargo to neuronal and glial cells [36]. Accordingly, this study demonstrated that surface modification can facilitate EV accumulation in otherwise inaccessible tissues while minimizing systemic clearance [36].

Despite recent progress in EV engineering, studies focusing on the in vivo spatiotemporal biodistribution of eEVs are limited [26, 49]. Traditional labelling methods, such as lipophilic dyes or radiolabeling of membrane proteins, have notable limitations [42, 67, 68, 69, 70] while reporter systems incorporating fluorescent (e.g., EGFP, mCherry) or bioluminescent proteins (e.g., Nanoluciferase, firefly luciferase) have emerged as more promising alternatives [26, 71, 72]. In particular, fusing fluorescent and bioluminescent reporters using a short linker peptide enables bioluminescence resonance energy transfer (BRET), improving sensitivity and accuracy for in vivo tracking [26, 72]. In BRET, light energy emitted by substrate oxidation of Nanoluciferase (∼460 nm) is non‐radiatively transferred to the acceptor fluorophore (e.g., EGFP), stimulating fluorescence emission at a longer wavelength (∼520 nm). [26, 72]. To enable precise in vivo tracking of eEVs in our study, we used a model cargo consisting of EGFP fused to the N‐terminus of Nanoluciferase.

**FIGURE 1:**
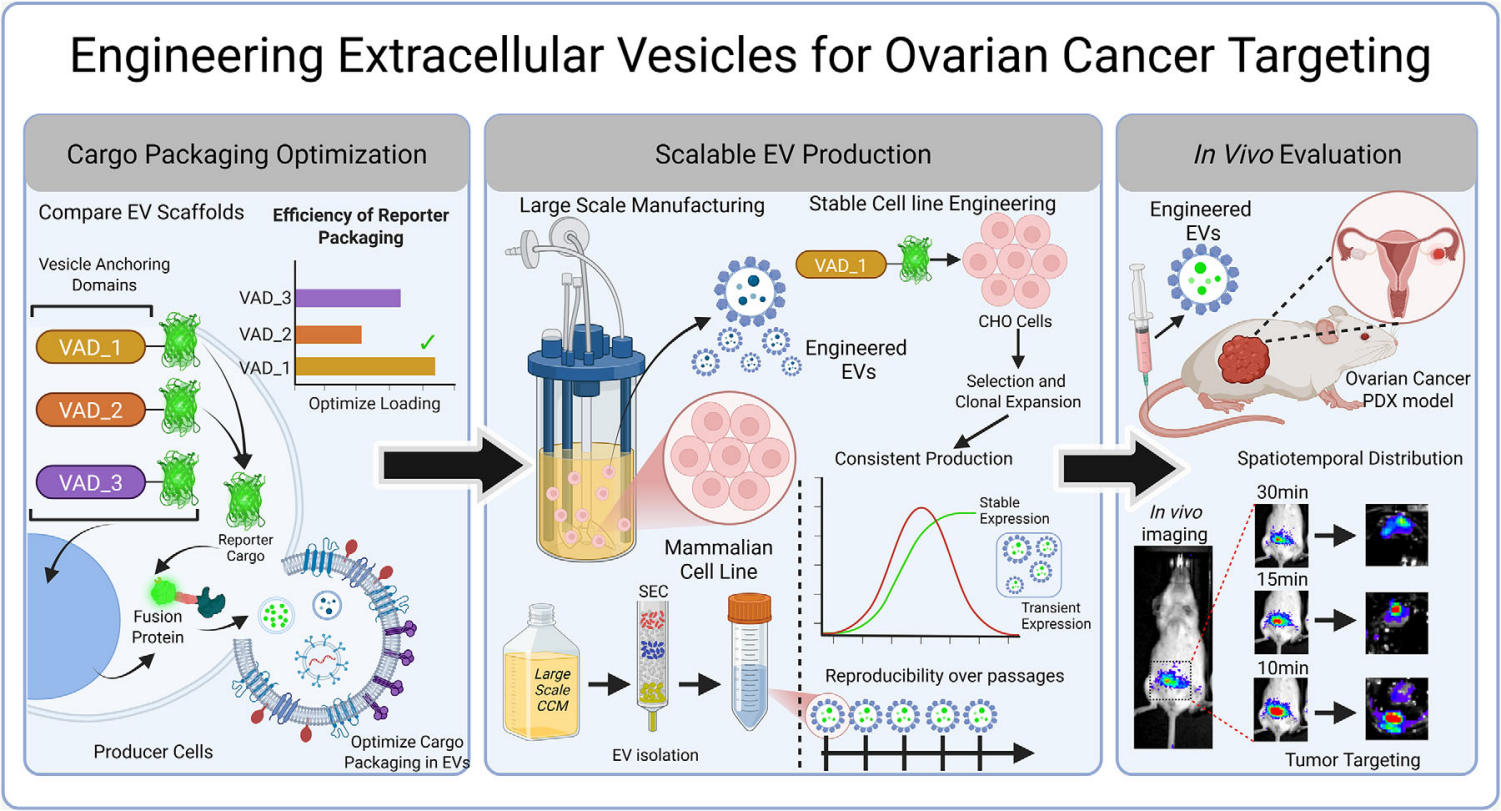
Engineering extracellular vesicles for targeted delivery in ovarian cancer. EV cargo loading was optimized by comparing vesicle‐anchoring domains to enhance reporter protein packaging. The selected strategy was applied to scalable production using mammalian cell systems, followed by the generation of stable engineered cell lines for consistent EV yield. Engineered EVs were then evaluated in ovarian cancer PDX models to assess in vivo biodistribution and tumor targeting.

**FIGURE 2:**
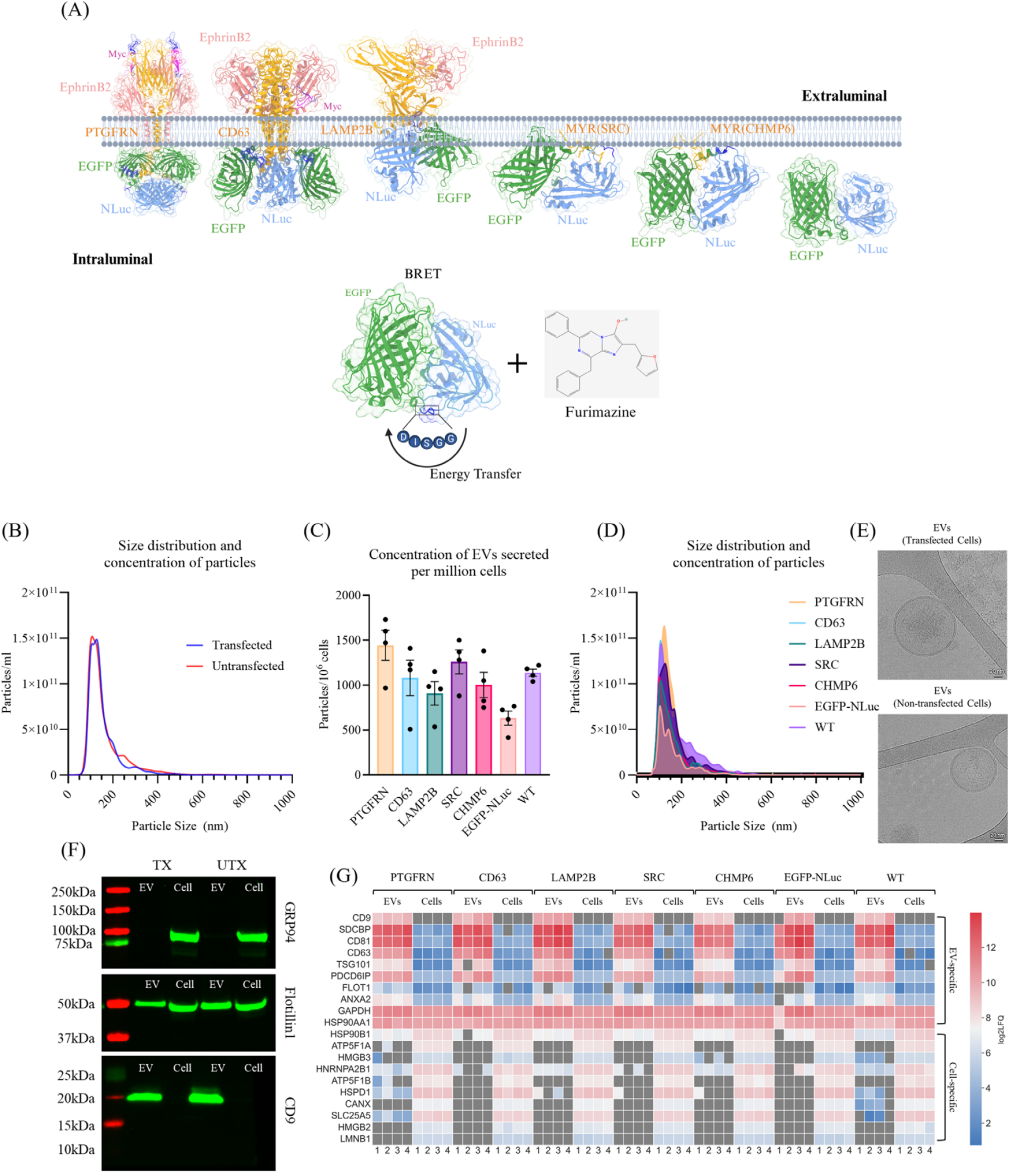
Analyses to test transfection‐induced artefacts in EV preparation. (A) Schematics of different EV engineering strategies using vesicle‐associated domains (VADs) of PTGFRN, CD63, LAMP2B, and protein‐N‐myristoylation motifs (peptides) derived from SRC kinase family and charged multivesicular body protein 6 (CHMP6) and passive packaging control (PPC) EGFP‐NLuc without VAD. (B) Size distribution of EV isolated from transfected and untransfected cells, determined by NTA. (C) Relative yield of EVs (particles/million cells) from different cell transfection treatments, determined by NTA. (D) Size distribution of EVs isolated from different cell transfection treatments, determined by NTA. (E) Cryo‐TEM images demonstrating intact lipid bilayer structures and spherical morphology across all EV groups. (F) Immunoblots of EV protein extracts, identifying enrichment of canonical markers CD9 and Flotillin‐1. GRP94 (endoplasmic reticulum marker) was used as a negative control. (G) Proteomic characterization of EVs and corresponding cell lysates to assess the presence and relative abundance of EV‐associated and non‐EV proteins. Data are presented as mean ± SEM unless otherwise indicated. Statistical significance was assessed using one‐way ANOVA with multiple comparisons performed using Tukey's tests, with *p* < 0.05 considered significant. ^*^ (*p* ≤ 0.05), ^**^ (*p* ≤ 0.01), ^***^ (*p* ≤ 0.001), ^****^ (*p* ≤ 0.0001).

In the present study, we demonstrate the in vivo selective tumor accumulation properties of eEVs that display the Ephrin‐B2 surface ligand and are also packaged with reporter protein cargo (Figure 1). To address the need for efficient and scalable EV‐based delivery, we compared multiple vesicle‐anchoring domains (VADs) for their ability to package a dual‐reporter protein, EGFP‐Nanoluciferase (EGFP‐NLuc), into EVs. This reporter cargo was fused to the C‐terminus of each of three transmembrane proteins (truncated PTGFRN [28], CD63 [38, 39], and LAMP2B [36, 42]), as well as to each of two lipid‐modified peptides that bind to inner EV membranes (SRC [41] and CHMP6 [73] myristoylated peptides), selected based on their enrichment in EV membranes (Figure 2A). A control dual‐reporter protein lacking a VAD was used to benchmark the degree to which cargo proteins were passively loaded into EVs, in the absence of any active mechanism associating the cargo proteins with the EV membrane (Figure 2A). To facilitate translational production, we evaluated five bio‐pharmaceutically relevant cell lines(ExpiCHO, Lonza‐CHO, CHO‐S, FLP‐IN‐CHO, and Expi293) [74, 75] for their ability to produce eEVs carrying exogenous cargo. Based on EV yield, cargo packaging, and proteomic consistency, we developed a stable ExpiCHO‐based producer line enabling scalable and uniform eEV production. Finally, we used this stable ExpiCHO line to produce Ephrin‐B2 displaying eEVs and assessed their targeting specificity and biodistribution after systemic administration in Eph receptor‐B4 positive patient‐derived xenograft murine models of high‐grade serous ovarian cancer.

## 2 Results

### 2.1 Comparison of Cargo Protein Packaging by Vesicle Anchoring Domains

The engineered EVs (eEVs) were produced using a cell engineering approach with adherent HEK293T cells transiently transfected with DNA plasmids (Plasmid #1–#5, Table 1) encoding one of the five VAD‐EGFP‐NLuc fusion proteins. EGFP fluorescence from the live cells was analyzed to determine the transfection efficiency of each plasmid (percentage of EGFP‐positive cells; Figure S1A). The EGFP‐NLuc passive packaging control (PPC) and SRC plasmids were the most efficiently transfected and resulted in significantly more EGFP‐positive cells than other VAD plasmids (Figure S1B).

**TABLE 1:** Summary of engineered plasmids detailing fusion protein composition.

Plasmid number	Fusion protein	Plasmid size (kb)	Targeting ligand (EphrinB2)	Vesicle anchoring domain	Cargo
1	EphrinB2‐PTGFRN‐EGFP‐NLuc	∼6.7	+	PTGFRN	EGFP‐NLuc
2	EphrinB2‐CD63‐EGFP‐NLuc	∼6.8	+	CD63	
3	EphrinB2‐LAMP2B‐EGFP‐NLuc	∼7.3	+	LAMP2B	
4	MYR(SRC)‐EGFP‐NLuc	∼5.3	—	MYR (SRC)	
5	MYR(CHMP6)‐EGFP‐NLuc	∼5.3	—	MYR (CHMP6)	
6	EGFP‐NLuc	∼5.3	—	N/A	
7	EphrinB2‐PTGFRN‐EGFP	∼6.0	+	PTGFRN	EGFP
8	PTGFRN‐EGFP‐NLuc	∼6.1	—	PTGFRN	EGFP‐NLuc

The cell‐conditioned media were harvested and processed for EV isolation, and adherent cells were lysed to extract total protein. Immunoblotting confirmed EGFP expression, as a part of the fusion protein, in transfected cells (Figure S1C). EGFP expression was strongest in PPC (∼47 kDa), SRC (∼47 kDa), and CHMP6 (∼47 kDa), moderate in PTGFRN (∼96 kDa) (two distinct EGFP‐reactive bands), and weaker in CD63 (100 kDa) and LAMP2B (117 kDa) expressing cells. Nanoluciferase activity measured in cell lysates (Figure S1D) mirrored the pattern of EGFP‐positive cells (Figure S1B). Data independent acquisition (DIA) mass spectrometry (MS) with label‐free quantification (LFQ; DIA‐LFQ) also showed a similar pattern; PPC and SRC plasmid‐transfected cells exhibited the highest EGFP and NLuc levels, and CD63 cells, the lowest (Figure S1E,F).

Global proteomic profiling performed using DIA‐MS on cell lysates revealed transfection‐induced proteomic shifts. Differentially expressed proteins were identified using log2 fold‐change thresholds (>1.0 for upregulation, <−1.0 for downregulation, *p* < 0.05) (Figure S2A). Across all transfected cells, five proteins (CSTB, FKBP1A, PFN, RABL6, SNRPC) were consistently upregulated, while three (ALB, LTF, SLC2A1) were downregulated relative to untransfected (referred to as wild‐type (WT)) cells (Figure S1D). Each VAD exhibited a unique proteomic signature, PTGFRN (30 up, 16 down), CD63 (14 up, 17 down), LAMP2B (17 up, 15 down), SRC (32 up, 14 down), CHMP6 (42 up, 12 down), and PPC (20 up, 14 down) (Figure S2A). Comparison of proteins unique to or shared with WT cells revealed VAD‐dependent remodeling of the cellular proteome (Figure S2B). For instance, PTGFRN‐transfected cells exhibited the largest unique protein set (557), whereas CHMP6 and SRC displayed fewer unique proteins (329 and 361, respectively), indicating that the extent of proteomic divergence varied across DNA constructs (Figure S2B). Functional enrichment analysis of the significantly dysregulated cellular proteins revealed that fusion protein expression induced selective VAD‐dependent remodeling of host‐cell molecular machinery (Figures S3 and S4). PTGFRN (Figures S3 and S4A), SRC (Figures S3 and S4D), and CHMP6(Figures S3 and S4E) were characterized by enrichment of increased proteins in nucleosome assembly, chromatin organization, and ribonucleoprotein biogenesis, together with nuclear and nucleolar compartments, consistent with increased protein (EGFP‐NLuc) expression. In parallel, downregulated proteins in these groups mapped strongly to transport, cytoskeleton, and secretory granule lumen‐associated functions, indicating their roles in cellular trafficking machinery. In CD63 (Figure S3B) expressing cells, downregulated proteins were associated with processes such as chromosome condensation, DNA recombination, and chromatin organization, indicating convergence of these dysregulated proteins on genome regulatory pathways. Similarly, in LAMP2B (Figure S3C) and PPC (Figure S3C) expressing cells decreased proteins linked to nucleosome and chromatin‐associated processes. Upregulated proteins in PPC cells are strongly linked to protein ubiquitination, small protein conjugation, amino acid modification, and mitochondrial electron transport‐related pathways (Figure S3C). At the cellular component level, proteins dysregulated by CD63 (Figure S4B) and LAMP2B (Figure S4C) constructs mapped to vesicle and granule lumen compartments, indicating their engagement with the cells’ secretory system. Collectively, these analyses indicate that, while a core cellular proteome remains largely preserved, transient expression of distinct VAD constructs drives construct‐specific proteomic remodeling. These changes converge on nuclear regulatory and endomembrane pathways, processes that are directly linked to protein expression and EV biogenesis.

EVs were isolated from the CCM of transfected and WT cells by differential ultracentrifugation, and sample purity was assessed based on size, concentration, morphology, and canonical EV markers [30]. Nanoparticle tracking analysis (NTA) showed no significant difference in size distribution between EVs from transfected versus WT cells (modal size: 111 vs. 125 nm; Figure 2B). Although the particle concentration per million cells from PTGFRN transfection appeared to be more than that of WT or other constructs, the difference was not statistically significant (Figure 2C). Given that PTGFRN is preferentially sorted into EVs and associates with CD9/CD81‐linked membrane domains involved in trafficking [28], it is plausible that PTGFRN expression may influence EV sorting or release dynamics; however, this was not directly tested here. Similarly, the modal particle size (∼111 ± 10 nm) through all samples showed no significant difference (Figure 2D). Cryo‐transmission electron microscopy (TEM) confirmed spherical morphology and intact bilayer structures of EVs (Figure 2E). Immunoblots verified the presence of CD9 (tetraspanin) and Flotillin‐1, classical EV markers, and a lack of expression of endoplasmic reticulum‐specific marker, GRP94, indicating purity of isolated EV samples (Figure 2F). Proteomic analysis of MS data confirmed the enrichment of EV‐specific markers (CD63, CD9, TSG101, PDCD6IP, CD81, FLOT1, SDCBP, ANXA2, HSP90AA1) in EV samples, while non‐EV proteins (e.g., HSP90B1, CANX, ATP5F1A/B) were more abundant in cell lysates (Figure 2G; Figure S5). Together, these data confirmed that transfection did not introduce artefacts affecting EV purity or physicochemical characteristics.

**FIGURE 3:**
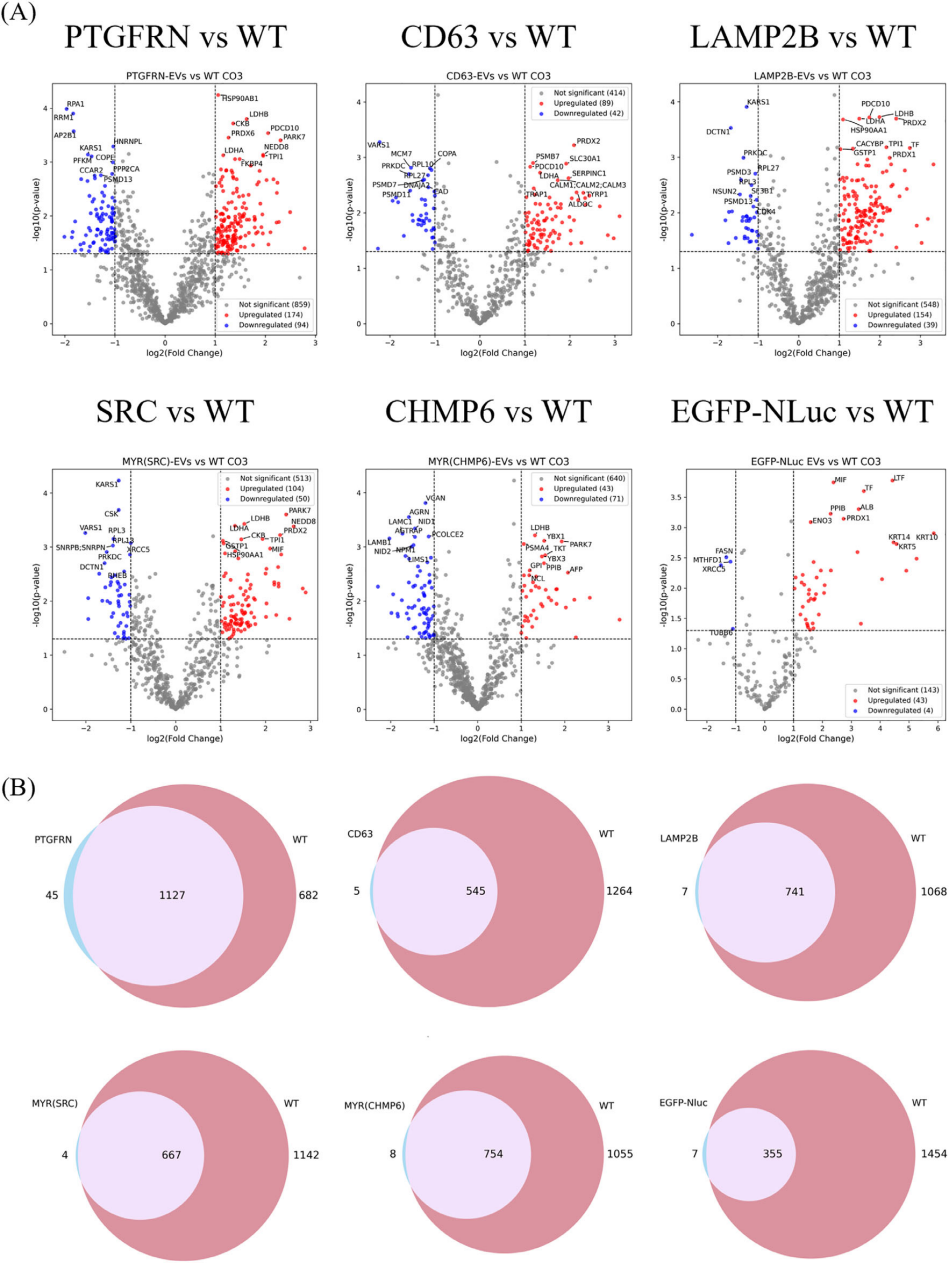
Global proteomic profiling of engineered and wild‐type EVs. Comparative analyses of proteomic profiles were performed to assess transfection‐associated changes in protein abundance. (A) Volcano plots display differential protein expression between engineered and wild‐type EVs, with thresholds set at log_2_ fold change > 1.0 or < −1.0 and *p* < 0.05. (B) Venn diagrams illustrate the distribution of proteins unique to each condition, shared between engineered and wild‐type EVs.

Global proteomics using MS revealed that EVs incorporating different VAD‐fusion proteins shared an enrichment of 10 proteins (ALDOA, LTF, MIF, NME1, PGK1, PPIA, PPIB, PRDX1, TF, TPI1) compared to WT EVs, indicating engineering‐associated changes in vesicular cargo (Figure 3A). However, the EVs engineered with different VAD‐fusion proteins revealed a distinct EV proteomic profile compared to WT EVs; PTGFRN (174 up, 94 down), CD63 (89 up, 42 down), LAMP2B (154 up, 39 down), SRC (104 up, 50 down), CHMP6 (43 up, 71 down), and PPC (43 up, 4 down) (Figure 3A). Comparison of unique versus shared proteins for eEVs and WT EVs revealed that engineering cells using PTGFRN‐fusion proteins stimulated the expression of 45 unique proteins in eEVs, with 1127 shared and 682 WT‐exclusive proteins, while other VAD‐fusion proteins produced similar trends (Figure 3B). To define the biological significance of EV proteome remodeling induced by different VADs, significantly dysregulated EVs proteins from each engineered condition were subjected to grouped gene ontology‐biological processes enrichment analysis relative to WT EVs (Figure S6). Across all conditions, the enriched terms converged on a restricted set of biologically relevant themes such as protein trafficking, membrane organization, adhesion, receptor‐mediated endocytosis, immune‐associated signaling, and metabolism. Accordingly, these pathways are mechanistically linked to EV biogenesis, membrane composition, and host‐cell interaction, indicating that VAD choice selectively reshapes functional EV features while preserving overall vesicular features.

EVs engineered using PTGFRN‐VAD were primarily associated with protein trafficking, adhesion, and receptor‐mediated endocytosis, consistent with the scaffold that supports membrane presentation while promoting EV interaction and uptake‐related features (Figure S6A). Similarly, CD63‐VAD also retained a trafficking‐centered profile and showed additional enrichment in cell‐cell junction assembly, and Ca^2+^‐linked membrane processes, suggesting selective effects on membrane organization and vesicle interaction pathways (Figure S6B). In contrast, LAMP2B‐VAD engineered EVs exhibited strong enrichment for vesicle‐mediated transport, endo‐lysosomal organization, multivesicular body‐lysosome fusion, and receptor‐mediated endocytosis, in line with the established endo‐lysosomal association of LAMP2B and indicating a stronger imprint on intracellular sorting pathways (Figure S6C). EVs engineered with MYR(SRC)‐VAD were enriched for integrin‐mediated signaling, extracellular membrane organization, adhesion, and immune‐associated pathways, consistent with altered membrane‐facing features that may influence tissue interaction and biodistribution (Figure S6D). Notably, EVs with MYR(CHMP6)‐VAD showed strong enrichment for plasma membrane organization and tubulation, adhesion‐related signaling, and membrane remodeling pathways, consistent with the ESCRT‐linked role of CHMP6 in membrane deformation and vesicle formation (Figure S6E). Lastly, even the EVs engineered as PPC showed enrichment of adhesion, endocytosis, and metabolic pathways, indicating changes originating from transient overexpression (Figure S6F). Collectively, these data suggest that although engineered EVs retain a core proteome, each VAD‐fusion protein modulates cargo loading and functional pathways in a distinct manner.

**FIGURE 4:**
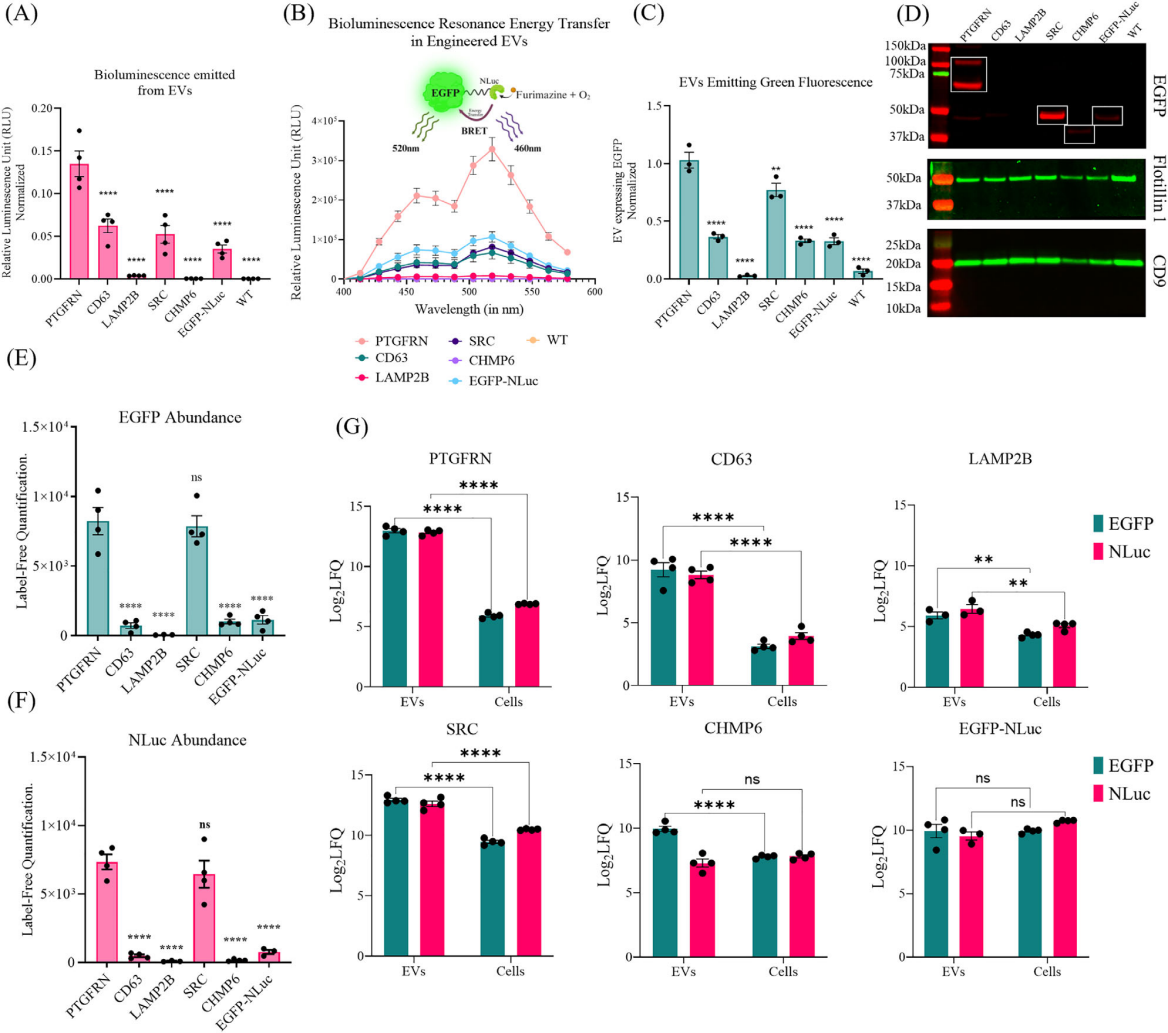
Evaluation of packaging efficiency of EGFP‐NLuc fusion protein into EVs using different VADs. (A) Packaging efficiency of fusion proteins, measured as the NLuc bioluminescence of EVs normalized against the corresponding cell lysate NLuc signal. (B) Spectral luminescence analysis demonstrating bioluminescence and fluorescence emission profiles from EVs packaged with EGFP‐NLuc fusion proteins. (C) Quantification of EGFP‐positive EVs using nano‐flow cytometry (nFCM), normalized against EGFP‐positive cell populations. (D) Immunoblot analysis confirming the expression of EGFP‐NLuc fusion proteins packaged into EVs by different VADs, with EV markers used as loading controls. Flotillin‐1 and CD9 were blotted using separate gels due to their similar molecular weight as fusion proteins. (E, F) Quantitative proteomic analysis of EGFP and NLuc expression in engineered EVs. EGFP (E) and NLuc (F) levels in EVs were quantified by DIA mass spectrometry using a label‐free quantification (LFQ) approach. (G) Quantitative comparison of reporter protein abundance in engineered EVs and source cells. Log_2_‐transformed LFQ values for EGFP and NLuc were compared between EVs and their originating cells to assess enrichment efficiency across different packaging strategies, including vesicle anchoring domains (VADs) and passive loading. Statistical significance was assessed using one‐way ANOVA with multiple comparisons performed using Tukey's tests, with *p* < 0.05 considered significant. ^*^ (*p* ≤ 0.05), ^**^ (*p* ≤ 0.01), ^***^ (*p* ≤ 0.001), ^****^ (*p* ≤ 0.0001).

The reporter cargo packaging efficiency of each VAD in engineered EVs was quantified using multimodal fluorescence and luminescence readouts. We measured the Nanoluciferase (NLuc) bioluminescent signal emitted from the eEVs after treatment with Furimazine, the NLuc chemical substrate. To ensure that the bioluminescent signal originated from the reporter cargo packaged intraluminally inside the eEVs, a protease protection assay was performed in which an equal number of EVs were treated with proteinase and detergent either separately or in combination (Figure S7). Bioluminescent signals from proteinase‐treated EVs were normalized against the corresponding cell lysate luminescent signals to account for variability in the transfection efficiency of different VAD‐fusion protein expressing plasmids (Figure 4A). The PTGFRN‐VAD achieved the highest cargo packaging, followed by CD63 and SRC, whereas PPC (passive loading) outperformed the LAMP2B‐, and CHMP6‐ VADs. To evaluate BRET activity, luminescence measurements at wavelength intervals between 400 and 600 nm were recorded. Luminescence measurements at wavelength intervals between 400 and 600 nm indicated a strong EGFP signal (∼520 nm), confirming the presence of intact EGFP‐NLuc fusion protein. EVs engineered using PTGFRN‐VAD very clearly generated the strongest NLuc (∼460 nm) and EGFP (∼520 nm) signals, followed by PPC EVs (Figure 4B; Figure S8). Similarly, nano‐flow cytometry (nFCM) showed that PTGFRN‐ and SRC‐ fusion proteins resulted in the highest percentage of EGFP‐positive EVs (Figure 4C). Immunoblots confirmed the packaging of EGFP protein cargo in the eEVs for all VADs (Figure 4D). Consistent with luminescence and nFCM results, PTGFRN‐ and SRC‐ fusion proteins exhibited the strongest band intensity (Figure 4D). nFCM measurements of EGFP‐positive EVs were normalized to parent cell fluorescence, confirming comparable expression across constructs and ensuring that observed differences reflect intrinsic cargo loading efficiency rather than variability in expression. Of note, two distinct EGFP‐reactive bands were consistently observed in immunoblots for both EVs and the cell lysate from the cells transfected with the PTGFRN‐fusion protein plasmid. Notably, the PTGFRN construct produced an additional lower‐molecular‐weight EGFP‐reactive band that was absent or minimal in the other VAD groups. This was detectable in both the producer cell lysates (Figure S1C) and the corresponding EVs, suggesting that it originates during intracellular processing of the PTGFRN fusion protein and is subsequently carried into secreted EVs. DIA‐LFQ proteomic analysis of EVs further reinforced these observations, as EGFP and NLuc levels were highest in PTGFRN EVs, followed by SRC EVs, while passively packaged EVs yielded EGFP and NLuc protein levels comparable to CD63, LAMP2B, and CHMP6 EVs (Figure 4E,F). DIA‐LFQ proteomic analysis further revealed significantly higher enrichment of EGFP and NLuc in eEVs packaged through VADs compared with their producer cells, whereas passively packaged eEVs showed no enrichment, underscoring the selective cargo loading capacity of each VAD (Figure 4G).

**FIGURE 5:**
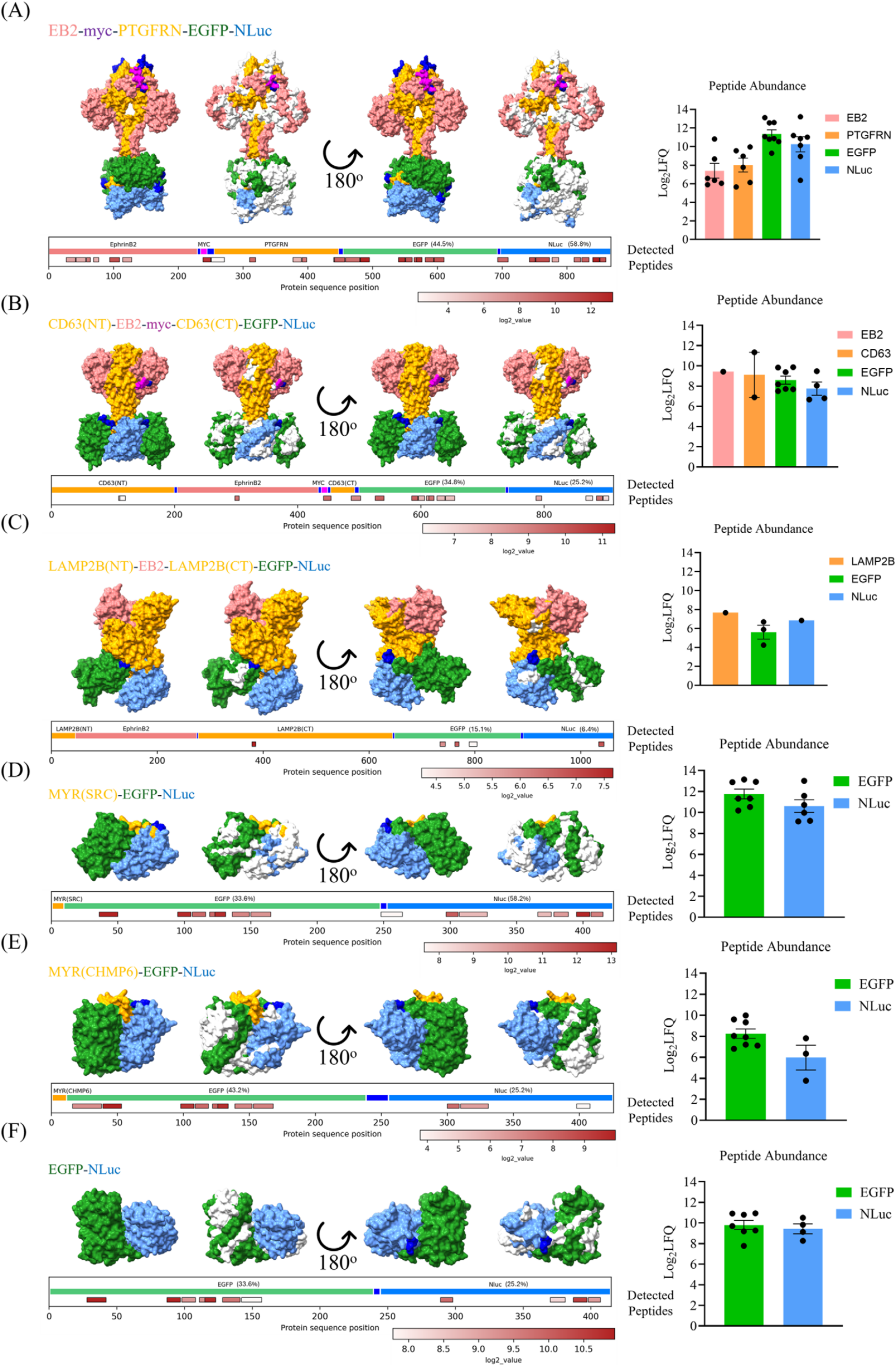
Peptide level characterization of fusion proteins in engineered EVs. 3D chimeric models and linear sequence maps of different VAD‐cargo fusion proteins, (A) EB2‐myc‐PTGFRN‐EGFP‐NLuc, (B) CD63(NT)‐EB2‐myc‐CD63(CT)‐EGFP‐NLuc, (C) LAMP2B(NT)‐EB2‐LAMP2B(CT)‐EGFP‐NLuc, (D) MYR(SRC)‐EGFP‐NLuc, (E) MYR(CHMP6)‐EGFP‐NLuc, and (F) EGFP‐NLuc, were overlaid with peptides identified by DIA‐mass spectrometry. Detected peptides are highlighted in white on the structural models and mapped to the linear protein sequences with color intensity indicating log_2_ LFQ abundance. Quantitative comparison of peptide abundance is shown as bar plots of log_2_‐transformed LFQ values, with symbols above the bars denoting the number of unique peptides identified for each construct. Statistical significance was assessed using one‐way ANOVA with multiple comparisons performed using Tukey's test.

To further resolve cargo packaging on the peptide level, we mapped MS‐identified peptides onto 3D chimeric models and linear sequences of VAD‐EGFP‐NLuc fusion proteins (Figure 5A–F). Peptides were detected across targeting ligand, EB2 (in PTGFRN and CD63), anchoring domains (in PTGFRN, CD63, and LAMP2B), and reporter cargo EGFP and NLuc, confirming expression of the engineered fusion protein in eEVs. Sequence mapping highlighted the domain‐specific abundance, with PTGFRN‐VAD yielding broad and intense coverage of EGFP (44.5% sequence coverage) and NLuc (58.8% sequence coverage) unique peptides (Figure 5A), whereas CD63 (34.8%—EGFP and 25.2%—NLuc) and LAMP2B (15.1%—EGFP and 6.4%—NLuc) displayed sparse coverage with significantly lower intensities (Figure 5B,C). In the packaging mediated by myristoylation, SRC‐VAD yielded a comparable abundance of unique cargo peptides to PTGFRN‐VAD (Figure 5D), while CHMP6‐VAD displayed significantly lower abundance (Figure 5E). Sequence coverage analysis for unique cargo peptides showed that SRC‐VAD achieved NLuc coverage (58.2%) comparable to PTGFRN (Figure 5D), while CHMP6‐VAD (43.2%) achieved EGFP coverage similar to PTGFRN (Figure 5E). Notably, reporter cargo, EGFP‐NLuc passively packaged inside eEVs exhibited abundance and sequence coverage comparable to, or in some cases greater than, that of VADs such as CD63, LAMP2B, or CHMP6 (Figure 5F). Collectively, the peptide level analyses complemented the bulk proteomic readouts and established PTGFRN as the most effective VAD for promoting selective cargo packaging in eEVs.

### 2.2 Scalable Production of Engineered EVs From Biomanufacturing Relevant Cell Lines

As a first step toward upscaling production of EVs with exogenous cargo, we evaluated the yield, proteomic composition, and packaging efficiency of EVs produced by five bio‐pharmaceutically relevant suspension cell lines (ExpiCHO, Lonza‐CHO (CHOK1 GS‐KO), CHO‐S, FLP‐IN‐CHO, and Expi293). Among these, the three closely related CHO cell lines (ExpiCHO, Lonza‐CHO, and CHO‐S) were used to assess how differences in their expression systems influence EV yield and cargo, whereas FLP‐IN‐CHO was included for its ability to integrate transgenes at a defined genomic locus, enabling stable and controlled expression. Expi293 cells were included for their human origin and distinct glycosylation patterns, which influence EV membrane protein composition.

To facilitate a comparison of eEV production by the five suspension cell lines, we selected the truncated PTGFRN VAD (which performed well in adherent HEK293T cells) for loading EGFP reporter cargo. To produce eEVs packaged with EGFP, the five cell lines were transiently transfected with an EB2‐PTGFRN‐EGFP plasmid (Plasmid #7, Table 1).

**FIGURE 6:**
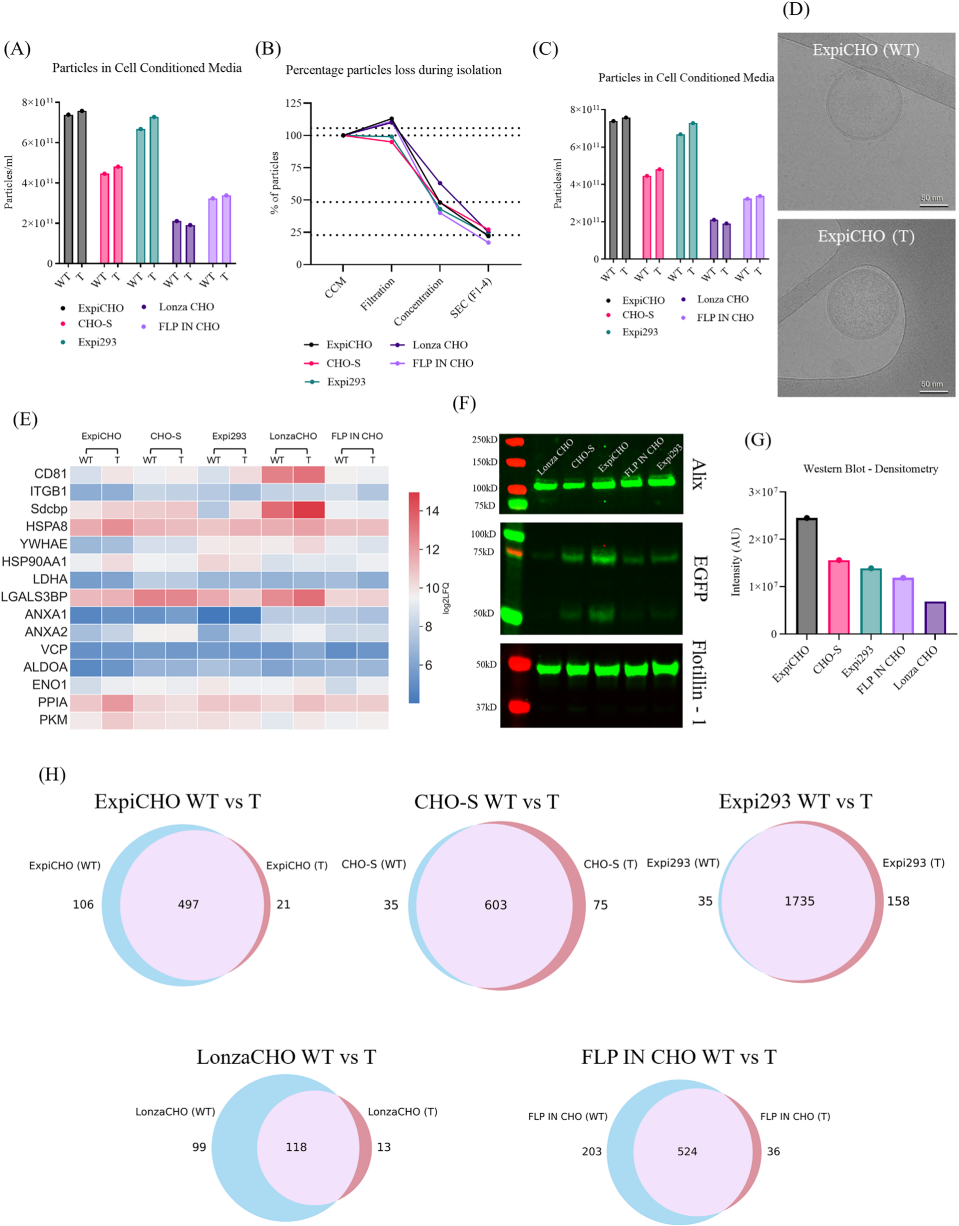
Scalable production of Engineered EVs from biopharmaceutically relevant cell lines. (A) Nanoparticle tracking analysis quantifying total particle concentration in unprocessed conditioned culture medium from transfected (T) and untransfected (WT) cells. (B) Evaluation of particle recovery at each step of the EV isolation process: post‐filtration, post‐concentration, and post‐size exclusion chromatography. (C) NTA‐based quantification of particle concentration in SEC‐enriched EV preparations from different cell lines. (D) Cryo‐transmission electron microscopy (cryo‐TEM) images showing lipid bilayer structures and spherical morphology of isolated EVs. (E) Proteomic profiling of EVs using data‐independent acquisition (DIA) mass spectrometry was used for the identification of classical EV markers filtered through the ExoCarta database. (F, G) Evaluation of the packaging efficiency of EGFP fusion protein into EVs derived from various biopharmaceutically relevant cell lines. (F) Immunoblot analysis confirming the expression of EGFP fusion proteins in EVs derived from various cell lines, with EV markers used as loading controls. Alix and Flotillin‐1 were blotted using separate gels, due to their similar molecular weight as fusion proteins. (H) Densitometry quantification of the EGFP reactive bands from the immunoblot. (G) Global proteomic analysis of EVs to assess transfection‐induced changes across different producer cell lines. Venn diagram depicting comparative analysis of total, shared, and uniquely identified proteins in EVs derived from transfected (T) and untransfected (WT) cells, based on data‐independent acquisition mass spectrometry.

CCM from both transfected (T) and untransfected (WT) cells was harvested, and total particle counts from unprocessed CCM were determined by NTA (Figure 6A), forming a baseline for evaluating total particle loss at each step of the EV isolation process. CCM from ExpiCHO and Expi293 cells showed the highest particle yields, consistent with their known high cell densities [76] (Figure 6A). Lonza‐CHO produced the fewest particles of all the 5 cell lines, consistent with its low growth density [77] (Figure 6A). EVs were isolated by size exclusion chromatography (SEC), which led to ∼50% particle loss after concentration and ∼75% cumulative loss post‐enrichment (Figure 6B).

The purity of EV samples was evaluated by assessing their concentration, morphology, size, and protein‐specific markers, according to MISEV2023 guidelines [30]. NTA and protein quantification of SEC fractions indicated the highest particle‐to‐protein ratio in the first four fractions, which were pooled for downstream analyses (Figure S9A).

Following enrichment, ExpiCHO and Expi293 again yielded the highest EV particle concentrations, with Lonza‐CHO the lowest (Figure 6C). Cryo‐TEM confirmed the spherical morphology and lipid bilayer structure of EVs (Figure 6D; Figure S9B). DIA ‐LFQ cross‐referenced with ExoCarta validated the presence of EV‐specific markers and showed consistent expression between WT and T groups across cell lines (Figure 6E).

Immunoblotting confirmed the presence of EGFP fused to PTGFRN within isolated EVs with native EV protein markers such as Flotillin‐1 and Alix [30] serving as EV loading controls (Figure 6F,G). A strong ∼75 kDa EGFP band was observed for ExpiCHO‐derived EVs, indicating efficient packaging, while signal intensity was weaker or absent in EVs from other lines.

Global proteomic profiling revealed transfection‐induced changes in EV cargo (Figure 6H). A total of 230, 624, 713, 763, and 1928 proteins were identified from EVs derived from Lonza‐CHO, ExpiCHO, CHO‐S, FLP‐IN‐CHO, and Expi293, respectively. ExpiCHO (T) EVs showed a stable proteomic profile, with 21 unique proteins compared to 106 in WT EVs, and 497 proteins shared between both. Transfection caused greater shifts in CHO‐S EVs (75 unique proteins in T versus 35 in WT). Expi293 EVs had the largest proteome, likely reflecting the complexity of the *Homo sapiens* proteome relative to *Cricetulus griseus* (Chinese Hamster), with 158 unique proteins in T versus 35 in WT. Lonza‐CHO EVs displayed only 13 unique proteins post‐transfection, but the total proteome was comparatively smaller. FLP‐IN‐CHO EVs mirrored ExpiCHO, with 36 unique proteins in T versus 203 in WT, the majority being shared proteins.

### 2.3 Generation of Stable Cell Lines to Produce Engineered EVs Packaged With EGFP‐NLuc

ExpiCHO cells were selected to develop scalable production of ovarian cancer targeting eEVs due to their superior packaging efficiency and minimal disruption of native EV proteome composition. We generated stable pools of two separate ExpiCHO lines expressing EGFP‐NLuc fusion protein tethered to truncated PTGFRN with randomly integrated copies of linearized plasmid DNA (Plasmid #1 and #8, Table 1); one pool expressed EB2‐PTGFRN‐EGFP‐NLuc (EB2^+ve^, Ephrin‐B2 ligand tethered to N‐terminus of PTGFRN) and a second pool expressed PTGFRN‐EGFP‐NLuc (EB2^−ve^, lacking the ligand). The EB2^+ve^ cell line was designed to display the EB2 ligand on the outer surface of EVs incorporating the fusion protein. Confocal imaging confirmed EGFP expression in the stable cells (Figure S10A) and proportion of EGFP‐expressing cells (EB2^+ve^ ∼71%, EB2^−ve^ ∼74%) were determined by flow cytometry (Figure S10B). Nanoluciferase assays showed comparable bioluminescence for EB2^+ve^ and EB2^−ve^ cells; this indicated similar transgene expression levels, suggesting that the copy number of integrated transgenes was equal or close to equal for the two stable cell lines (Figure S10C). Immunoblotting of cell lysates revealed two distinct EGFP‐reactive bands in EB2^+ve^ cells and a single prominent band in EB2^−ve^ cells (Figure S7D). This could indicate processing of the EB2 extraluminal domain, possibly by metalloproteases [28].

Comparative analyses of global proteomic profiles were performed to assess changes associated with stable integration of the constructs (Figure S10E). Differentially expressed proteins were defined by log2fold‐change thresholds (>1.0 for upregulation, ←1.0 for downregulation) with a significance cut‐off of p < 0.05. Relative to WT cells, 27 proteins were upregulated, and 101 were downregulated in stably integrated cells (Figure S10E,F). Between the two stable lines, EGFP and NLuc were significantly downregulated in EB2^+ve^ cells, whereas PDHX and SFXN4 were upregulated (Figure S10E). Interestingly, this difference in EGFP and NLuc expression was not evident in NanoLuc assays, flow cytometry, or western blotting, suggesting that DIA‐LFQ captured subtle variations in protein abundance that were not detected by conventional assays. Comparative analysis of shared and unique proteins revealed 2257 proteins common in all conditions, with 83 proteins unique to EB2^−ve^, 181 unique to EB2^+ve^, and 59 unique to WT cells (Figure S10G). Additionally, 410 proteins were shared between EB2^+ve^ and EB2^−ve^, while only 54 and 14 proteins were shared between EB2^+ve^ and WT, and EB2^−ve^ and WT, respectively (Figure S10G). These results suggest that while a large core proteome is retained, stable integration drives distinct remodeling of cellular proteins. Enrichment analysis of significantly dysregulated proteins revealed a shared proteomic signature in both EB2^+ve^ and EB2^−ve^ stable cells relative to WT cells (Figure S11A,B). In both groups, upregulated proteins were enriched for nucleosome and chromatin assembly and nuclear compartments, whereas downregulated proteins mapped predominantly to endocytosis, membrane organization, actin and cytoskeletal regulation, focal adhesion, and cell junction pathways, together with membrane raft and intracellular membrane‐bound organelle terms. This suggests that stable engineering induces coordinated remodeling of both nuclear and membrane‐associated pathways, where the introduction of exogenous DNA potentially impacts chromatin, transcriptional, and translational processes, while the PTGFRN scaffold preferentially influences membrane dynamics, adhesion, and trafficking related pathways. Notably, the overall enrichment profiles were highly similar between both the stable cell lines, suggesting that the major proteomic changes are driven by the engineered scaffold, with EB2 contributing only modest secondary effects related to host‐interaction biology.

EVs were harvested from the CCM of stably integrated and WT ExpiCHO cells. Cells were continuously monitored for viable cell density, showing steady growth with no significant differences between cell treatments (Figure S12A). NTA of aliquots from unprocessed CCM confirmed a progressive increase in particle concentrations, also without significant differences among treatments (Figure S12B). EVs were isolated from concentrated CCM by SEC, with ten fractions collected to maximize purity over yield. NTA and protein quantification demonstrated that EVs were enriched in the first four fractions, which also exhibited the highest Nanoluciferase bioluminescence signal, whereas a negligible signal was detected in WT EVs (Figure S12C,D).

**FIGURE 7:**
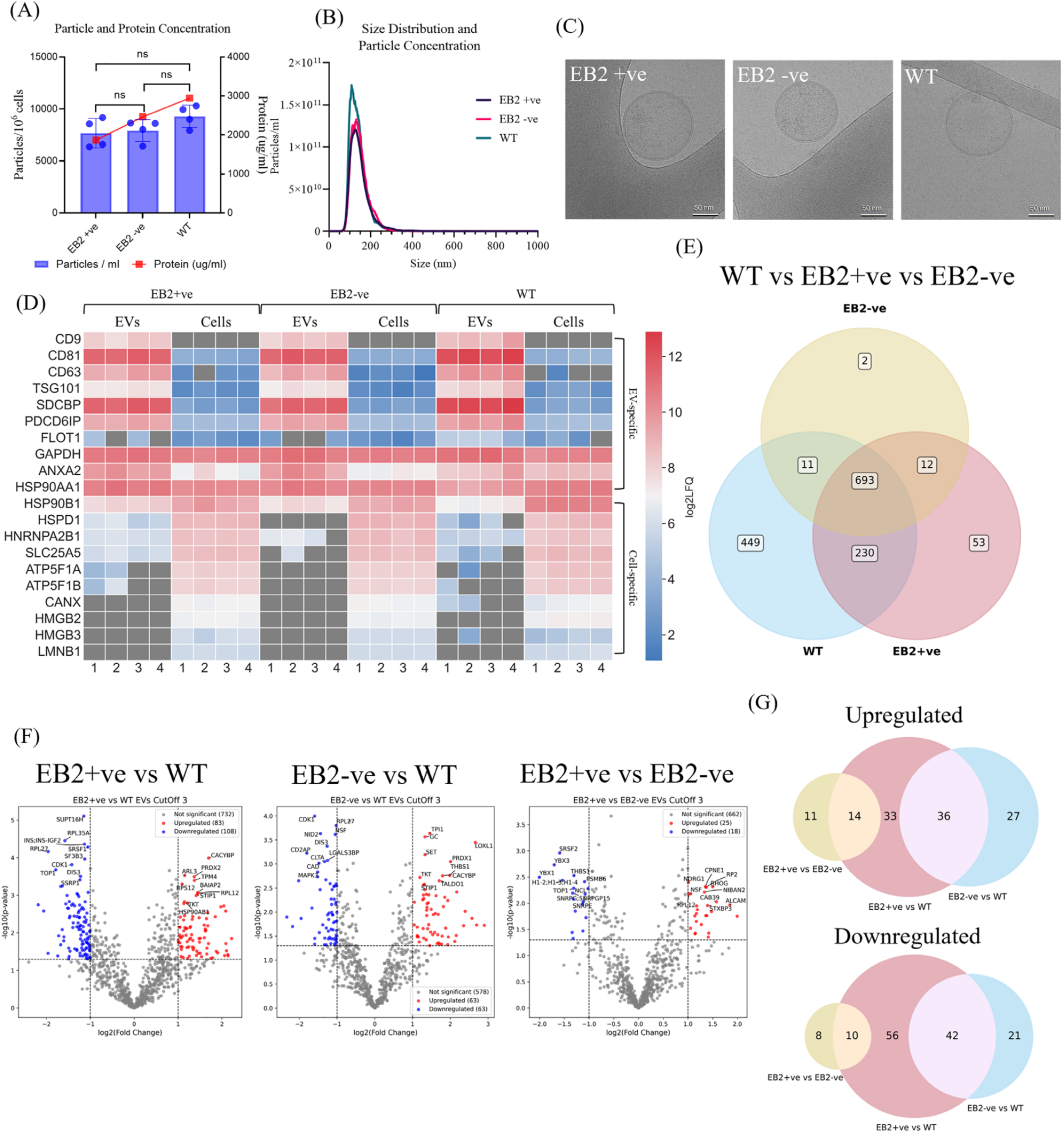
Comprehensive characterization of EVs from wild‐type and stably engineered cell lines. (A, B) Nanoparticle tracking analysis and protein quantification of EVs isolated from engineered (EB2^+ve^, EB2^−ve^) and wild‐type (WT) cells, determining particle concentration per million cells, modal size distribution, and protein concentration. (C) Cryo‐transmission electron microscopy (cryo‐EM) images illustrating vesicles with intact, spherical morphology and a clearly defined lipid bilayer. (D) Proteomic characterization of EVs and corresponding cell lysates to assess the expression and relative abundance of EV‐associated and non‐EV proteins. (E–G) Global proteomic profiling of engineered and wild‐type EVs. (E) Global proteomic profiling of EVs to identify proteins shared among all EV populations, unique to specific groups, and common between selected pairs. (F) Comparative analyses of proteomic profiles were performed to assess stable integration of specific changes in EV‐protein abundance. (G) Venn diagrams showing the overlap of differentially expressed proteins between engineered EV populations. Statistical significance was assessed using one‐way ANOVA with multiple comparisons performed using Tukey's test.

The first four SEC fractions were pooled, concentrated, and characterized for size, particle concentration, morphology, and protein markers, following MISEV2023 guidelines [30]. NTA of concentrated fractions from the control and engineered cells indicated no significant differences in the concentration of particles, and the modal particle size was 121 ± 10 nm (Figure 7A,B). Consistent with the NTA, total protein did not differ significantly between the three treatments (Figure 7A). Cryo‐TEM images of particles from each treatment showed the presence of intact spherical vesicles with a well‐defined lipid bilayer structure, consistent with the expected morphology of EVs (Figure 7C). Proteomic analysis confirmed enrichment of EV‐specific markers (i.e. CD63, CD9, TSG101, PDCD6IP, CD81, GAPDH, FLOT1, SDCBP, ANXA2, and HSP90AA1) in EV samples, while non‐EV proteins (i.e. HSP90B1, HSPD1, HNRNPA2B1, SLC25A5, ATP5F1A, ATP5F1B, CANX, HMGB2, HMGB3, and LMNB1) were more abundant in cell lysates (Figure 7E; Figure S13). Together, these data confirmed that stable integration did not introduce artefacts affecting characteristics of EVs.

Global proteomic analysis of EVs identified 693 proteins shared across all conditions, with 449 unique to WT, 53 unique to EB2^+ve^, and 2 unique to EB2^−ve^ EVs (Figure 7E). An additional 230 proteins were shared between WT and EB2^+ve^ EVs, whereas only 11 and 12 proteins were shared between EB2^−ve^ and WT, and EB2^−ve^ and EB2^+ve^, respectively (Figure 7E). Relative to WT EVs, 83 proteins were significantly upregulated and 108 downregulated in EB2^+ve^, while EB2^−ve^ EVs showed 63 upregulated and 63 downregulated proteins (Figure 7F). Relative to EB2^−ve^ EVs, EB2^+ve^ EVs exhibited 25 upregulated and 18 downregulated proteins, highlighting ligand‐dependent cargo differences (Figure 7F). Across both engineered EV populations, 36 proteins were commonly upregulated, and 42 were commonly downregulated relative to WT (Figure 7G). GO enrichment analysis of significantly dysregulated EV proteins showed that both EB2^+ve^ and EB2^−ve^ EVs shared a core engineered signature relative to WT EVs, dominated by translation, gene expression, and DNA metabolic pathways, indicating that much of the EV proteome remodeling arises from the common PTGFRN‐induced engineering (Figure S14A–C). EB2^−ve^ additionally exhibited broader enrichment in exosomal secretion, nuclear membrane organization, ECM and integrin‐related pathways, inflammatory signaling, and ubiquitin‐related catabolism, highlighting the stronger effects of vesicle release and matrix interaction pathways (Figure S14A). In contrast, EB2^−ve^ EVs displayed a restricted profile enriched for biosynthetic trafficking and mucosal immune response pathways (Figure S14B). Direct comparison between EB2^+ve^ and EB2^−ve^ EVs further highlighted differences in intracellular transport, membrane localization, actin‐based vesicle trafficking, chromatin‐associated processes, and immune response terms, indicating that expression of EB2 ligand selectively tunes EV cargo composition instead of redefining EV functionalities (Figure S14C).

**FIGURE 8:**
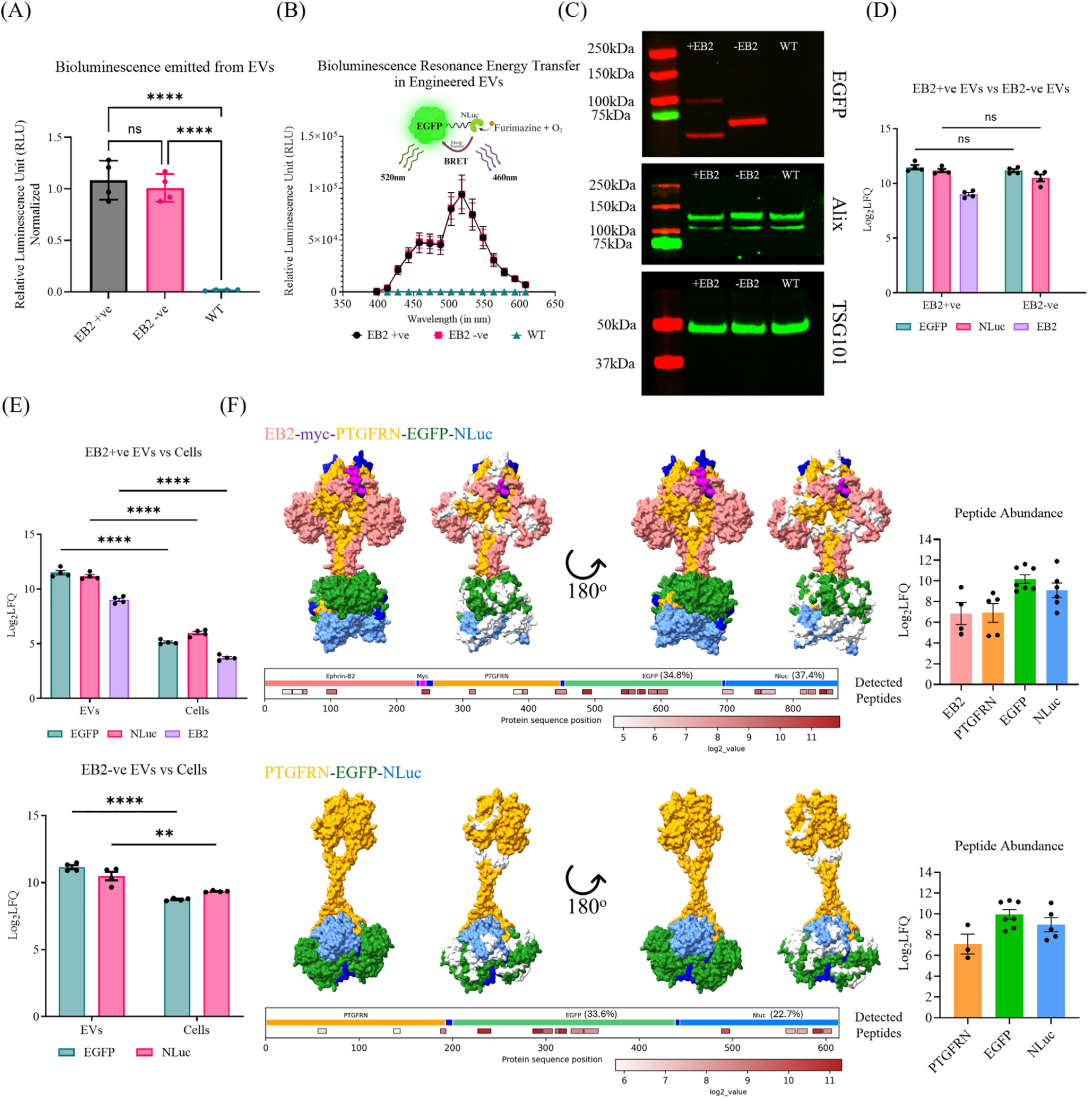
Assessment of reporter protein packaging and characterization of engineered EV cargo. (A) Bioluminescence quantification of intraluminal NLuc activity in EVs following proteinase treatment, normalized to corresponding cell lysates. (B) Spectral luminescence analysis confirming intraluminal proximity‐based energy transfer between NLuc and EGFP inside EVs. (C) Immunoblot analysis of EVs showing EGFP‐NLuc fusion protein expression, with Alix and TSG101 as EV‐specific loading controls. Alix and TSG101 were blotted using a separate gel, due to their similar molecular weight as fusion proteins (D) Quantitative proteomic analysis of EGFP, NLuc, and Ephrin‐B2 expression in engineered EVs. EGFP, NLuc, and EB2 levels in EVs were quantified by DIA mass spectrometry using a label‐free quantification (LFQ) approach. (E) Quantitative comparison of reporter protein abundance in engineered EVs and source cells. Log_2_‐transformed label‐free quantification (LFQ) values for EGFP and NLuc were compared between EVs and their originating cells to assess enrichment efficiency across different engineered EV groups. (F) Peptide‐level characterization of EB2^+ve^ and EB2^−ve^ PTGFRN fusion proteins in engineered EVs 3D chimeric structural models and linear sequence maps of EB2‐myc‐PTGFRN‐EGFP‐NLuc (EB2^+ve^) and PTGFRN‐EGFP‐NLuc (EB2^−ve^) overlaid with peptides identified using DIA‐mass spectrometry. Detected peptides are highlighted in white on the structural models. Linear sequence maps highlighted domain‐specific peptide abundance, color‐coded by log_2_ LFQ intensity. Quantitative comparison of identified peptides is shown as bar plots of log_2_ LFQ values, with symbols above bars indicating the number of unique peptides identified for each construct. Statistical significance was assessed using one‐way ANOVA with multiple comparisons performed using Tukey's tests, with *p* < 0.05 considered significant. ^*^ (*p* ≤ 0.05), ^**^ (*p* ≤ 0.01), ^***^ (*p* ≤ 0.001), ^****^ (*p* ≤ 0.0001).

Nanoluciferase bioluminescence was measured to confirm the presence of EGFP‐NLuc fusion protein packaged within EVs. To ensure that signals originated from encapsulated proteins, a protease protection assay was performed in which an equal number of EVs were treated with proteinase and detergent either separately or in combination (Figure S15A). Bioluminescence from proteinase‐treated EVs was normalized against total luminescence from corresponding cell lysates to account for variations in cellular expression of transgenes (Figure 8A). No significant difference was observed in NLuc bioluminescence between EB2^+ve^ and EB2^−ve^ EVs, indicating comparable (equimolar) cargo loading. To evaluate BRET activity, luminescence measurements at wavelength intervals between 400 and 600 nm were recorded (Figure 8B; Figure S15B–D). A strong EGFP‐specific signal (∼520 nm) confirmed the presence of intact, uncleaved EGFP‐NLuc fusion proteins. Accordingly, as for NLuc bioluminescence, the EB2^+ve^ and EB2^−ve^ EVs showed no significant difference in EGFP fluorescence (Figure 8B). Immunoblotting further confirmed the presence of EGFP within EVs, with native EV protein markers such as Alix and TSG101 [30] serving as EV‐specific markers (Figure 8C). EB2^−ve^ EVs displayed a single, distinct EGFP band, whereas the EB2^+ve^ group consistently exhibited two EGFP‐reactive bands, we presume that EGFP expression may be distributed across two EV populations in EB2^+ve^ PTGFRN‐mediated packaging.

We further quantified EGFP, NLuc, and EB2 in eEVs using DIA ‐LFQ analysis. No significant differences were observed between the abundance of EGFP and NLuc in the eEVs (Figure 8D). Compared with eEVs producing stable cells, all three proteins were significantly enriched in eEVs, demonstrating once again that PTGFRN enabled efficient and selective cargo loading (Figure 8E). To further resolve cargo packaging at the peptide level, we mapped MS‐identified peptides onto the 3D chimeric models of EB2^+ve^ and EB2^−ve^ PTGFRN‐fusion proteins (Figure 8F). The sequence mapping confirmed a highly overlapping peptide distribution for reporter cargo, whereas the LFQ analysis showed comparable abundance of EGFP and NLuc‐derived peptides between both the eEV groups. The sequence coverage analysis supported these observations as both groups exhibited broad and intense coverage of EGFP (34.8%—EB2^+ve^ and 33.6%—EB2^−ve^) and NLuc (37.4%—EB2^+ve^ and 22.7%—EB2^−ve^) unique peptides.

Together, these results indicate that EB2 ligand display on the surface of eEVs does not alter the efficiency of EGFP‐NLuc packaging.

### 2.4 Spatiotemporal Distribution of Engineered EVs in Ovarian Tumor PDXs

**FIGURE 9:**
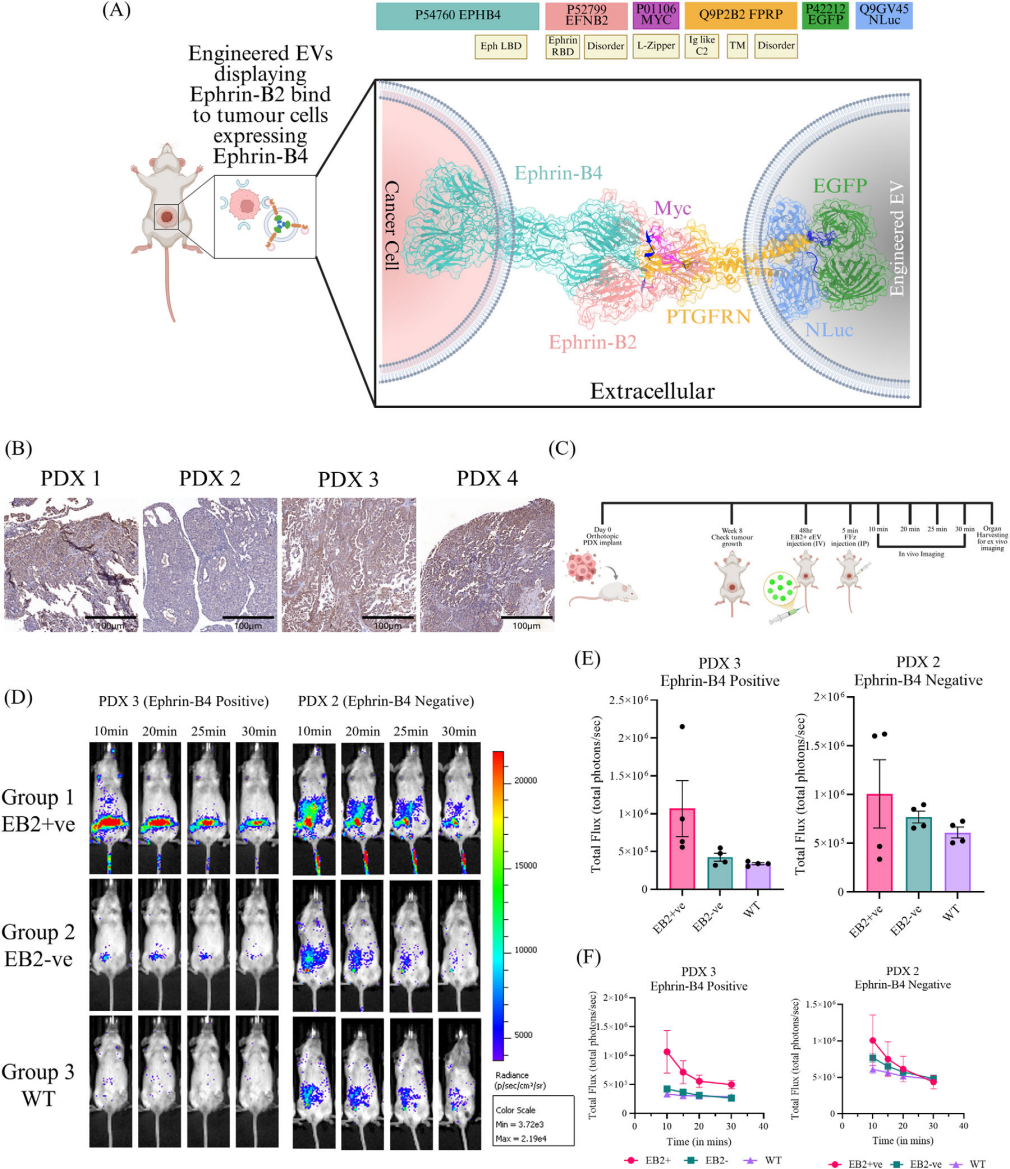
In vivo spatiotemporal distribution of engineered EVs (eEVs) in ovarian cancer PDX models. (A) Schematics of engineered EVs displaying Ephrin‐B2 ligand interact with tumor cells expressing Ephrin‐B4 receptor (B) IHC analysis of EphB4 expression in four high‐grade serous ovarian cancer PDX models revealed surface localization in PDX3, cytosolic expression in PDX1 and PDX4, and no expression in PDX2. (C) Schematic of experimental workflow: tumor‐bearing PDX3 (EphB4^+^) and PDX2 (EphB4^−^) mice received 100 µg EGFP‐NLuc‐loaded eEVs via tail vein injection, followed by intraperitoneal fluorofurimazine (FFz). (D) Representative IVIS images over 30 min for total luminescence (open filter) detection. (E) Quantification of total photon flux showed higher signals for EB2^+^^ve^ eEVs in PDX3 with peritoneum‐localized accumulation, versus diffuse distribution in PDX2. (F) Time‐course radiance analysis indicated progressive signal decay.

To assess the in vivo spatiotemporal distribution of eEVs in ovarian cancer, four PDX models were established using high‐grade serous ovarian tumor tissue engrafted into female NSG mice (Figure 9A). Immunohistochemical (IHC) screening of tumors for surface‐specific expression of receptor tyrosine kinase, Eph receptor‐B4 (EphB4), confirmed surface expression in PDX3 and cytosolic expression in PDX1 and PDX4; PDX2 was scored negative (Figure 9B). IHC of non‐cancerous tissues from multiple organs confirmed EphB4 expression in the lungs (Figure S16).

To evaluate EphB4‐specific eEV targeting, tumor slurries from PDX3 and PDX2 were injected intraperitoneally into separate cohorts (*n* = 4). Once tumors were established, mice received 100 µg of bioluminescent eEVs carrying EGFP‐NLuc and WT EVs via tail vein injection, followed by intraperitoneal administration of the fluorofurimazine (FFz) substrate (Figure 9C). In vivo imaging was performed using IVIS over 30 min, capturing total luminescence (open filter) (Figure 9D; Figure S17), NLuc bioluminescence (500 nm) (Figure S18), and EGFP fluorescence (520 nm) (Figure S19).

Group 1 mice (EB2^+ve^ eEVs) exhibited higher total flux across all channels compared to Group 2 (EB2^−ve^ eEVs) and Group 3 (WT EVs) mice (Figure 9E). In PDX3‐treated mice, the luminescence signal localized predominantly to the lower peritoneum, presumably indicating targeted accumulation in EphB4‐positive tissue (Figure 9D). This pattern also aligns with the physiological localization of orthotopic tumor implants, as ovarian cancer tumors will disseminate to the peritoneal surface [78]. In contrast, signals in PDX2 mice (negative for EphB4 expression) were diffusely distributed throughout the peritoneal cavity, suggesting nonspecific dissemination (Figure 9D). A steady decline in radiance was observed over time, as expected for metabolic clearance of the Nanoluciferase substrate (FFz) by hepatic and renal systems, in line with manufacturer guidelines (Promega, USA) and a previous study [79] (Figure 9F).

**FIGURE 10:**
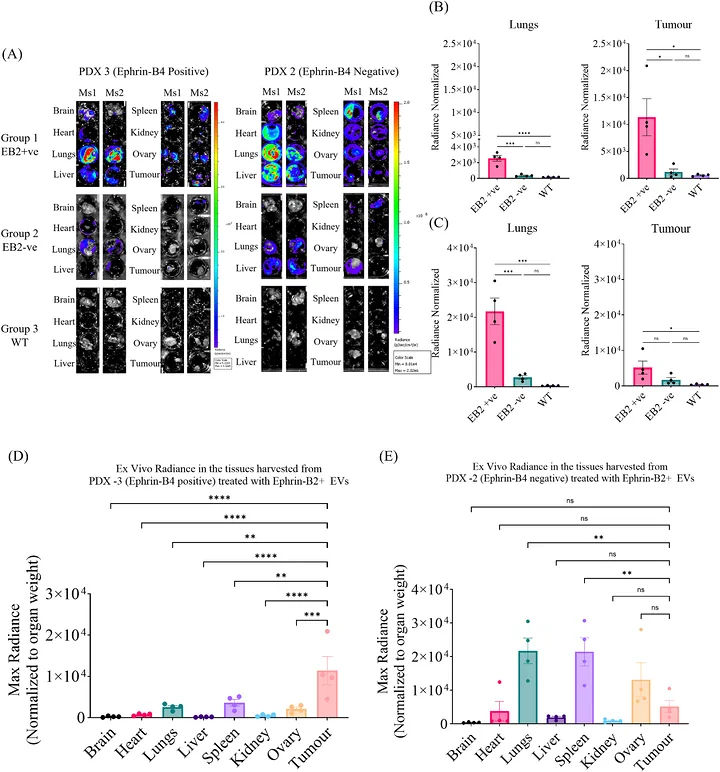
Ex vivo biodistribution of engineered EVs (eEVs) in ovarian cancer PDX models. (A) Representative ex vivo IVIS images of organs (brain, heart, lungs, liver, spleen, kidney, ovary, tumor) harvested from PDX3 (EphB4^+^) and PDX2 (EphB4^−^) mice 40 mins post systemic administration of EGFP‐NLuc loaded eEVs and FFz treatment. (B, C) Quantification of total radiance per organ, showing higher signals for EB2^+^^ve^ eEVs in multiple tissues compared to EB2^−ve^ and WT EVs. (D, E) Normalized radiance (per milligram of tissue) highlighting enriched accumulation of EB2^+^ eEVs in PDX3 tumors, whereas PDX2 tumors showed no selective enrichment. Statistical significance was assessed using one‐way ANOVA with multiple comparisons performed using Tukey's tests, with *p* < 0.05 considered significant. ^*^ (*p* ≤ 0.05), ^**^ (*p* ≤ 0.01), ^***^ (*p* ≤ 0.001), ^****^ (*p* ≤ 0.0001).

To assess organ‐specific biodistribution of eEVs, the brain, heart, lungs, liver, spleen, kidney, ovaries, and tumor were harvested from mice, immersed in FFz, and imaged ex vivo for luminescence (Figure 10A; Figure S20). For both PDX2 and 3 mice, organs from Group 1 animal models showed significantly higher radiance than those from Groups 2 and 3 (Figure 10B; Figure S21A,B). In PDX3‐treated mice, ex vivo radiance emitted by lung tissue was significantly higher than for other organs, including tumor tissue, consistent with the known expression of EphB4 in pulmonary tissue (Figures S16 and S21C). However, this signal was not observed in vivo, which may be attributed to the limited diffusion of the FFz substrate across the diaphragm following intraperitoneal administration [80]. In contrast, in PDX2 mice, ex vivo radiance from lungs was significantly higher than from organs such as the brain, heart, kidney, ovary, and tumor, but was comparable to liver and spleen, suggesting a broader nonspecific distribution pattern (Figure S21D). To account for size‐dependent variability, radiance values were normalized to organ mass, which is a standard practice normally performed in biodistribution studies [26, 81]. In PDX3 mice, normalized radiance was significantly higher in tumor tissue than in all other organs, supporting selective accumulation of EB2^+ve^ eEVs (Figure 10B,D). Conversely, in PDX2 mice, tumor‐normalized radiance was comparable to or lower than that of other organs, consistent with the absence of EphB4‐mediated targeting in this model (Figure 10C,E).

## 3 Discussion

Ovarian cancer remains the seventh most common gynecological malignancy worldwide and the sixth leading cause of cancer‐related mortality among women [1, 82]. Its clinical management is hindered by heterogeneity of subtypes, lack of early detection biomarkers, and limited efficacy of existing therapies, underscoring the need for innovative approaches to enhance patient outcomes [5]. Among emerging modalities, extracellular vesicles have garnered significant interest as therapeutic delivery vehicles due to their biocompatibility, low immunogenicity, and their ability to shield and deliver cargo across biological barriers [33, 34]. These characteristics position EVs as a next‐generation platform for targeted therapeutic delivery [83].

In this study, we systematically evaluated vesicle‐anchoring domains (VADs) and cell lines used in biopharmaceutical manufacturing to develop a scalable system for the production of engineered EVs packaged with exogenous molecular cargo. We assessed six packaging strategies, including five VADs and a passive packaging control, using a dual‐reporter fusion protein (EGFP‐NLuc) that enabled cargo‐protein quantification through detection of both fluorescent and bioluminescent signals. A truncated version of PTGFRN [28], a single‐pass transmembrane protein, consistently outperformed other VADs in packaging efficiency of EVs produced by HEK293T cells. Our comparative analysis of VADs reinforced the work of Dooley et al. [28], who first reported the dual functionality of the truncated N‐terminal variant of PTGFRN (∆687 PTGFRN) [28], and we further confirmed that this PTGFRN protein variant functions well in ExpiCHO cells as an endogenous sorting motif for packaging of exogenous cargo within EV lumen. Of note, however, several studies have previously reported effective EV cargo loading using CD63, LAMP2B, and CHMP6 as scaffolds [36, 38, 39, 40, 42]. We therefore evaluated them in our system but did not replicate those reported outcomes, suggesting that differences may arise from experimental context or the absence of direct comparison to scaffold‐like PTGFRN.

In contrast, canonical EV markers like CD63, despite their prevalence in EV research, demonstrated only moderate packaging efficiency in our experiments. This might be explained by the findings of Ai et al. (2024) that endosomal routing via the YxxΦ motif can divert CD63 toward lysosomal degradation [84]. Similarly, LAMP2B, a frequently used transmembrane scaffold, exhibited poor packaging efficiency, likely due to lysosomal processing [36, 42, 85, 86, 87]. Interestingly, EGFP‐NLuc overexpressed in soluble cytosolic form exhibited EV packaging efficiency comparable to that of several VAD‐fused variants like CD63, LAMP2B, and CHMP6. The packaging of such soluble cytosolic protein is often stochastic and is not driven by any endogenous sorting machinery, which could often lead to heterogeneous or unpredictable cargo packaging [88]. This nonselective nature of packaging is often termed passive packaging or loading [88]. The SRC‐derived N‐myristoylation peptide supported efficient packaging of EGFP‐NLuc into EVs, likely due to its strong membrane affinity and potentially aided by the small size of SRC‐EGFP‐NLuc fusion protein (approximately half the molecular weight of the transmembrane VAD‐EGFP‐NLuc fusion proteins). However, myristoylation of the EGFP‐NLuc fusion protein mediated by CHMP6 also has a low molecular weight, but it resulted in suboptimal EV packaging. The basis for this discrepancy is not immediately clear, as prior reports described efficient EV loading with CHMP6‐derived motifs, whereas our experiments did not reproduce that outcome. Post‐translational modifications such as myristoylation mediated by the SRC octapeptide can promote efficient luminal cargo loading, but they do not enable surface display of functional ligands [41]. In this context, although both SRC and PTGFRN supported robust packaging, PTGFRN was the most versatile choice because its transmembrane domains permit dual functionality for efficient intraluminal cargo loading together with surface ligand expression.

Although transient engineering of HEK293T cells enabled efficient packaging of EVs with the exogenous molecular cargo, the small experimental scale hindered the functional testing of these eEVs. To overcome this, we evaluated multiple biopharmaceutical cell lines for scalable eEV production with the specific aim to assess the stability of their native cargo composition post‐engineering. A cumulative loss of approximately 75% of particles was observed during EV isolation from large volumes of culture medium, with ultrafiltration‐based volume concentration alone accounting for more than 50% of the particle loss. This substantial loss is likely attributable to membrane fouling, which can restrict flow, reduce throughput, and potentially damage vesicles during processing [89]. This observation emphasizes the need for techniques that can minimize particle loss. In contrast to dead‐end filtration methods such as ultrafiltration, tangential flow filtration (TFF), where fluid flows parallel to the membrane surface, can enable concentrations of large volumes while reducing particle loss [89]. However, using TFF alone for EV isolation from large‐scale cultures is not ideal, as co‐isolated soluble proteins can confound EV quantification and potentially compromise overall in vivo safety [90, 91, 92]. Large‐scale manufacturing, therefore, requires purification strategies that balance yield and purity. Consequently, workflows combining TFF with SEC can be adopted for scalable EV isolation, as they enable the processing of large volumes while reducing contamination from soluble proteins and other extracellular components [93].

Notably, ExpiCHO and Expi293 produced high EV yields, whereas Lonza CHO, despite its reputation for stable yields, produced the lowest EV concentration [76, 77]. However, the compliance of Lonza CHO cells with regulatory manufacturing, along with their suitability for cGMP production, could be advantageous in certain circumstances, particularly for the development of clinical EV manufacturing [94, 95]. Proteomic profiling of ExpiCHO‐derived eEVs confirmed high post‐engineering fidelity, showing minimal deviation from WT cargo profiles. This demonstrates that ExpiCHO cells can maintain robust and stable EV biogenesis following genetic manipulation, making them ideal for large‐scale EV production.

Transient expression often suffers from poor reproducibility due to inconsistent transfection efficiency and plasmid uptake. These limitations are particularly detrimental when relying on endogenous sorting machinery for selective EV packaging. While transient transfections can be quick and robust for generating transgene expression at the cellular level, this approach is often confounded by batch‐to‐batch variations and faces challenges in upscaling for clinical manufacturing [76]. A key challenge in the large‐scale production of EVs and EV‐based therapeutics is cargo heterogeneity arising from the stochastic nature of vesicle biogenesis, which can compromise product consistency and therapeutic reproducibility. While workflows combining TFF and SEC are increasingly adopted to balance yield and purity during large‐scale EV productions [93], standardized quality control approaches such as particle‐to‐protein ratio, EV marker profiling, and contaminant assessment will be essential for ensuring batch consistency and safety during clinical translation of EV‐based therapeutics. Therefore, to circumvent the batch variations in scalable EV production, we developed stable expression ExpiCHO cell pools, using EGFP fluorescence as a threshold for selection by flow sorting. While monoclonal lines offer greater clonal uniformity [96], validated polyclonal pools enabled faster optimization. We developed two stable ExpiCHO cell lines expressing EGFP‐NLuc fusion protein bound to the C‐terminus of PTGFRN. One of the two lines was engineered to express EB2 ligand fused to the extraluminal domain on the N‐terminus of PTGFRN, ensuring its expression on the surface of EVs (EB2^+ve^). A corresponding cell line expressing PTGFRN‐EGFP‐NLuc without the EB2 ligand EB2^−ve^, served as a control.

In the present study, although ExpiCHO cells provide a robust and scalable platform to produce eEVs, their xenogeneic origin represents a major bottleneck for direct clinical translation. EVs derived from non‐human sources inherently carry species‐specific molecular cargo, which may elicit immune responses and limit their suitability for systemic administration in humans [31]. In contrast to conventional biologics, which involve the purification of defined molecules, such as monoclonal antibodies produced in pharmaceutically relevant cell lines, EV‐based delivery systems retain the intrinsic molecular cargo of parental cells, necessitating consideration of immunogenicity and regulatory compatibility. Accordingly, ExpiCHO cells were employed in the present study as a platform development model for scalable biomanufacturing of engineered EVs, and these workflows may be subsequently adapted to allogeneic or autologous cell sources to facilitate future clinical translation and evaluation

EVs primarily mediate cargo delivery via surface receptor interactions, often involving integrins, tetraspanins, or Ig‐superfamily proteins [31]. However, the native EV proteome provides limited targeting specificity, emphasizing the need for rational surface engineering [31, 42, 49, 97]. In this work, EVs were modified to overexpress the extracellular domain of Ephrin‐B2 (EB2) for targeting ovarian tumors expressing EphB4 receptors. For targeting eEVs to ovarian tumors, we used the specific topological domain from the Ephrin‐B2 protein known to specifically bind with the Ephrin‐B4 receptor [15]. Endogenously, EB2 is expressed as a transmembrane protein, and structural studies performed to analyze the thermodynamics of its interaction with the EphB4 receptor have identified that the extracellular domain of EB2 is sufficient for its high‐affinity binding with its receptor [15]. In theory, by only expressing the extracellular domain of EB2 on the surface of eEVs, we trimmed its size by almost half without affecting its binding affinity [15]. Previous studies have also leveraged EphB4 for selective nanocarrier delivery, most commonly by chemically functionalizing synthetic gold‐based nanoparticles with EphB4 binding TNYL/TNYL‐RAW peptide [98, 99, 100, 101]. These studies demonstrated enhanced uptake of TYNL functionalized hollow gold nanospheres, as well as polymeric micelles incorporating hollow gold nanospheres and chemotherapeutics in EphB4 positive tumors. This improved photothermal and photochemotherapeutic efficacy in vivo, indicating EphB4 can be leveraged as an effective delivery receptor [98, 99, 100, 101]. In contrast, our study uses biologically derived vesicles and presents the extracellular domain of the native EphB4 ligand, EB2, rather than a synthetic peptide. This design is intended to retain high‐affinity receptor engagement while potentially reducing the signaling complexity associated with full‐length ligand presentation on the donor membrane. Future studies will be required to directly compare the binding affinity of EB2‐displaying EVs with that of synthetic EphB4‐binding peptides such as TYNL/TYNL‐RAW, as well as to determine how receptor engagement by these ligands influences downstream EphB4 signaling in tumor cells.

Despite differences in intracellular EGFP and NLuc expression, proteomic analysis confirmed equimolar packaging of EGFP‐NLuc in EVs from both EB2^+ve^ and EB2^−ve^ lines, highlighting PTGFRN‐mediated selective enrichment. Notably, two distinct EGFP‐reactive bands were consistently observed on immunoblots of protein contents from both cell lysates and eEVs, but we identified that their expression was limited to EB2^+ve^ cells and the corresponding EVs. Additionally, this was not observed for EB2^−ve^ cells and EVs expressing PTGFRN, or for cells and EVs with other transmembrane VADs such as CD63 or LAMP2B. The presence of an additional band in both cells and EVs suggests that it arises during intracellular processing of EB2 bearing PTGFRN fusion protein and is subsequently carried into EVs. Based on prior evidence that extracellular PTGFRN sequence can be proteolytically labile, including sensitivity mitigated by truncation of the N‐terminal extracellular domain, this species is most consistent with construct‐specific proteolytic processing rather than a general feature of PTGFRN‐mediated packaging [28]. This possibility is complicated by the findings of Dooley et al. [28], who demonstrated that strategic removal of N‐terminal amino acids from the PTGFRN extraluminal domain rendered it resistant to ADAM10, a metalloprotease, thereby enabling efficient and stable enrichment of a broad range of proteins on the EV surface [28]. The specific mechanism and the degree of this potential EB2‐related proteolytic cleavage were not further explored here, but we plan to investigate this in the future; deciphering the stability and processing of ligands expressed on the surface of EVs was beyond the scope of the present study.

In parallel, comparative enrichment analysis between EB2^+ve^ and EB2^−ve^ EVs suggested that displaying EB2 ligand on the EV surface selectively remodels EV proteomics rather than inducing broad immune‐activating reprogramming. EB2^+ve^ EVs were preferentially associated with protein trafficking, actin‐based vesicle transport, and membrane localization pathways, consistent with modulation of EV surface and interaction‐based features. In contrast, EB2^−ve^ EVs showed greater enrichment for chromatin‐associated, RNA processing, and immune response pathways. While these data do not directly establish biocompatibility or immunogenicity, they argue against the immune‐activating effects of EB2‐expressing EVs. Instead, these findings suggest that ligand display enhances EV‐associated trafficking and membrane interaction pathways. Direct in vivo safety and immunocompatibility studies will be required to confirm these effects. To evaluate in vivo targeting, we employed patient‐derived xenograft (PDX) models recapitulating the heterogeneity of high‐grade serous ovarian cancers [102, 103]. IHC confirmed variable EphB4 expression and subcellular localization. Surface‐specific expression is critical for EV docking and internalization, which can be confounded if the targeted antigen is sequestered cytosolically [31, 37, 49]. Hence, we selected a PDX model (PDX3) with surface‐specific expression of EphB4 for analyzing systemic administration of eEVs with and without the EB2 ligand.

In vivo imaging revealed selective accumulation of EB2^+ve^ eEVs in EphB4‐expressing tumors. Despite confirming EphB4 expression in the lungs, we observed minimal signal in thoracic regions post‐injection, likely due to limited FFz substrate diffusion across the diaphragm or photon attenuation in deep tissues [80, 104]. Ex vivo imaging confirmed lung accumulation, supporting this hypothesis. Radiance normalized to tissue weight further revealed the highest signal density in tumors, confirming the targeting specificity of EB2^+ve^ eEVs. This observation further highlights that EVs exhibit organ‐selective biodistribution, which is often strongly influenced by factors such as administration routes and the cellular source of the vesicles [105, 106]. Across multiple in vivo models, the lungs have frequently been reported as a major site of EV accumulation, particularly following nebulized or intratracheal administration [105]. However, the lungs are not always the dominant organ following IV delivery. For instance, after IV administration of EVs derived from umbilical cord fetal mesenchymal stem cells in rats, the highest accumulation was observed in metabolic organs, particularly the spleen [107]. Collectively, these findings suggest that the route of administration, EV source, and surface functionalization are key determinants of EV biodistribution in vivo. Importantly, across early human trials and large animal models, engineered EVs have often exhibited favorable short‐term safety and low detectable immunogenicity, while exerting targeted immunomodulation [108, 109]. However, robust data on long‐term, repeat‐dose immunogenicity, especially for allogeneic and xenogeneic EVs, remain limited and would be a key area for future investigations.

Altogether, our findings present a scalable and customizable EV engineering platform capable of delivering exogenous cargo to a specific tumor target. This strategy holds promise for enhancing therapeutic precision by combining luminal cargo enrichment and surface ligand display. Such a system can be adapted to carry diverse therapeutic payloads, including proteins such as genome editors or immune modulators, and nucleic acids such as mRNA or siRNA. The versatility to accommodate these different cargo molecules broadens its potential utility in both oncology and other disease settings. Importantly, EphB4 expression is elevated in multiple malignancies, including prostate, colorectal, bladder, and breast cancer [110, 111, 112, 113]. This pan‐cancer relevance further underscores the translational potential of our approach. The modularity of the PTGFRN platform and its compatibility with pharmaceutically relevant cell lines help position engineered EVs as a next‐generation delivery vehicle for precision oncology and beyond.

## 4 Conclusions

Our study presents a robust strategy for the development of an EV engineering platform that is capable not only of achieving efficient intraluminal cargo packaging but also of enhancing targeted delivery toward ovarian tumors. By utilizing PTGFRN as a transmembrane scaffold for endogenous sorting, we demonstrated efficient equimolar packaging of EGFP‐NLuc bioluminescent cargo within the EV lumen. Nonetheless, by leveraging its extraluminal domain, we simultaneously expressed a functional ligand, Ephrin‐B2, on the surface of EVs to enhance their targeting potential. The use of biopharmaceutically relevant ExpiCHO cells to develop stable lines acted as a miniaturized bioreactor to produce engineered EVs, ensuring scalability and high reproducibility. The in vivo targeting of EB2^+ve^ eEVs, demonstrated using ovarian cancer PDXs, highlighted their tumor targeting properties.

Collectively, our study presents a scalable translational platform to produce engineered EVs that can be adapted for the targeted delivery of various therapeutic cargos in the realm of cancer treatment. Future studies aimed at further optimizing the stability of surface ligands and loading of therapeutic cargo in autologous EVs will be a crucial step toward unlocking the full potential of engineered EV delivery systems at the clinical level.

## 5 Materials and Methods

### 5.1 Fusion Protein Design and Plasmid Construction

All transgenes and fusion proteins (*n* = 8, Table 1) were designed in silico using the cloud‐based platform Benchling (Benchling, USA). For *EB2‐PTGFRN‐EGFP‐NLuc* (Plasmid‐1), *EB2‐CD63‐EGFP‐NLuc* (Plasmid‐2), and *EB2‐LAMP2B‐EGFP‐NLuc* (Plasmid‐3), *EB2‐PTGFRN‐EGFP* (Plasmid‐7), a ∼25 kDa extracellular domain of Ephrin‐B2, which selectively binds the Ephrin‐B4 receptor, was fused to N‐terminal extraluminal domains of the respective transmembrane vesicle anchoring domains (VADs) [15]. In CD63‐VAD and LAMP2B‐VAD constructs, the EB2 domain was inserted between two extraluminal loops of the scaffold proteins as described previously [36, 38, 42]. For PTGFRN‐VAD, we engineered the truncated variant [28] to display the EB2 ligand on the EV surface while simultaneously packaging bioluminescent protein cargo within the EV lumen. The truncated variant ∆688 was derived from *Cricetulus griseus* (Chinese Hamster) rather than *Homo sapiens* to prevent protein misfolding and associated packaging discrepancies associated with CHO producer cells. Cargo reporter protein (EGFP‐NLuc) was fused to the C‐terminus of each VAD, with orientation and linker design adopted from Schaub et al. [114] All other domains were connected using single or tandem flexible linkers, unless otherwise specified. Designs for each VADs followed established frameworks reported in prior studies [28, 36, 38, 40, 41, 42]. Translated sequences for all the fusion proteins expressed by the plasmids used in this study are added to Table S1.

The transgenes were cloned into the mammalian expression vector pCMV‐3Tag‐1A between the NotI and ApaI restriction sites using Gibson assembly (NEBuilder HiFi DNA Assembly reagents, New England Biolabs, USA). The DNA sequence for Plasmid‐3 was adopted from our previous study [42], whereas all other constructs were generated from double‐stranded DNA sequences (gBlocks) synthesized by Integrated DNA Technologies (IDT, USA). The constructed plasmids were transformed into 5‐alpha competent E. coli (DH5α, New England Biolabs, USA), and 100 mL midiprep cultures were expanded. The plasmid DNA was extracted using the NucleoBond Xtra endotoxin‐free Midi kit (Macherey Nagel, Germany). All plasmid sequences were confirmed by Sanger sequencing performed at the Australian Genome Research Facility (AGRF, Brisbane).

### 5.2 Cell Culture

Human embryonic kidney (HEK) 293T cells were cultured at 37°C with 5% CO_2_ and 8% O_2_ in Dulbecco's Modified Eagle Medium (Gibco, USA) supplemented with 1% 200 mm GlutaMAX (Gibco, USA), 10% Fetal Bovine Serum (FBS) (Gibco, USA), and 1% Antibiotic‐Antimycotic (Gibco, USA). HEK293T cells were seeded at a density of 3.5 × 10^6^ in T175 flasks, with four replicates for each expression plasmid to be transfected. Once flasks reached ∼60% confluence, at ∼24 h after seeding, ∼50 µg of endotoxin‐free midi‐prep plasmid DNA was transfected into each flask using Lipofectamine 3000 (Invitrogen, USA) according to the manufacturer's protocol (at a 1:1 ratio of DNA:Lipofectamine). Twenty‐four hours post‐transfection, cells were washed and cultured in nutrient‐enriched serum‐free (OptiMEM) medium for 48 h to enable EV secretion into the media. Prior to harvesting conditioned culture medium (CCM), EGFP fluorescence was measured in live cells using Incucyte S3 Live Cell Analysis System (Essen BioScience, Michigan, USA).

For the scalable production of eEVs carrying exogenous cargo, we used the following bio‐pharmaceutically relevant cell lines – ExpiCHO, CHO‐S, FLP‐IN‐CHO, and Expi293 (all sourced from Gibco, USA), and Lonza‐CHO (CHOK1 GS‐KO; sourced from Lonza, Switzerland). Suspension cultures were maintained in serum‐free, chemically defined media as follows: ExpiCHO, ExpiCHO Expression Medium (Gibco, USA); Expi293, Expi293 Expression Medium (Gibco, USA); LonzaCHO, CD CHO supplemented with 6 mm L‐Glutamine (Gibco, USA); CHO‐S and FLP‐In CHO, CD‐CHO (Gibco, USA). All CHO cell lines (ExpiCHO, CHO‐S, and LonzaCHO) were transfected in 30 mL of ExpiFectamine CHO Reagent (Gibco, USA) according to the manufacturer's instructions. HEK cell line (Expi293) was transfected in 25 mL of ExpiFectamine 293 Reagent (Gibco, USA) according to the manufacturer's instructions. Suspension cultures were grown at 37°C, 7.5% CO_2_, and 81% humidity, with shaking at 125–135 rpm. Cells were passaged every 3–4 days to maintain a density between 0.3 × 10^6^ and 6 × 10^6^ cells/mL. For subculture, cells were diluted to 0.3–0.5 × 10^6^ cells/mL with fresh pre‐warmed medium. Cell density and viability were routinely monitored using trypan blue exclusion.

### 5.3 Generation of Stable Pools of ExpiCHO Cells

We generated stable cell pools of two separate ExpiCHO lines expressing EGFP‐NLuc fusion protein tethered to truncated PTGFRN with randomly integrated copies of linearized plasmid DNA, where one pool expressed EB2‐PTGFRN‐EGFP‐NLuc (EB2^+ve^, Ephrin‐B2 ligand tethered to N‐terminus of PTGFRN) and a second pool expressed PTGFRN‐EGFP‐NLuc (EB2^−ve^, lacking the ligand). The cells were cultured in ExpiCHO Expression Medium and maintained at 1 × 10^6^ cells/mL in 125‐mL shake flasks on a shaking platform at 37°C, 7.5% CO_2_, and 81% humidity. For each gene of interest, plasmid DNA was linearized using DraIII restriction enzyme, and independent transfections were performed.

On the day of transfection, 25 µg of linearized EB2‐PTGFRN‐EGFP‐NLuc or PTGFRN‐EGFP‐NLuc plasmid DNA was diluted in 2 mL cold OptiPRO SFM, and separately, 40 µL of ExpiFectamine CHO Reagent was diluted in 2 mL cold OptiPRO SFM. The diluted ExpiFectamine was added to the DNA solution, gently mixed, and incubated for 1–5 min at room temperature. The resulting complexes were slowly added to the cells.

Cells were cultured post‐transfection under standard conditions. Forty‐eight hours post‐transfection, cell viability and titre were assessed, and selection pressure was added 200 µg/mL Geneticin (G418) (Invitrogen, USA). After cells had recovered, approximately 2 × 10^5^ GFP‐positive ExpiCHO‐S cells were sorted into 1 mL of ExpiCHO Expression Medium supplemented with 200 µg/mL Geneticin (G418) (Invitrogen, USA) using a FACSAria III cell sorter (BD Biosciences, USA). Sorted cells were transferred to T25 flasks containing 3 mL of fresh medium and incubated under static conditions at 37°C in a humidified atmosphere with 7.5% CO_2_ for 5–7 days. Cells were then scaled up into 125 mL shake flasks and maintained on an orbital shaker at 130 rpm, 37°C, 7.5% CO_2_, and 81% humidity. Cells were subsequently maintained by passaging every 3–4 days at 0.3 × 10^6^ cells/mL. Once cultures reached a viability of >90% and density >1 × 10^6^ cells/mL, cell banks were established. The timeline to achieve stable pools through selection and sorting typically ranged from 6 to 8 weeks.

### 5.4 Confocal Imaging

The expression of EGFP in the stable pools was validated using confocal microscopy. 10 µL of cell aliquot was pipetted into disposable Countess chamber slides (Invitrogen, USA) and the imaging was performed using a Zeiss LSM 710 confocal microscope (ZEISS, Germany). The acquisition was performed on the Zen Black software (ZEISS, Germany), by using smart setup to autoload preset spectral curve for EGFP fluorescent protein with configuration set to smartest signal, and the objective was set to auto‐focus. The gain for the laser (488 nm) was adjusted to 850–900 arbitrary units for capturing EGFP‐positive cells, while the gain for the transmission photomultiplier tube was adjusted to 200–300 arbitrary units for capturing the brightfield images. The raw files were saved as. czi and were processed using ImageJ software (NIH, USA).

### 5.5 EV Isolation

For adherent HEK293T cells, immediately after Incucyte analysis (24 h post‐transfection), the cells were washed twice with DPBS (Gibco, USA) and then supplemented with reduced serum OptiMEM medium (Gibco, USA) containing 1000 U/mL antibiotic‐antimycotic (Gibco, USA). After 48 h of further culturing, the cell‐conditioned media (CCM) were harvested from the flasks and centrifuged at 500 g for 5 min, 2000 g for 20 min, and 10 000 g for 40 min. The supernatant was collected and stored at −80°C until EV isolation was performed.

EVs were isolated from the differentially centrifuged CCM by ultracentrifugation at 100 000 g for 2 h. The pellet was washed and resuspended in 200 µL of DPBS. The isolated EVs were transferred to 1.5 mL Protein LoBind tubes (Eppendorf, USA) and stored at −800C until downstream characterization.

Cell‐conditioned media (CCM) from the cultures of five suspension cultures were centrifuged at 4000 × g for 20 min at 4°C. The supernatant was filtered through a 0.45 µm filter (Millipore, Massachusetts, USA) and concentrated using Amicon 100 kDa MWCO centrifugal filters (Merck Millipore). The EVs were isolated from the concentrated media using size exclusion chromatography (SEC) with qEV columns and an automatic fraction collector (Izon Science, Christchurch, New Zealand) following the manufacturer's instructions. The isolated EVs were transferred to 1.5 mL Protein LoBind tubes (Eppendorf, USA) and stored at −80°C until downstream characterization.

### 5.6 EV Characterization

Isolated EVs were characterized on the basis of particle size, concentration, morphology, and associated protein content in accordance with the 2023 guideline criteria for minimal information for the studies of extracellular vesicles (MISEV) [30].

### 5.7 Nanoparticle Tracking Analysis

The size and concentration of the EVs were characterized using the NS300 – Nanoparticle Tracking Analysis (NTA) instrument (Malvern Panalytical, UK). The instrument's performance was validated by using NIST traceable size standards 100 nm (Fisher Scientific, MA, USA). The EV samples were diluted in the DPBS to a final volume of 1 mL to an ideal concentration of 30–100 particles per frame. For each measurement, five 30‐s videos were recorded with the camera level set at 13 and syringe flow rate set at 50 arbitrary units. The captured videos were analyzed using NTA 3.4.4 (Malvern Panalytical, UK) software with the detection threshold set at 5.

### 5.8 Cryogenic Transmission Electron Microscopy

The morphology of the isolated EVs was observed using cryogenic transmission electron microscopy (Cryo‐TEM) as described previously in the work done by Sharifpour et al. [115] Samples were prepared using the Leica EM GP2 robotic vitrification system (Leica, Germany), under controlled conditions (temperature, 22°C; relative humidity, 95%). A 3 µL dispersion of EVs was applied onto a carbon‐coated perforated formvar film supported on a 200‐mesh copper TEM grid. The excess solution was blotted for a period of 3–3.5 s and plunged into liquid ethane near its freezing point (−183°C), and the grids were then stored in liquid nitrogen until imaging. Images were captured using the Jeol Cryo ARM 200 (JEM‐Z200FSC) transmission electron microscope (TEM; Jeol, Germany) in a frozen hydrated state at −176°C. Images were captured with zero energy loss at an acceleration voltage of 200 kV and a filter setting of 20 eV. To ensure minimal exposure, images were recorded under low‐dose conditions using the SerialEM software and a Gatan K2 direct detector camera.

### 5.9 Immunoblotting

Protein concentrations of samples lysed using in‐house radioimmunoprecipitation assay (RIPA) buffer were determined by bicinchoninic acid (BCA) protein assay (Thermo Fisher Scientific, MA, USA) performed according to the manufacturer's instructions using bovine serum albumin as the protein standard.

Candidate proteins associated with isolated EVs, including CD9, Flotillin‐1, TSG101, and Alix, specified in the MISEV2023 guidelines, were characterized by immunoblotting. For each EV sample, 10 µg was size‐separated by Sodium Dodecyl Sulfate Polyacrylamide Gel Electrophoresis (SDS‐PAGE) using a Bolt 4%–12% gel and system (Invitrogen, USA). Samples were resolved for 30 min at 200 V, at which point the proteins were transferred from the gel onto an activated polyvinylidene difluoride (PVDF) (Merck, USA) membrane using a Towbin buffer at 200 mA for 90 min. The membrane was blocked for 1 h at room temperature, then incubated with primary antibody overnight at 4°C (primary antibody information is included in the Table S2). The membrane was then washed with Tris‐buffered saline with Tween 20 (TBS‐T) buffer and incubated with host‐specific fluorescently tagged secondary antibody (for Rabbit‐specific primary: IRDye 800CW Goat anti‐Rabbit IgG Secondary Antibody (925‐32211) and for Mouse‐specific primary: IRDye 680RD Goat anti‐Mouse IgG Secondary Antibody (926‐68070)). The membrane was washed using TBS‐T, and protein bands were visualized using fluorescence detection on a ChemiDoc MP imaging system (Bio‐Rad, USA).

### 5.10 Nanoluciferase Assay to Measure Bioluminescence from the Isolated EVs

All of the transfected plasmids encoded fusion proteins with a C‐terminal Nanoluciferase domain; when the expressed Nanoluciferase enzyme is activated by interaction with its specific chemical substrate, furimazine, the interaction causes emission of a detectable bioluminescent signal (peak wavelength, 460 nm). The emission signal at 460 nm was measured to determine the efficiency of Nanoluciferase‐tagged protein packaging inside the EVs to ensure that the bioluminescent signal originates from the reporter cargo packaged intraluminally inside the eEVs, protease protection assay was performed as described previously in the work done by Bonsergent et al. [116] The isolated EV samples were incubated with PBS, with or without 0.1% Triton X‐100, and 50 µg/mL Proteinase‐K for 3 h at 37°C. 20 µL of NanoGlo Live Cell Assay Reagent (Promega, USA), containing the Nanoluciferase substrate furimazine, was added to each reaction in opaque 96‐well reaction plates, according to the manufacturer's instructions. Bioluminescence was measured for all wells using the Spark10M multimode microplate reader (Tecan, Switzerland) with the following settings: Attenuation, Auto; Settle Time, 0 ms; Integration Time, 1000 ms.

### 5.11 Nano‐Flow Cytometry

To further analyze reporter cargo packaging inside EVs, EGFP fluorescence from EVs was measured using a CytoFLEX flow cytometer (Beckman Coulter, Pasadena, CA) equipped with 405, 488, and 640 nm lasers. The 405 nm laser was selected for side scatter (V‐SSC) detection, with a manual threshold set at 1800 (height channel) and a V‐SSC signal gain of 100. EVs in DPBS were loaded at a flow rate of 10 µL/min, and data acquisition was performed for 20 s once a stable event rate was reached. Calibration of the sample flow rate was conducted according to the manufacturer's instructions by water weight measurement over 18 min at slow flow. Data were processed using CytExpert 2.0 software (Beckman Coulter, USA), with background subtraction from control samples to calculate events/mL. Standard fluorescent beads (Gigamix, BioCytex) and 100 nm Fluoresbrite YG microspheres (Polysciences, Inc.) were used for instrument calibration and particle sizing. GFP‐positive EV populations were specifically gated and quantified based on the 488 nm fluorescence signal.

### 5.12 Immunohistochemistry

Immunohistochemical analysis was performed using MACH1 Universal HRP‐Polymer Detection Kit (Biocare Medical, Pacheco, CA, USA, Catalog #M1U539 G, L10) on 4 µm formalin‐fixed, paraffin‐embedded tissue whole sections of tumor tissues obtained from patient‐derived xenograft models and control tissues such as liver, kidney, spleen, muscles, heart, and lungs. Standard protocols were followed, using heat‐induced epitope retrieval with citrate buffer (0.01 m, pH 6.0) using a decloaking chamber NxGen (Biocare Medical). The sections blocked with MACH1 Background Sniper (Biocare Medical, Pacheco, CA, USA) and Ephrin‐B4 primary antibody (Invitrogen, 3D7G8, USA) were diluted in the DaVinci Green Diluent at a concentration of 1:50.

### 5.13 Mass Spectrometry Sample Preparation, Data Acquisition, and Analysis

EV samples (20 µg) were mixed with an equal volume of lysis buffer (final SDS concentration: 1%). EV and cell lysates were denatured and reduced by dithiothreitol (DTT) to a final concentration of 20 mm and incubated at 37°C for 1 h. Free thiols were alkylated using iodoacetamide (IAA) to a final concentration of 40 mm in the dark at room temperature for 30 min. For buffer exchange and removal of detergents, 4 × 8 m urea was added to each sample, followed by filtering through 30 kDa molecular weight cut‐off filters. Samples were then washed twice using 8 m urea buffer and twice with 50 mm ammonium bicarbonate (ABC) to remove residual urea. Proteins were digested overnight at 37°C using mass spectrometry‐grade trypsin (ThermoFisher, USA) at an enzyme‐to‐protein ratio of 1:80 (w/w). Digested peptides were eluted using 50 mm ABC and then vacuum‐dried at room temperature.

Peptide samples were analyzed on either a TripleTOF 5600 or ZenoTOF 7600 mass spectrometer (Sciex) for protein identification and quantification. For global quantification, data‐independent acquisition (DIA) was applied, and raw files were analyzed using DIA‐NN (v1.8.2 beta 27) in a library‐free mode against the UniProt *Homo sapiens* and *Cricetulus griseus* (Chinese Hamster) reference database. Precursor ion generation was carried out using default DIA‐NN settings. False discovery rates (FDR) for both peptide and protein identification were controlled at 1%, and cross‐run alignment (match between runs) was enabled to improve quantitative accuracy.

### 5.14 Construction of 3D Chimeras

To generate the 3D structural models of the chimeric receptors, the signal peptides, protein domains, and transmembrane segments were first identified using UniProt reference sequences (The UniProt Consortium, 2023). The extracellular domain of EPHB2 (P52799, residues 28–299) was used as a common backbone, fused to selected domains from PTGFRN (Q9P2B2; extracellular residues 688–832, transmembrane 833–853, intracellular 854–879), CD63 (P08962; residues 1–200 encompassing three transmembrane segments, followed by the EB2 extracellular residues 201–238), and LAMP2B (P13473; first 17 residues after the signal peptide, insertion of the EB2 extracellular domain, and continuation through its transmembrane region plus 11 cytosolic residues). Three models were generated for each chimera after removal of signal peptides, employing Boltz2 for protein folding and dimeric assembly to approximate physiological states [117, 118]. For each chimera, we evaluated 20 diffusion sample models. Functional interaction modeling incorporated the cytoplasmic domain of Ephrin B4 (P54760, residues 16–539), co‐crystallized with Ephrin B2 in PDB entry 2HLE [15]. The final complex was generated as an ensemble with Modeller v10.7 [119] and the visualizations were prepared and refined with ChimeraX [120], with domain origins color‐coded according to sequence provenance.

### 5.15 Ovarian Cancer Patient‐Derived Xenograft (PDX) Mouse Models

All animal experiments were performed in accordance with the institutional (2021/AE000852) and national guidelines for animal care and use. Patient‐derived xenografts of high‐grade serous ovarian tumor models were established as intraperitoneal tumors in female NOD.Cg‐Prkdcscid Il2rgtm1Wjl/SzJ (NSG) mice as described previously in the work done by Harrington et al. [121, 122] Freshly dissociated serous ovarian carcinoma specimens were used with approval of the Mater Health Services Human Research Ethics Committee and with informed consent from participating patients. All experimental procedures complied fully with the ethical guidelines set forth by the institutional research committee and adhered to the standards outlined in the 1964 Helsinki Declaration and its subsequent amendments or equivalent ethical guidelines.

### 5.16 Detection of Biodistribution In Vivo

To establish an ovarian tumor model, freshly dissociated slurry from high‐grade serous ovarian PDX tumors was injected intraperitoneally into immunodeficient mice. Upon tumor development (in approximately 8–12 weeks), mice were randomized into three groups (*n*  =  4 per group), then intravenously injected via the tail vein with 100 µg of EVs as follows: Group 1 (EB2^+^^ve^ EVs), Group 2 (EB2^−ve^ EVs), and Group 3 (wild‐type EVs). Mice were anesthetized using isoflurane, and 5 min post‐EV injection, 20 µg flurofurimazine (FFz) substrate (Promega, USA) was administered intraperitoneally. In vivo imaging of anesthetized mice was performed using an IVIS system (PerkinElmer, USA). Images were captured longitudinally over 30 min following substrate administration across three sequential detection channels: total luminescence (open filter), NLuc luminescence (500 nm), and EGFP fluorescence (520 nm), with the excitation filter blocked throughout acquisition. BRET signals were recorded using the open filter to enable broad‐spectrum photon detection from NLuc and EGFP reporters.

### 5.17 Ex Vivo Imaging for Bioluminescence

At 30 min post intraperitoneal injection, the brain, heart, lungs, liver, spleen, kidneys, ovaries, and tumors were harvested from each animal to assess the extraperitoneal and organ‐specific localization of eEVs. Tissues were immersed in 1/500 flurofurimazine (FFz) substrate (Promega, USA) solution and subjected to ex vivo imaging using the IVIS system. BRET signals were captured using the open emission filter to enable broad‐spectrum detection of photons emitted from both NLuc and EGFP reporters. Acquired images were analyzed by quantifying the maximum radiance within selected regions of interest (ROI) for each organ.

### 5.18 Statistical Analysis

All data were analyzed using GraphPad Prism (Version 9.4.1, San Diego, CA). Statistical significance was assessed using one‐way ANOVA, with multiple comparisons performed using Tukey's test. Data shown are mean values ± SEM with p values ^*^ (*p* ≤ 0.05), ^**^ (*p* ≤ 0.01), ^***^ (*p* ≤ 0.001), ^****^ (*p* ≤ 0.0001) considered statistically significant. For the normalized proteomics data generated from DIA‐NN, a two‐tailed unpaired t‐test was used to determine significant differences (*p*‐value <.05). Plotting of heatmaps, volcano plots, and Venn diagrams was performed using Python scripts.

## Author Contributions

N.G.: Writing (original draft), conceptualization, investigation, writing (review and editing), methodology, data curation, validation, formal analysis, project administration, and visualization. A.L.: Investigation, methodology, data curation, validation, supervision. A.Q.: Writing (original draft), investigation, methodology, conceptualization, writing (review and editing), validation, formal analysis, supervision. M.G.: Writing (original draft), methodology, data curation, validation, writing (review and editing), supervision. d.g.: methodology, data curation, formal analysis, supervision. K.S.‐R.: Resources and project administration. A.S.B.: Writing (original draft), methodology, data curation, formal analysis, software, and visualization. B.L.D.: Methodology, data curation, formal analysis, software, and visualization. R.L.: Resources. N.C.: Resources. R.L.: Methodology, data curation, formal analysis, resources. K.B.: Methodology, data curation, formal analysis, resources, writing (review and editing). A.E.M.R.: Resources, writing (review and editing). K.F.: Resources. B.S.: Methodology, data curation, formal analysis, resources. Y.H. Methodology, data curation, formal analysis, and resources. J.D.H.: Resources, funding acquisition, writing (review and editing). C.S.: Conceptualization, writing (review and editing), resources, funding acquisition, supervision, and project administration.

## Funding

This project was conducted with the support of the Medical Research Future Fund (MRF1199984), National Health and Medical Research Council (NHMRC 1195451), the Donald & Joan Wilson Foundation Ltd. (2020000323), and the Ovarian Cancer Research Foundation (OCRF, 2018001167).

## Conflicts of Interest

The authors declare no conflicts of interest.

## Supporting information




**Supporting File**: advs75415‐sup‐0001‐SuppMat.docx.

## Data Availability

All data needed to evaluate the conclusions in the paper are present in the paper and/or the Supplementary Materials.
