# Supplementary material for: Scalable Engineering of Bio‐Manufactured Extracellular Vesicles for Selective Delivery in Ovarian Cancer Patient‐Derived Models
Source: Adv Sci (Weinh). 2026 Apr 23;13(40):e75415. doi: 10.1002/advs.75415 (PMC13335665; doi:10.1002/advs.75415)
Supplement: Supplementary file 1 — Supporting File: advs75415‐sup‐0001‐SuppMat.docx. [file ADVS-13-e75415-s001.pdf]

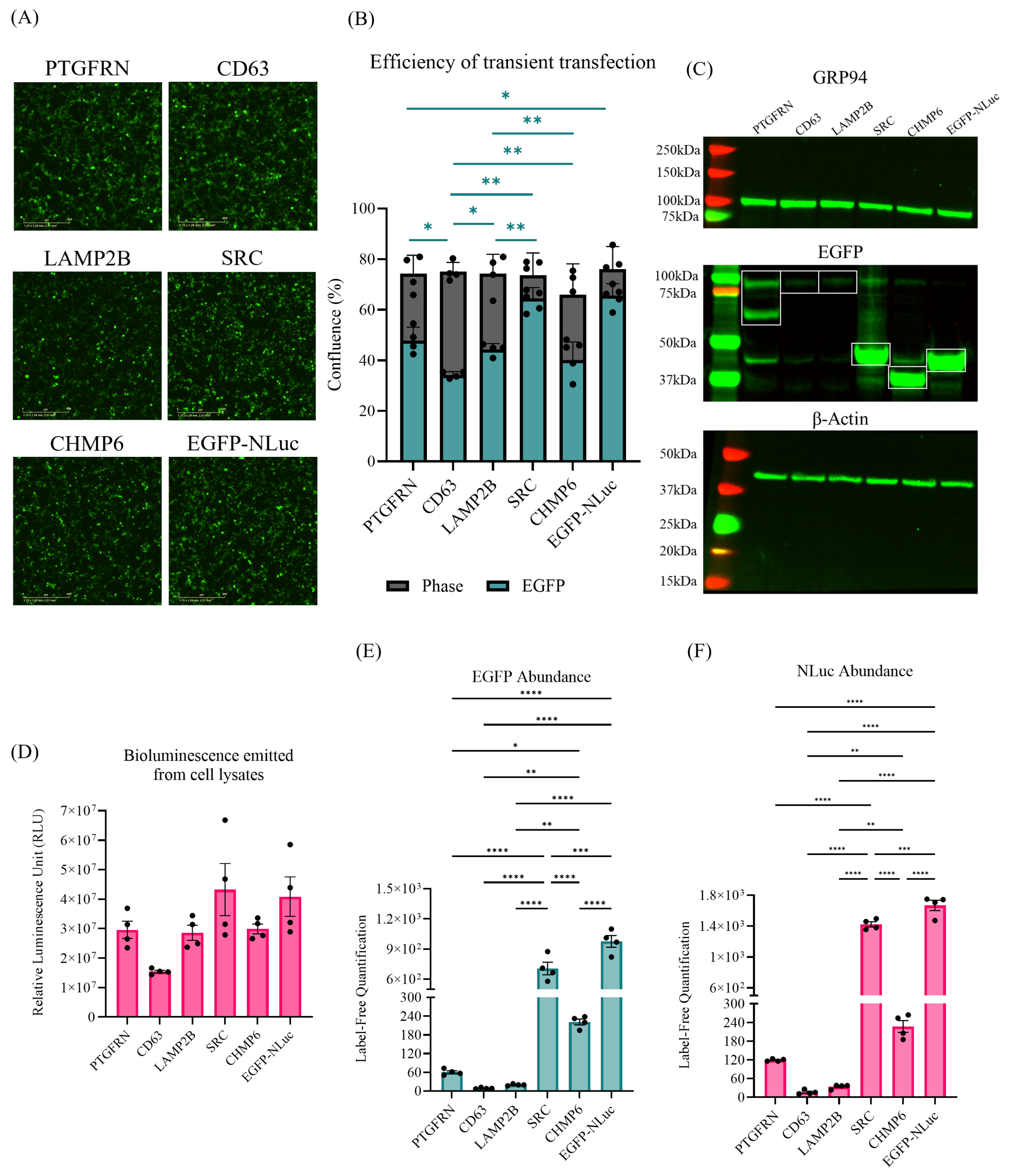

Figure S1: Assessing transfection efficiency and the expression of fusion protein in engineered cells. (A) EGFP expression in transfected HEK293T cells. (B) Transfection efficiency of the VAD-EGFP-NLuc plasmids in the HEK293T cells (percentage of cells that were EGFP-positive). (C) Immunoblot images showing GRP94, EGFP and B-Actin protein expression in cell lysates transfected with the different VAD-EGFP-NLuc plasmids. (D) Nanoluciferase activity in protein extracts from HEK293T cell lysates transfected with the various plasmids. (E-F) Quantitative proteomic analysis of EGFP and NLuc expression in transfected cells. EGFP (E) and NLuc (F) levels in cell lysates were quantified by DIA mass spectrometry using LFQ approach. Statistical significance was assessed using one-way ANOVA with multiple comparison performed using Tukey's tests, with $P < 0.05$ considered significant. * ($P \leq 0.05$), ** ($P \leq 0.01$), *** ($P \leq 0.001$), **** ($P \leq 0.0001$).

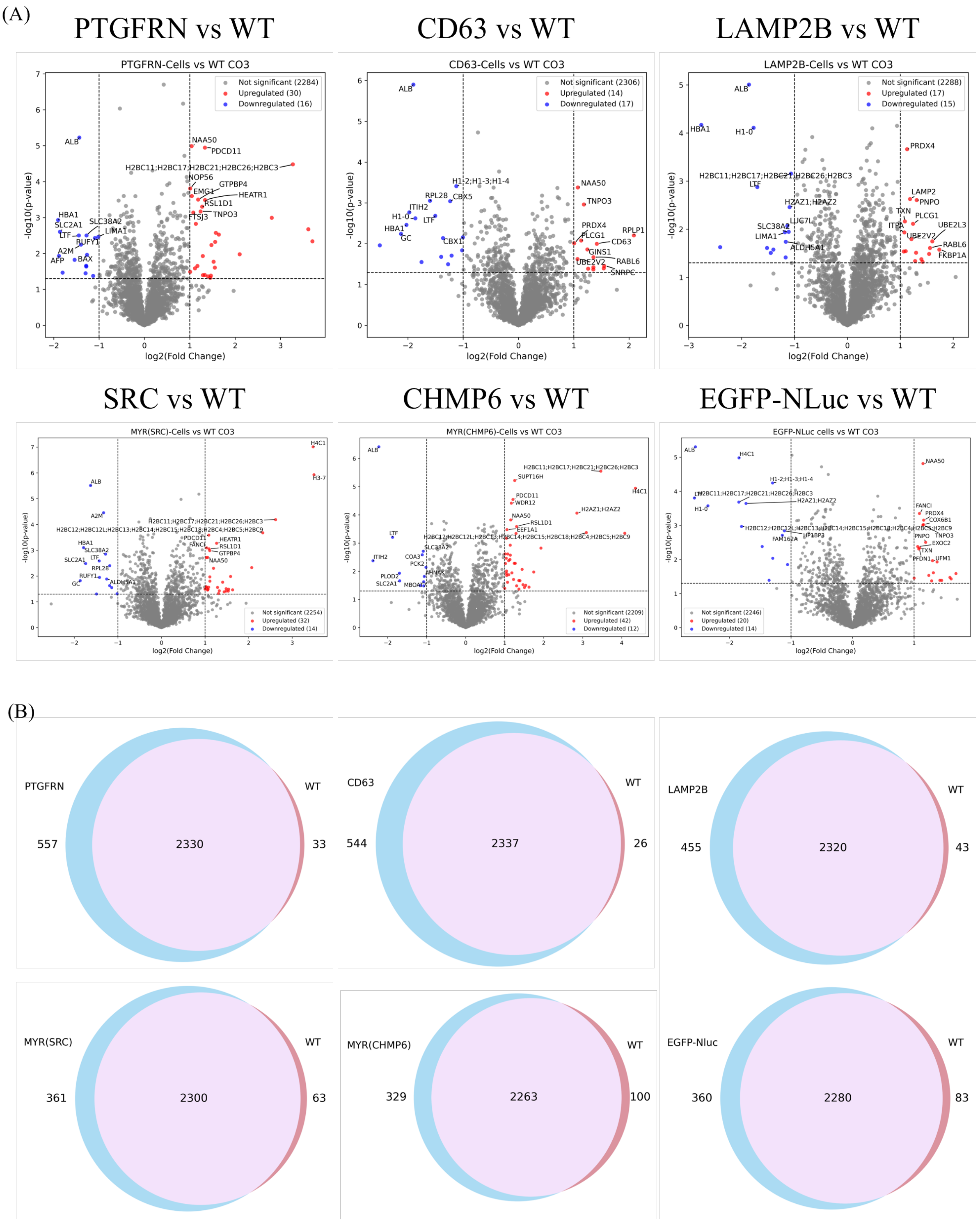

Figure S2: Global proteomic profiling of transfected and wild-type cells. Comparative analyses of proteomic profiles were performed to assess transfection-associated changes in protein abundance. (A) Volcano plots display differential protein expression between transfected and wild-type cells, with thresholds set at log_2 fold change > 1.0 or < –1.0 and $p < 0.05$. (B) Venn diagrams illustrate the distribution of proteins unique to each condition, shared between transfected and wild-type cells.

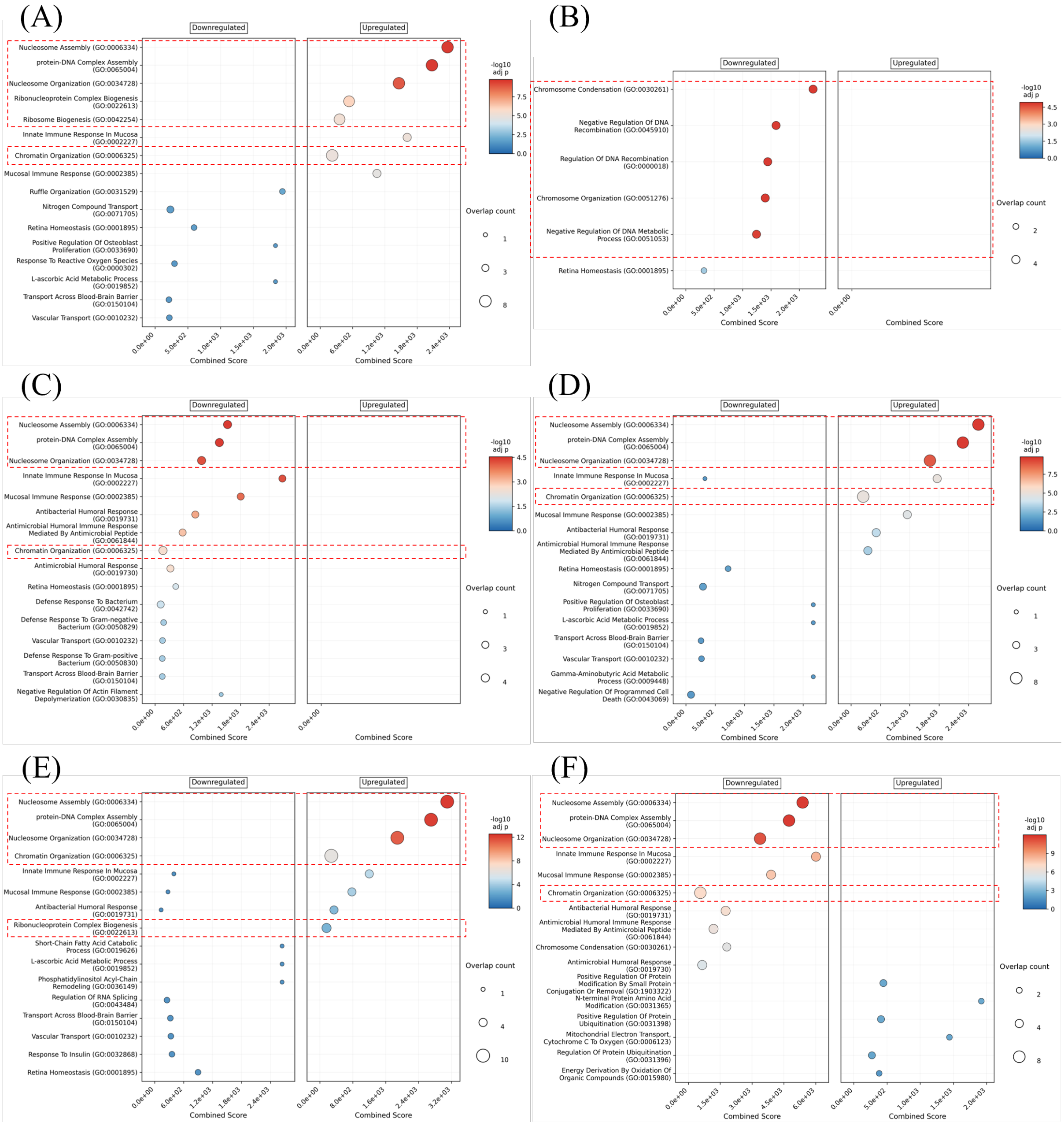

Figure S3: Gene ontology (GO) biological process enrichment of proteins altered by VAD expression in HEK293T cells. (A–F) Dot plots showing significantly enriched GO Biological Process terms derived from proteins classified as downregulated (left) or upregulated (right) relative to WT cells for each construct: (A) PTGFRN, (B) CD63, (C) LAMP2B, (D) MYR(SRC), (E) MYR(CHMP6), and (F) EGFP–NLuc. The x-axis represents the combined enrichment score, which integrates the significance and magnitude of enrichment for each term. Dot color indicates the –log10 adjusted p value (Benjamini–Hochberg corrected; higher values denote greater statistical significance), and dot size reflects the overlap count (number of proteins from the dataset associated with each GO term). Red dashed boxes highlight major functional clusters.

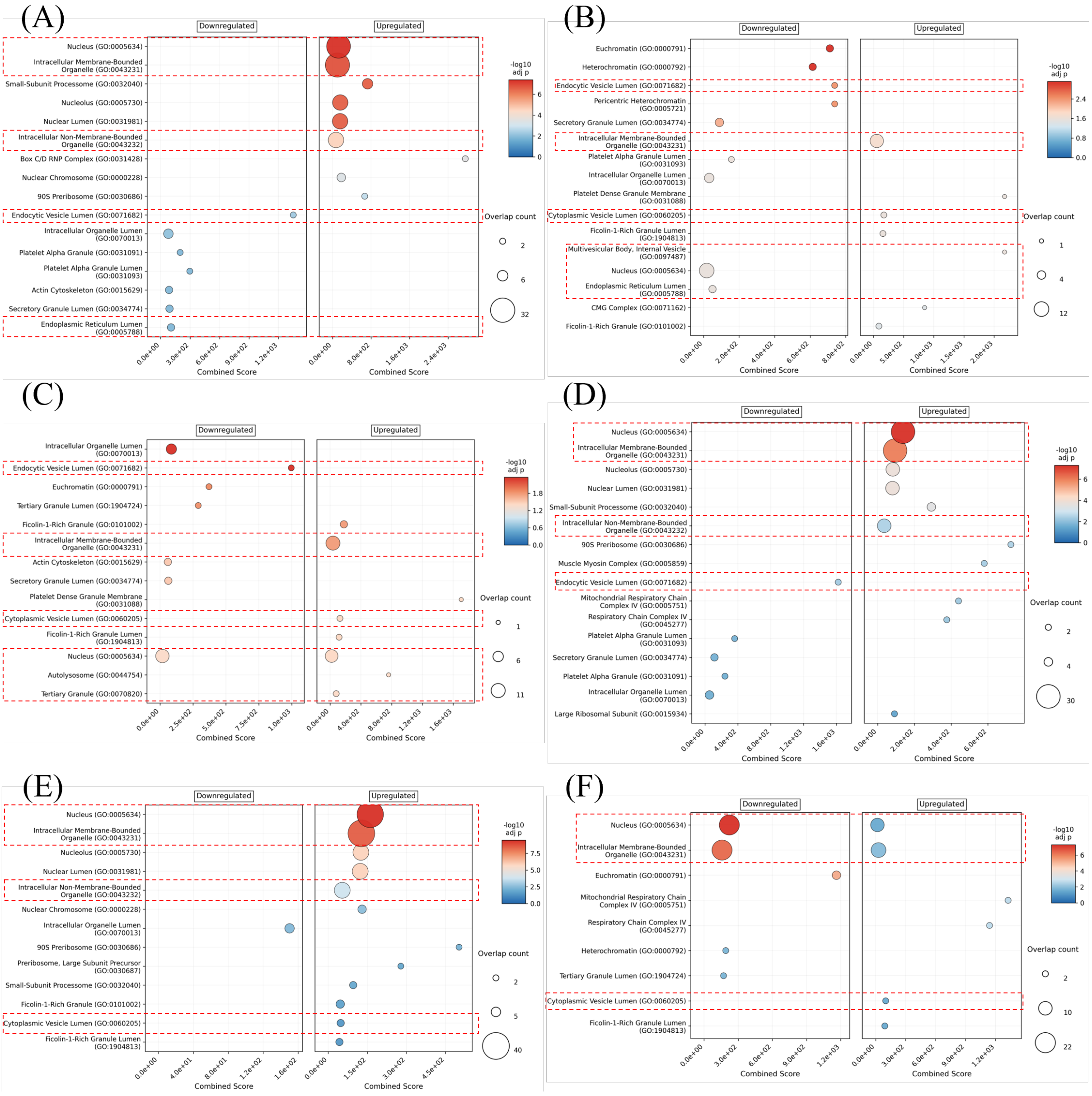

Figure S3: Gene ontology (GO) cellular component enrichment of proteins altered by VAD expression in HEK293T cells. (A–F) Dot plots showing significantly enriched GO Biological Process terms derived from proteins classified as downregulated (left) or upregulated (right) relative to WT cells for each construct: (A) PTGFRN, (B) CD63, (C) LAMP2B, (D) MYR(SRC), (E) MYR(CHMP6), and (F) EGFP–NLuc. The x-axis represents the combined enrichment score, which integrates the significance and magnitude of enrichment for each term. Dot color indicates the –log10 adjusted p value (Benjamini–Hochberg corrected; higher values denote greater statistical significance), and dot size reflects the overlap count (number of proteins from the dataset associated with each GO term). Red dashed boxes highlight key subcellular compartments.

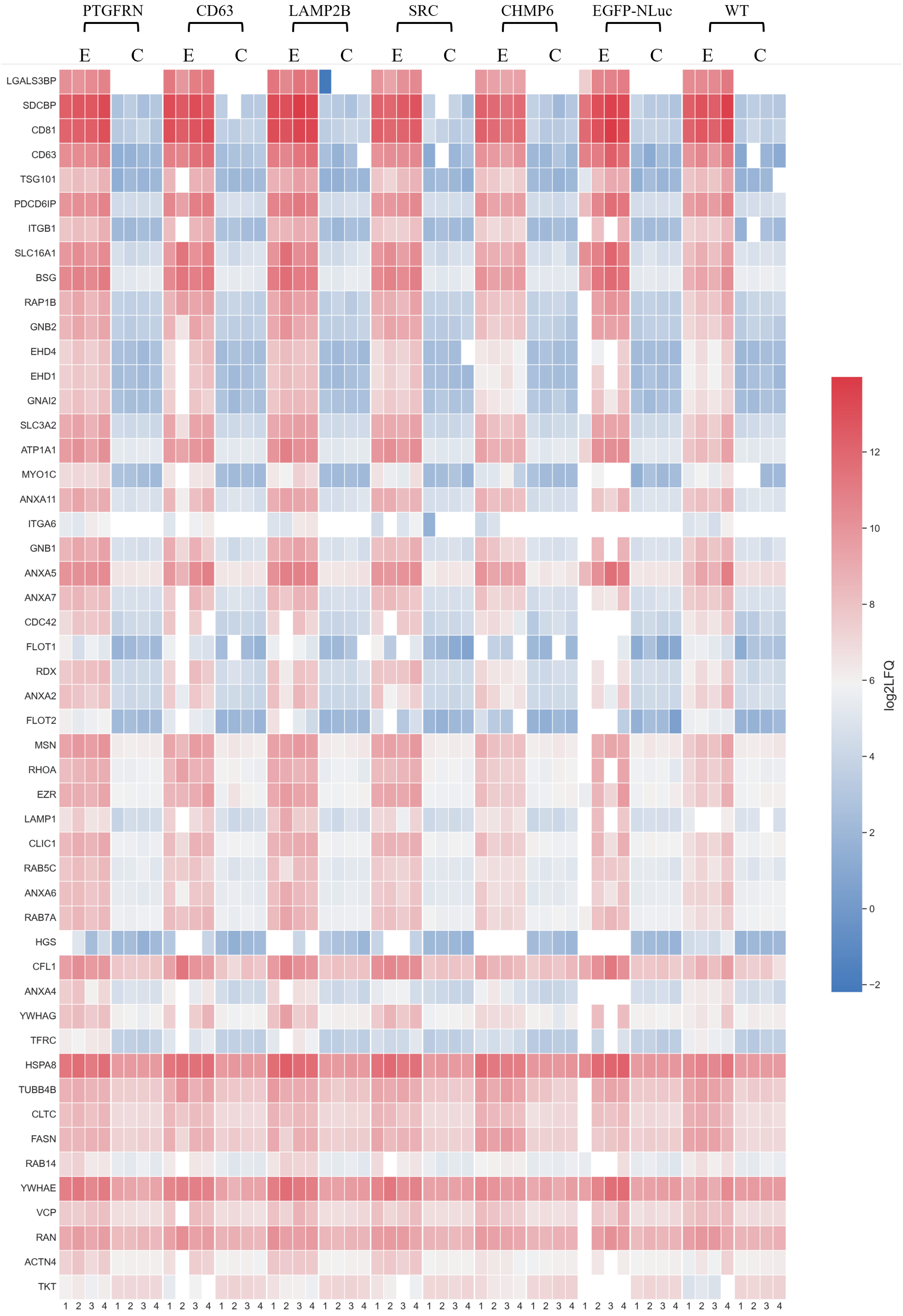
PTGFRN
CD63
LAMP2B
SRC
CHMP6
EGFP-NLuc
WT
E
C
LGALS3BP
SDCBP
CD81
CD63
TSG101
PDCD6IP
ITGB1
SLC16A1
BSG
RAP1B
GNB2
EHD4
EHD1
GNAI2
SLC3A2
ATP1A1
MYO1C
ANXA11
ITGA6
GNB1
ANXA5
ANXA7
CDC42
FLOT1
RDX
ANXA2
FLOT2
MSN
RHOA
EZR
LAMP1
CLIC1
RAB5C
ANXA6
RAB7A
HGS
CFL1
ANXA4
YWHAG
TFRC
HSPA8
TUBB4B
CLTC
FASN
RAB14
YWHAE
VCP
RAN
ACTN4
TKT
1 2 3 4
log2LFQ
12
10
8
6
4
2
0
−2

Figure S5: Heatmap for the expression levels of EV specific markers in the EVs and their corresponding producer cells. Expression of majority of EV-specific canonical markers was elevated in EVs when compared to their corresponding producer cells. The expression levels for all the identified markers were comparable in engineered and WT EVs.

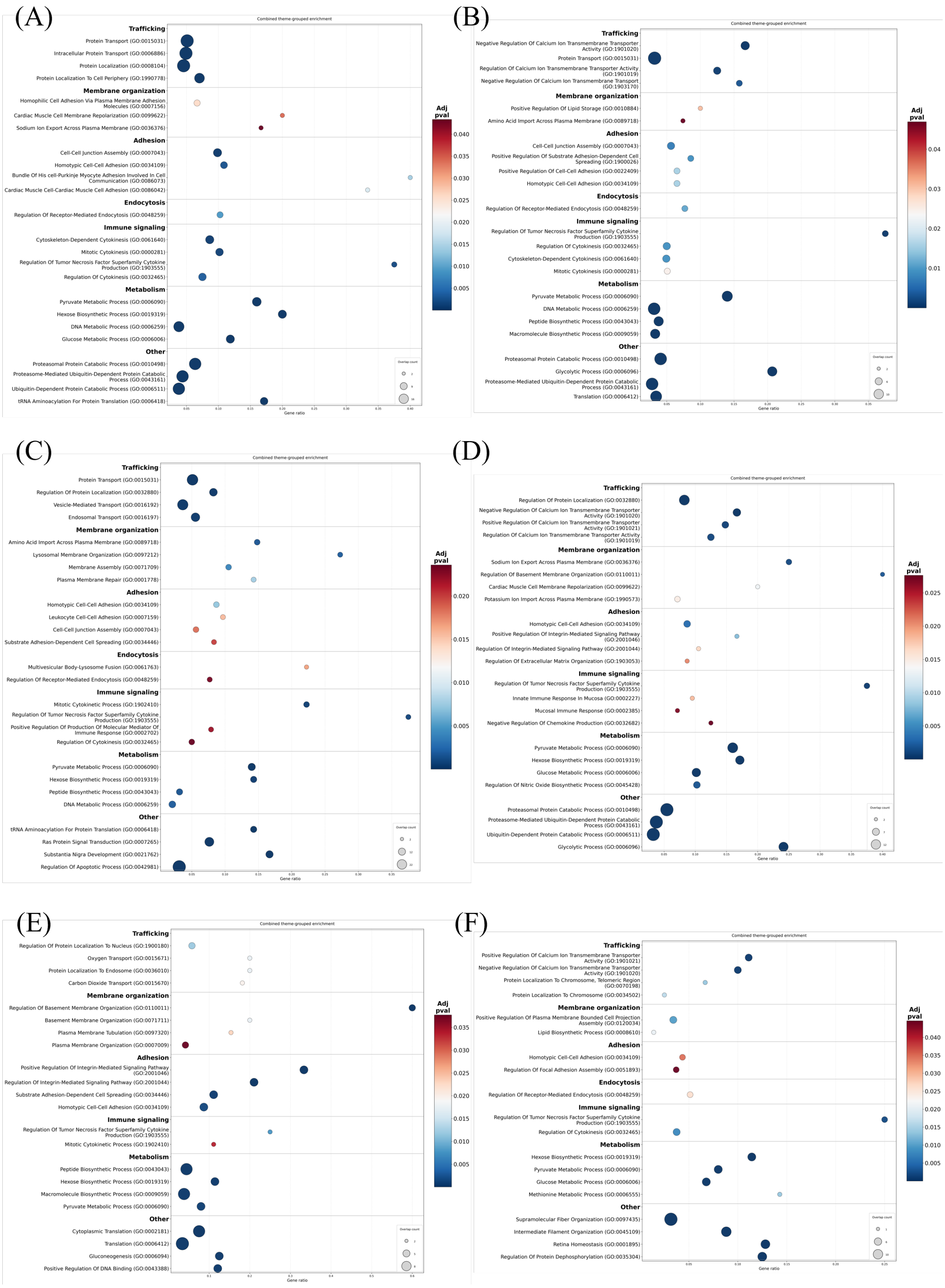

Figure S6. Functional enrichment of EV proteomes across VAD-engineered conditions. (A–F) Dot plots showing grouped GO Biological Process enrichment derived from significantly dysregulated EV proteins relative to WT EVs for each construct: (A) PTGFRN, (B) CD63, (C) LAMP2B, (D) MYR(SRC), (E) MYR(CHMP6), and (F) PPC. The x-axis represents the gene

ratio (proportion of proteins associated with each term), while dot color indicates the adjusted p value (Benjamini–Hochberg corrected; warmer colors denote higher significance). Dot size reflects the overlap count (number of EV proteins mapped to each term). Enriched terms are grouped into functional themes (left axis), including protein trafficking, membrane organization, adhesion, endocytosis, immune signaling, and metabolism, representing core processes linked to EV biogenesis and function.

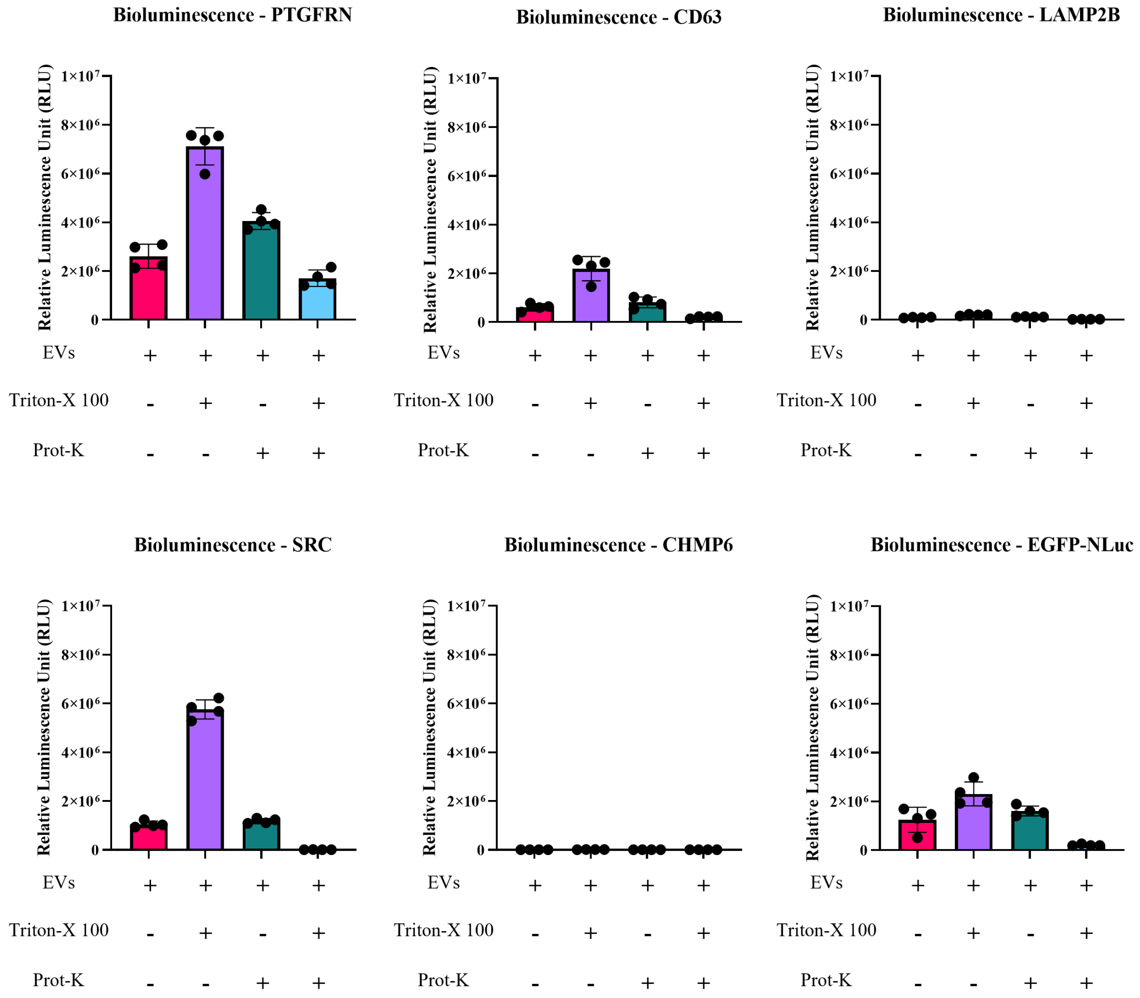

Figure S7: Evaluation of the of luminescence emitted from Nanoluciferase activity in engineered EVs. Proteinase and detergent treatments were performed separately and simultaneously on equal numbers of EVs to confirm that NLuc activity originated from within the EV lumen.

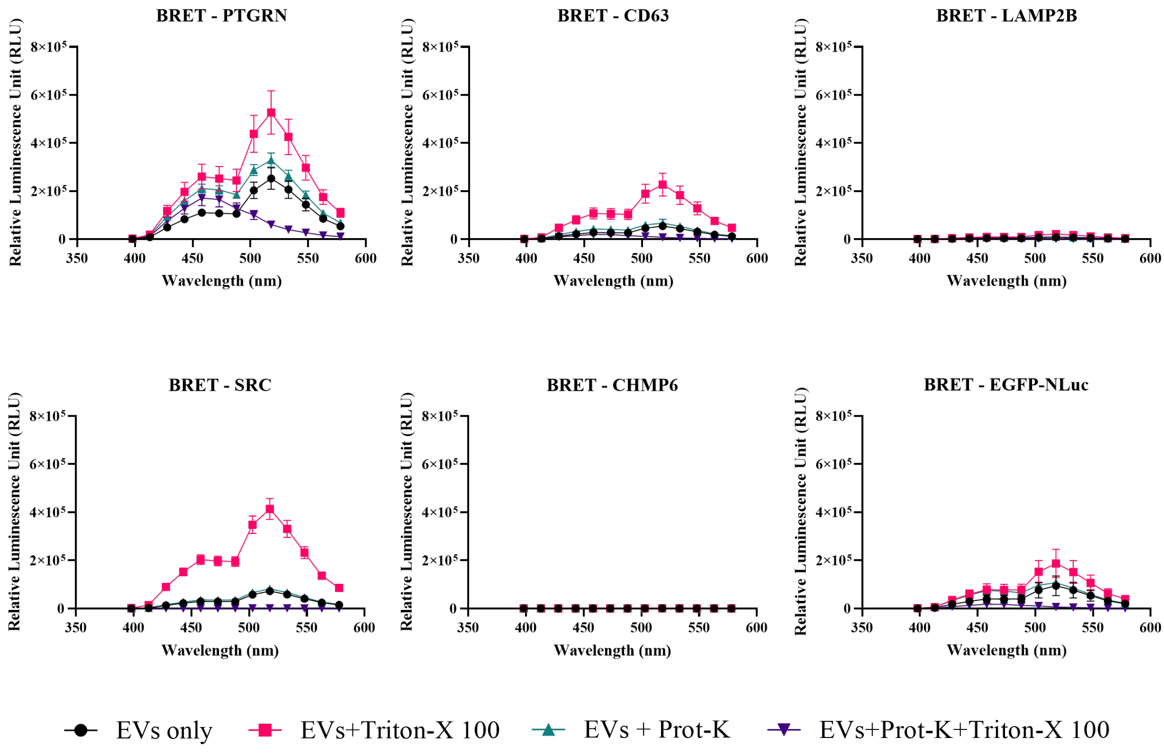

Figure S8: Evaluation of spectral luminescence to assess bioluminescence resonance energy transfer in engineered EVs. Proteinase and detergent treatments were performed separately and simultaneously on equal numbers of EVs to confirm the proximity of EGFP-NLuc within the EV lumen.

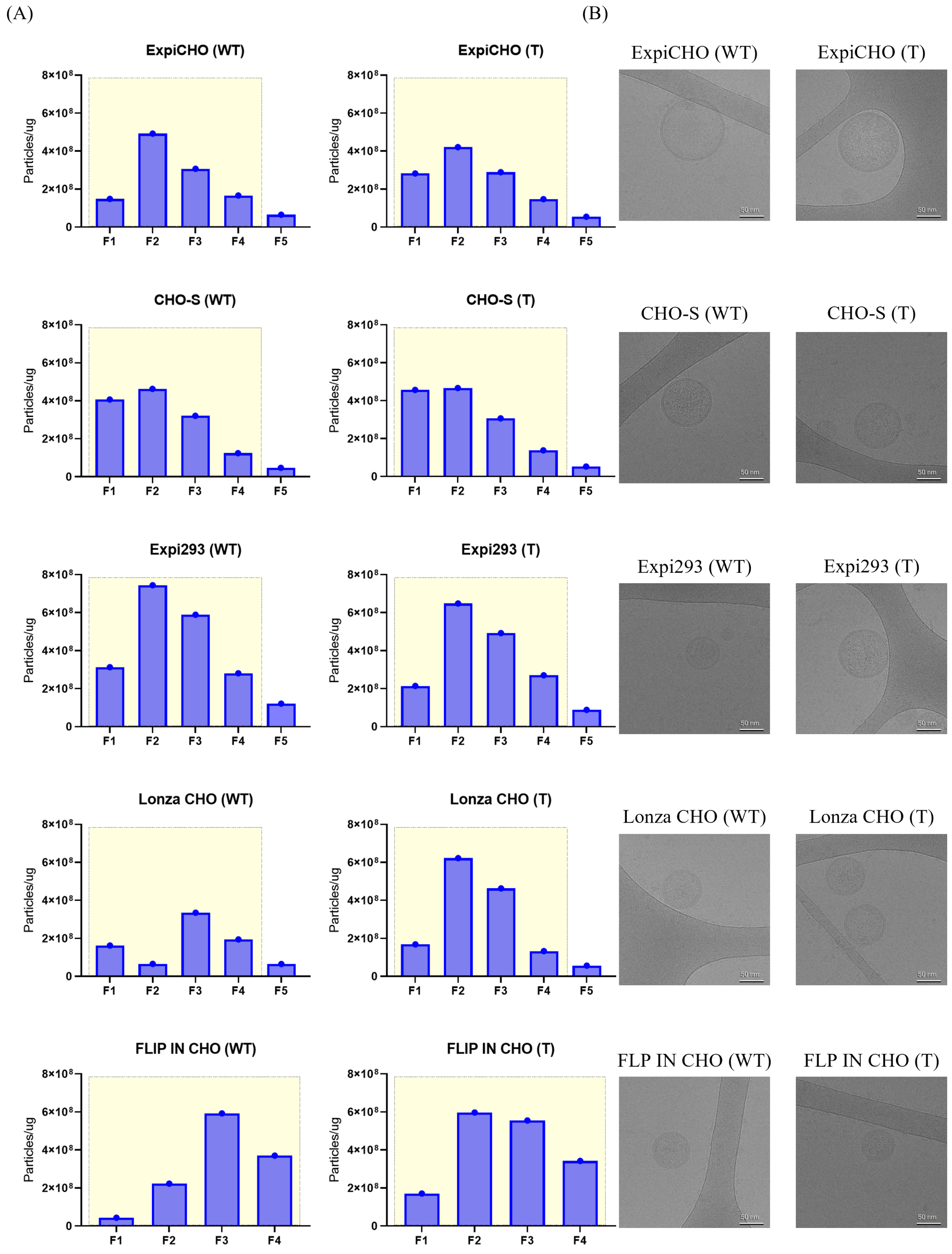

Figure S9: Characterization of EVs isolated through size exclusion chromatography. (A) Particle-to-protein ratio across SEC fractions, highlighting enrichment of EVs in fractions 1 to 4. (B) Cryo-Transmission electron microscopy images of isolated EVs showing intact lipid bilayer structures and spherical morphology.

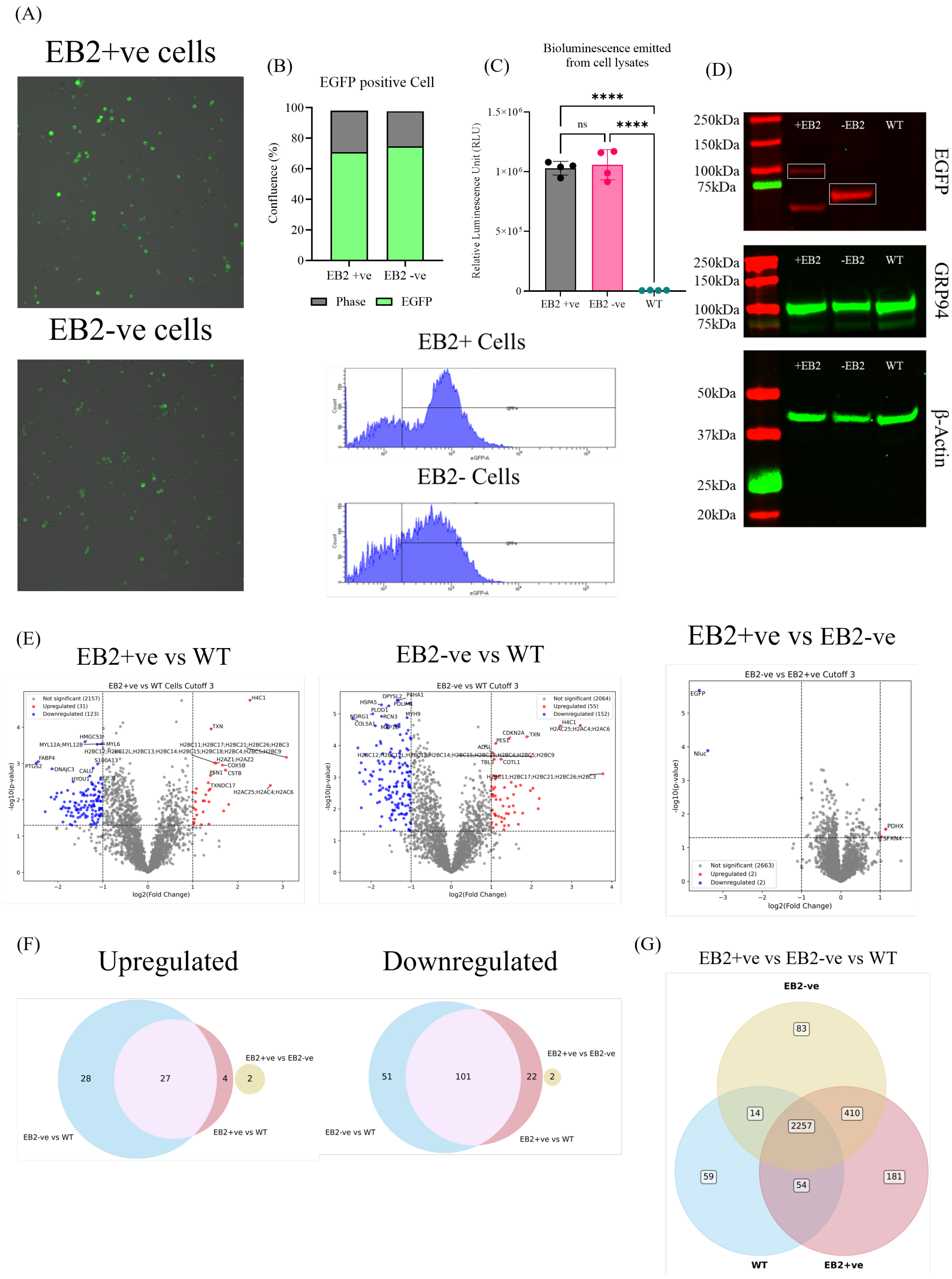

Figure S10: Analysis of stable ExpiCHO cell lines expressing EGFP-NLuc fusion proteins. (A) Confocal microscopy images of engineered ExpiCHO cells showing EGFP expression. (B) Flow cytometry analysis demonstrating enrichment of EGFP-positive cells following antibiotic selection. (C) Bioluminescence quantification indicating comparable NLuc expression levels for EB2^{+ve} and EB2^{-ve} cell lines. (D) Immunoblot analysis confirming EGFP-NLuc fusion

protein expression, with GRP94 and ACTB1 as loading controls. (E) Comparative analyses of differential protein expression is shown as volcano plots, performed to assess stable integration specific changes in cell-protein abundance. (F) Venn diagrams showing the overlap of differentially expressed proteins between engineered and WT cells. Comparison of EB2^{+ve} and EB2^{-ve} cells identified distinct sets of upregulated and downregulated proteins, alongside proteins commonly altered in both stable line groups relative to wild-type cells. (G) Proteomic profiling of cells. Global proteomic analysis conducted to identify proteins shared among engineered and WT cells, unique to specific groups, and common between selected pairs. Statistical significance was assessed using one-way ANOVA with multiple comparison performed using Tukey's tests, with $P < 0.05$ considered significant. * ($P \leq 0.05$), ** ($P \leq 0.01$), *** ($P \leq 0.001$), **** ($P \leq 0.0001$).

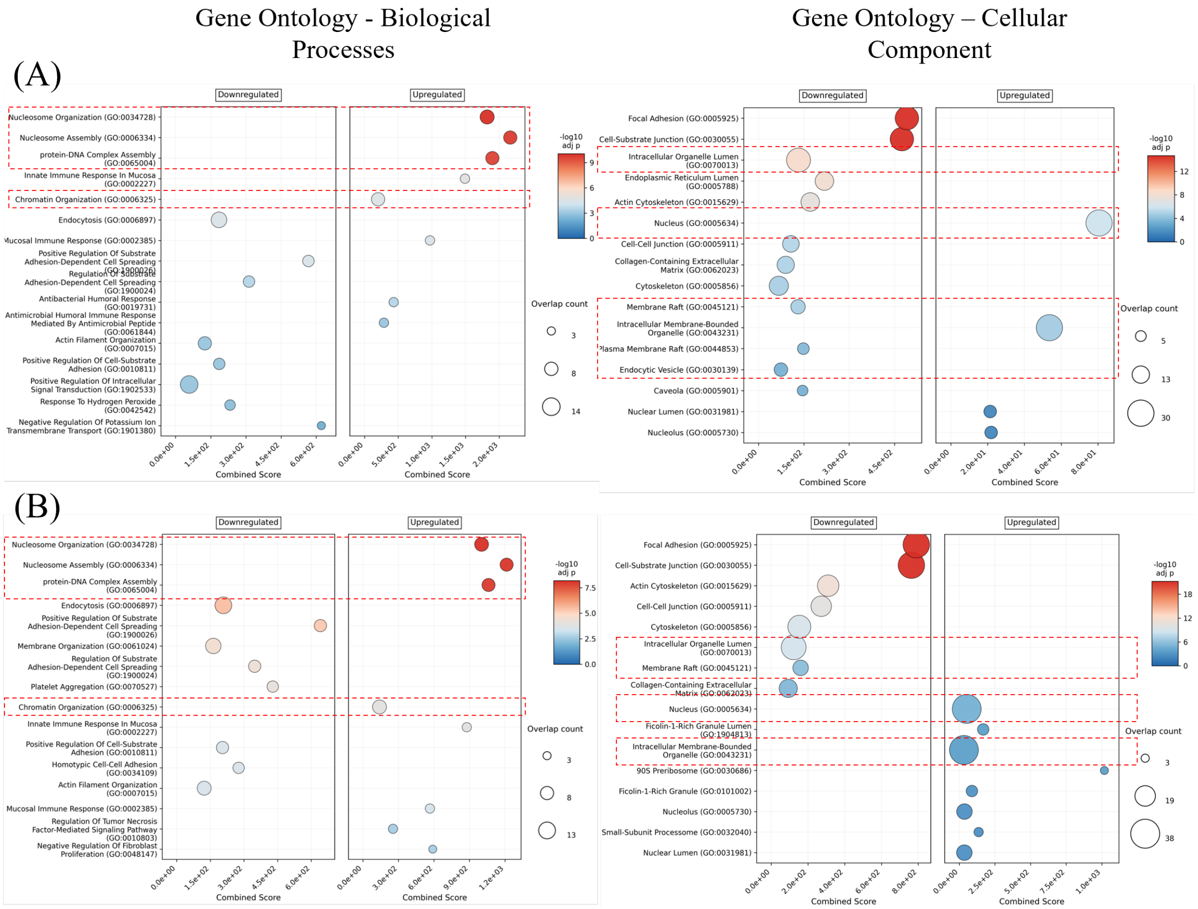

Figure S11. Functional enrichment of proteomic changes in stable engineered CHO cell lines.(A–B) Dot plots showing enriched GO terms derived from significantly dysregulated proteins relative to WT cells for (A) EB2^{+ve} and (B) EB2^{-ve} stable CHO cell lines. Proteins are separated into downregulated (left) and upregulated (right) groups. The x-axis represents the combined enrichment score (integrating enrichment magnitude and statistical significance). Dot color indicates –log10 adjusted p value (Benjamini–Hochberg corrected; higher values denote greater significance), and dot size reflects the overlap count (number of proteins associated with each term). Highlighted clusters (red dashed boxes) indicate dominant functional categories.

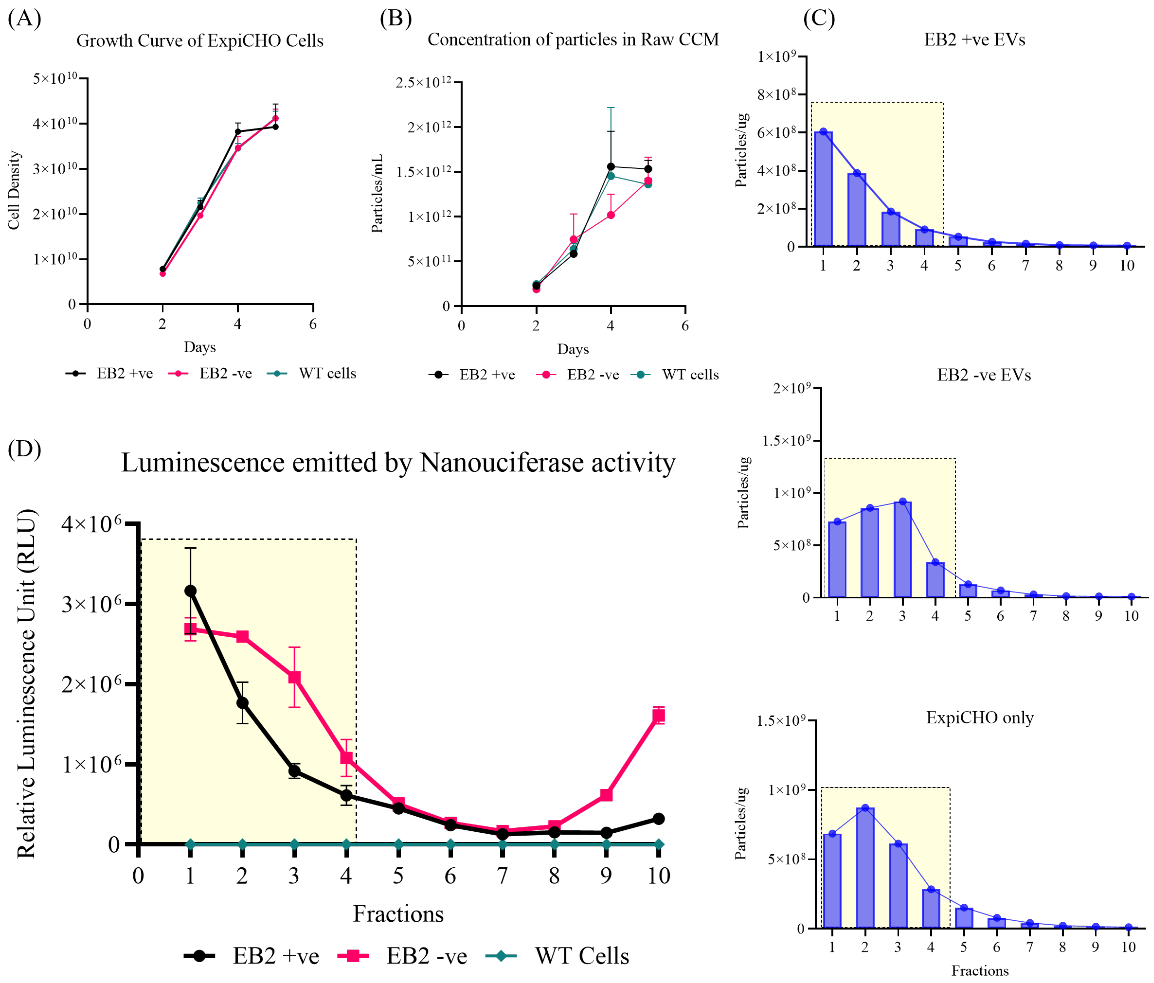

Figure S12: Evaluation of cell growth, particle production, and EV isolation during scaled-up culture. (A) Viable cell density over a 5-day culture period for control and engineered cells. (B) Nanoparticle tracking analysis (NTA) of unprocessed conditioned culture media (CCM) collected periodically to monitor particle concentration. (C) Particle-to-protein ratio across size exclusion chromatography (SEC) fractions to identify EV-enriched fractions. (D) Bioluminescence analysis of SEC fractions confirming EGFP-NLuc-enriched EVs. Statistical significance was assessed using two-way ANOVA with multiple comparison performed using Tukey's tests, with $P < 0.05$ considered significant.

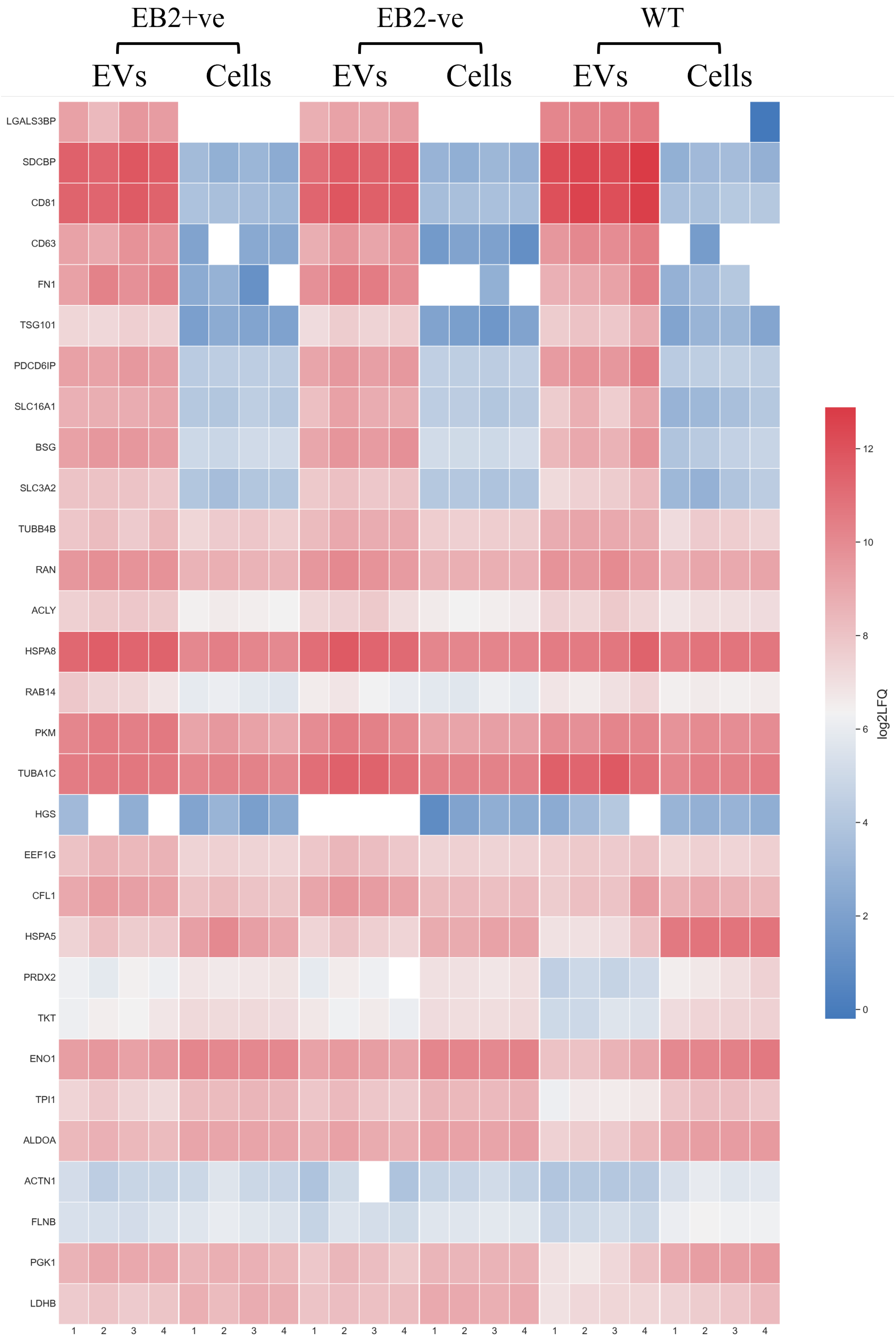
EB2+ve
EB2-ve
WT
EVs
Cells
EVs
Cells
EVs
Cells
LGALS3BP
SDCBP
CD81
CD63
FN1
TSG101
PDCD6IP
SLC16A1
BSG
SLC3A2
TUBB4B
RAN
ACLY
HSPA8
RAB14
PKM
TUBA1C
HGS
EEF1G
CFL1
HSPA5
PRDX2
TKT
ENO1
TPI1
ALDOA
ACTN1
FLNB
PGK1
LDHB
1 2 3 4 1 2 3 4 1 2 3 4 1 2 3 4 1 2 3 4 1 2 3 4
log2LFQ
0
2
4
6
8
10
12

Figure S13: Heatmap for the expression levels of EV specific markers in the EVs and their corresponding producer cells. Expression of majority of EV-specific canonical markers was elevated in EVs when compared to their corresponding producer cells. The expression levels for all the identified markers were comparable in engineered and WT EVs.

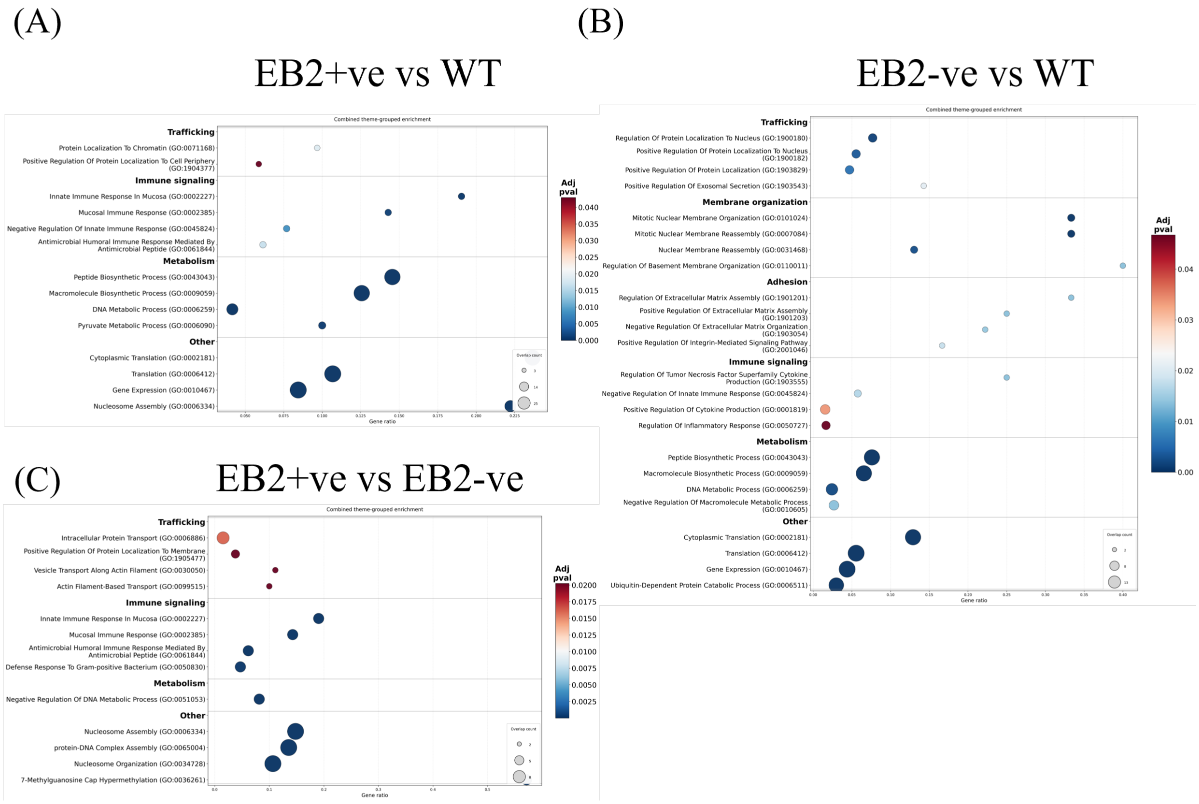

Figure S14. Functional enrichment of EV proteomes from stable engineered CHO cell lines. (A–C) Dot plots showing grouped GO Biological Process enrichment derived from significantly dysregulated EV proteins for (A) EB2^{+ve} vs WT, (B) EB2^{-ve} vs WT, and (C) EB2^{+ve} vs EB2^{-ve} comparisons. The x-axis represents the gene ratio (proportion of proteins associated with each term). Dot color indicates the adjusted p value (Benjamini–Hochberg corrected; warmer colors denote higher significance), and dot size reflects the overlap count (number of EV proteins mapped to each term). Across conditions, enriched terms cluster into shared functional themes including translation, gene expression, DNA metabolic processes, intracellular trafficking, immune signaling, and metabolism, indicating a conserved engineered EV signature.

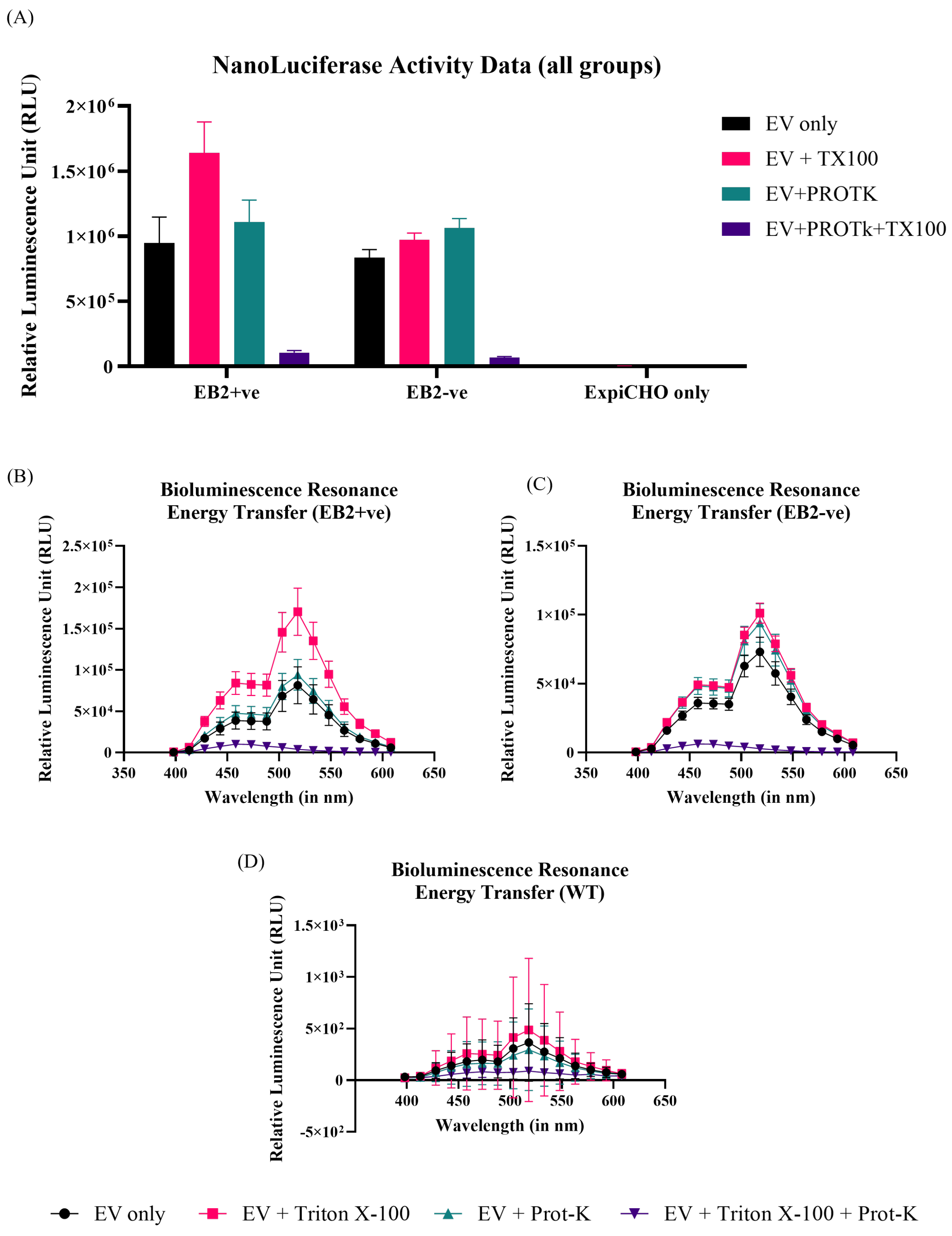

Figure S15: Evaluation of the luminescence emitted by Nanoluciferase activity and EGFP fluorescence. (A) Nanoluciferase activity for EVs derived from stable ExpiCHO and WT cells (B-D) Evaluation of luminescence emitted over spectral range in (B) engineered EVs displaying Ephrin-B2 ligand on their surface, (C) in engineered EVs lacking Ephrin-B2 ligand on their surface, and (D) in EVs derived from WT cells. To confirm the intraluminal localization of cargo Proteinase and detergent treatments were performed separately and simultaneously on equal numbers of EVs

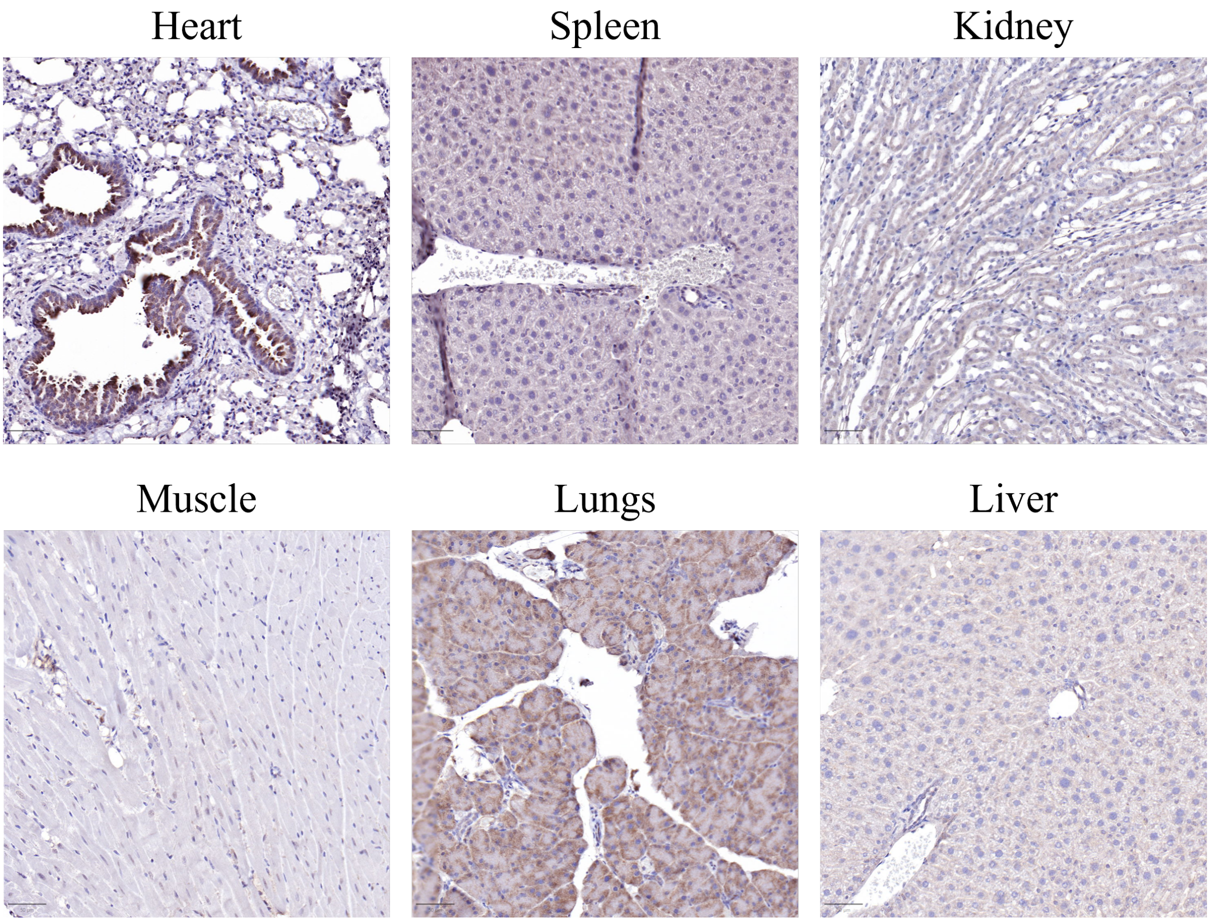

Figure S16: Immunohistochemical analysis performed to validate the expression of Ephrin-B4 in non-cancerous organs (heart, spleen, kidney, muscles, lungs, and liver) obtained from the mice injected with orthotopic PDX implant.

Open Filter – Total Luminescence

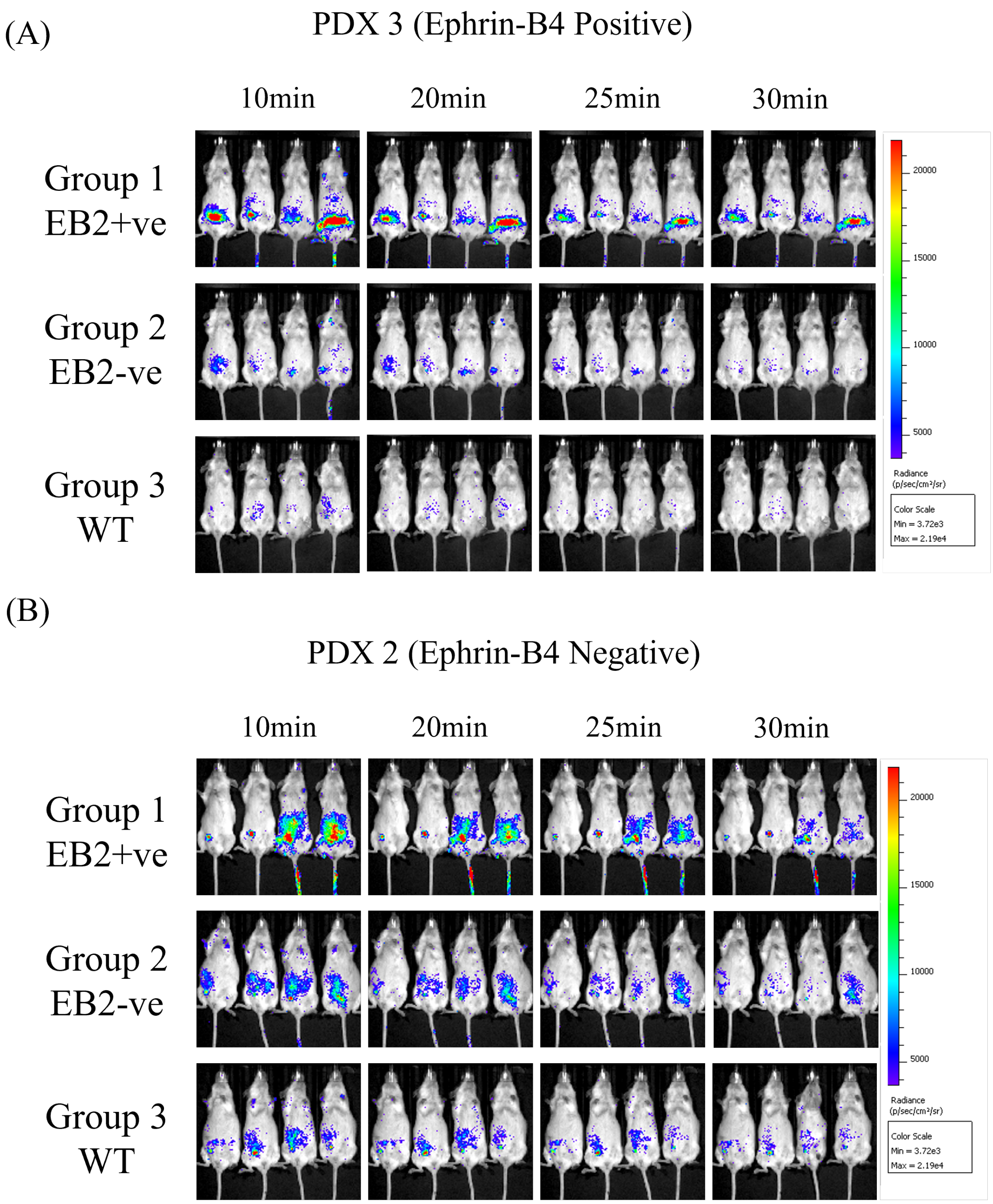

Figure S17: *In vivo* imaging for assessing biodistribution of eEVs using total luminescence emitted by NLuc and EGFP. (A) *In vivo* imaging performed for PDXs expressing Ephrin-B4 receptor (PDX3) for assessing the biodistribution eEVs. (B) *In vivo* imaging performed for PDXs lacking the expression Ephrin-B4 receptor (PDX2) for assessing the biodistribution eEVs.

500nm Filter – NLuc Luminescence

(A)

PDX 3 (Ephrin-B4 Positive)

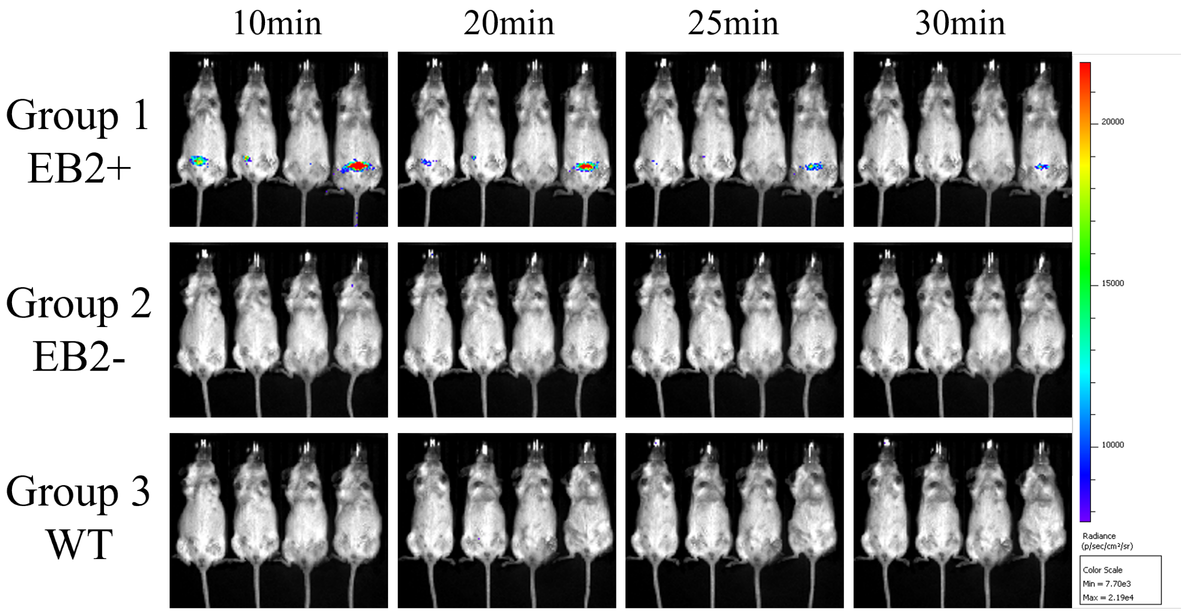

PDX 2 (Ephrin-B4 Negative)

(B)

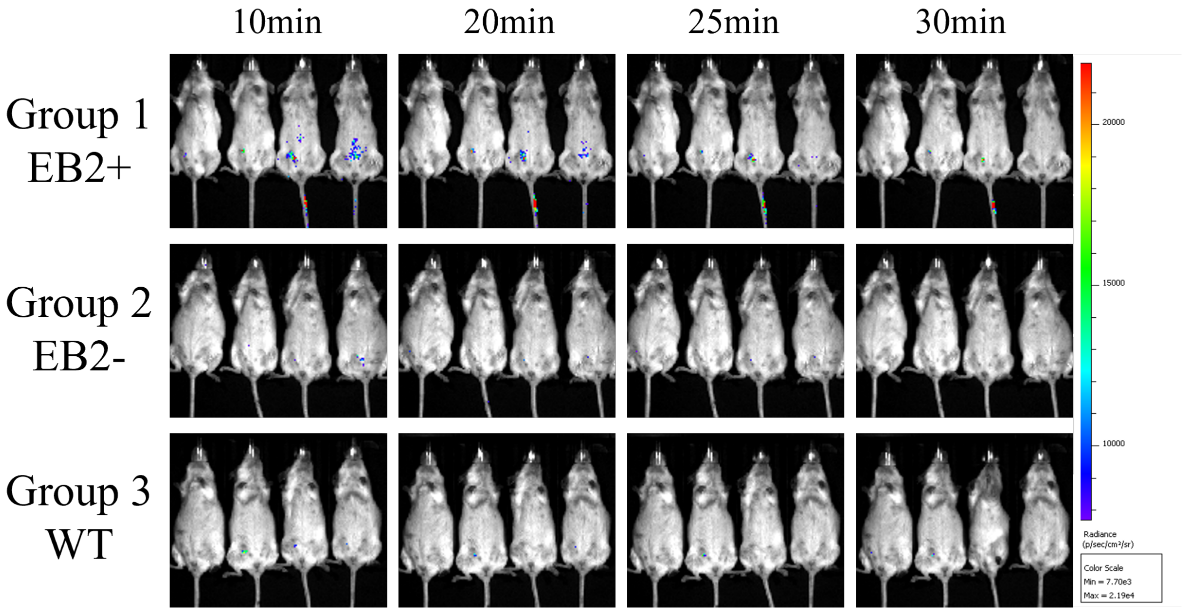

Figure S18: *In vivo* imaging for assessing biodistribution of eEVs via luminescence emitted by NLuc activity. (A) *In vivo* imaging performed for PDXs expressing Ephrin-B4 receptor (PDX3) for assessing the biodistribution eEVs. (B) *In vivo* imaging performed for PDXs lacking the expression Ephrin-B4 receptor (PDX2) for assessing the biodistribution eEVs.

520nm Filter - EGFP Fluorescence

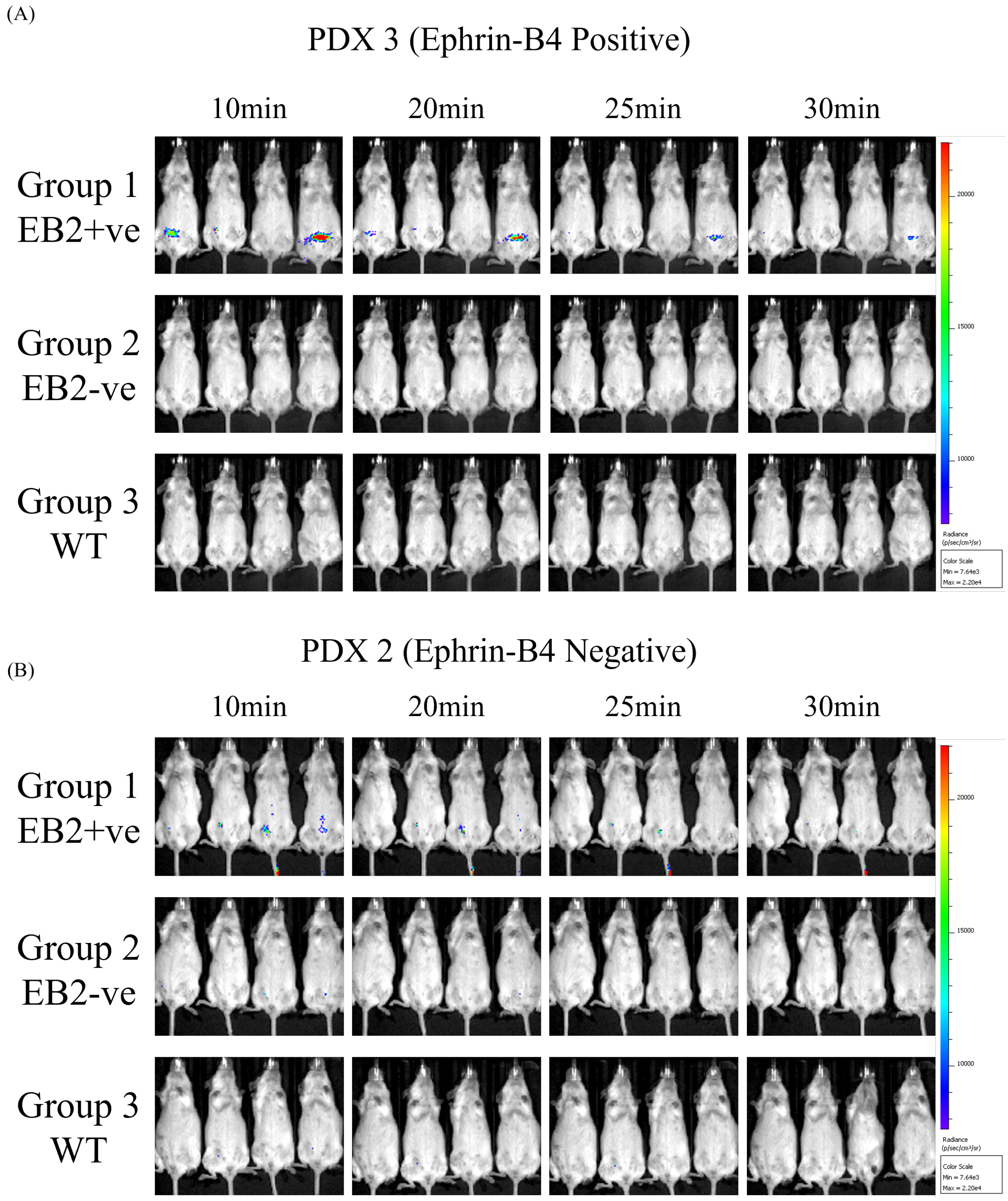

Figure S19: *In vivo* imaging for assessing biodistribution of eEVs via fluorescence emitted by EGFP. (A) *In vivo* imaging performed for PDXs expressing Ephrin-B4 receptor (PDX3) for assessing the biodistribution eEVs. (B) *In vivo* imaging performed for PDXs lacking the expression Ephrin-B4 receptor (PDX2) for assessing the biodistribution eEVs.

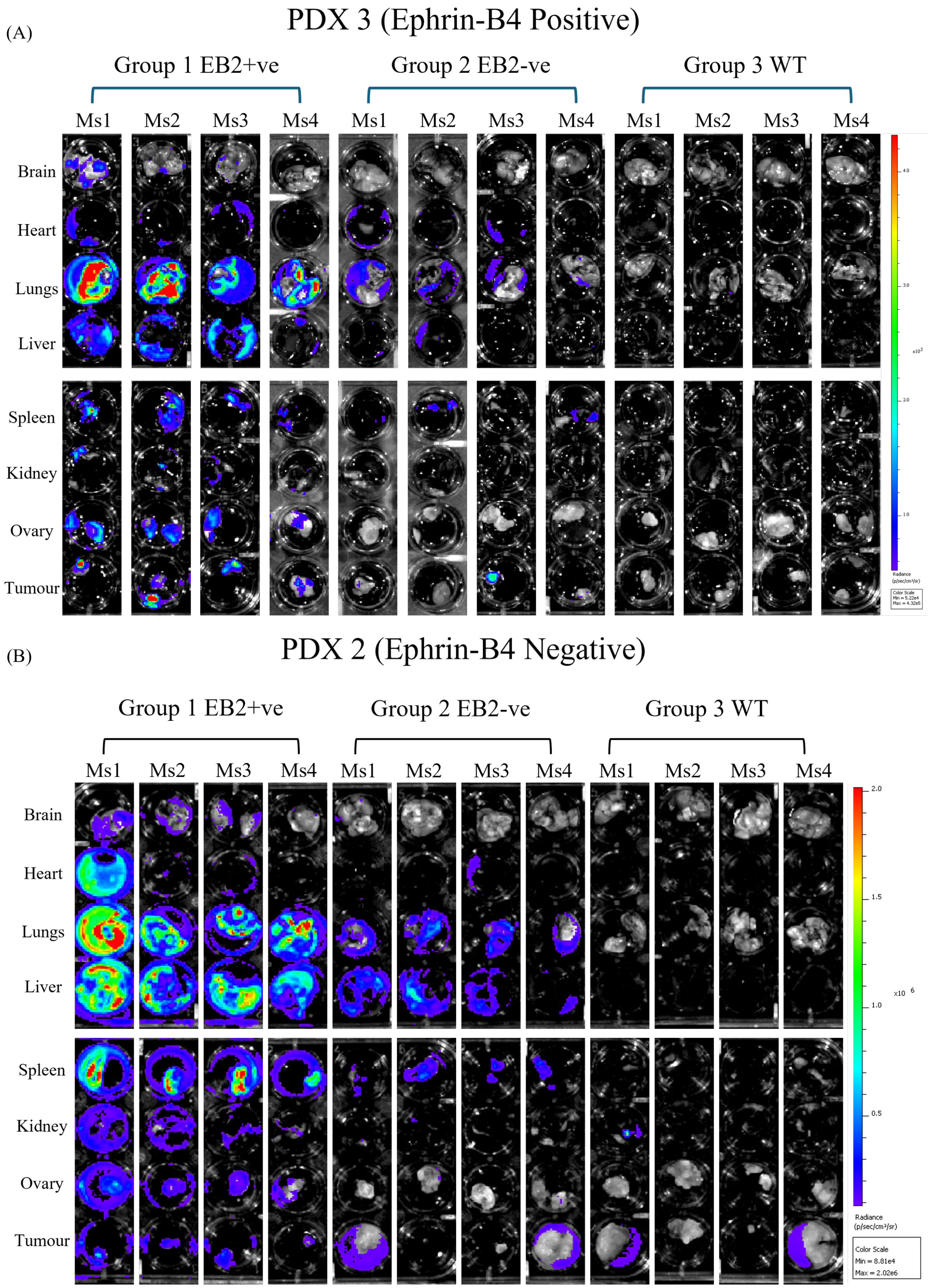

Figure S20: *Ex vivo* imaging for assessing biodistribution of eEVs using total luminescence emitted by NLuc and EGFP. (A) *Ex vivo* imaging performed for the organs harvested from the PDXs expressing Ephrin-B4 receptor (PDX3) for assessing the biodistribution eEVs. (B) *Ex*

vivo imaging performed for the organs harvested from the PDXs lacking the expression Ephrin-B4 receptor (PDX2) for assessing the biodistribution eEVs.

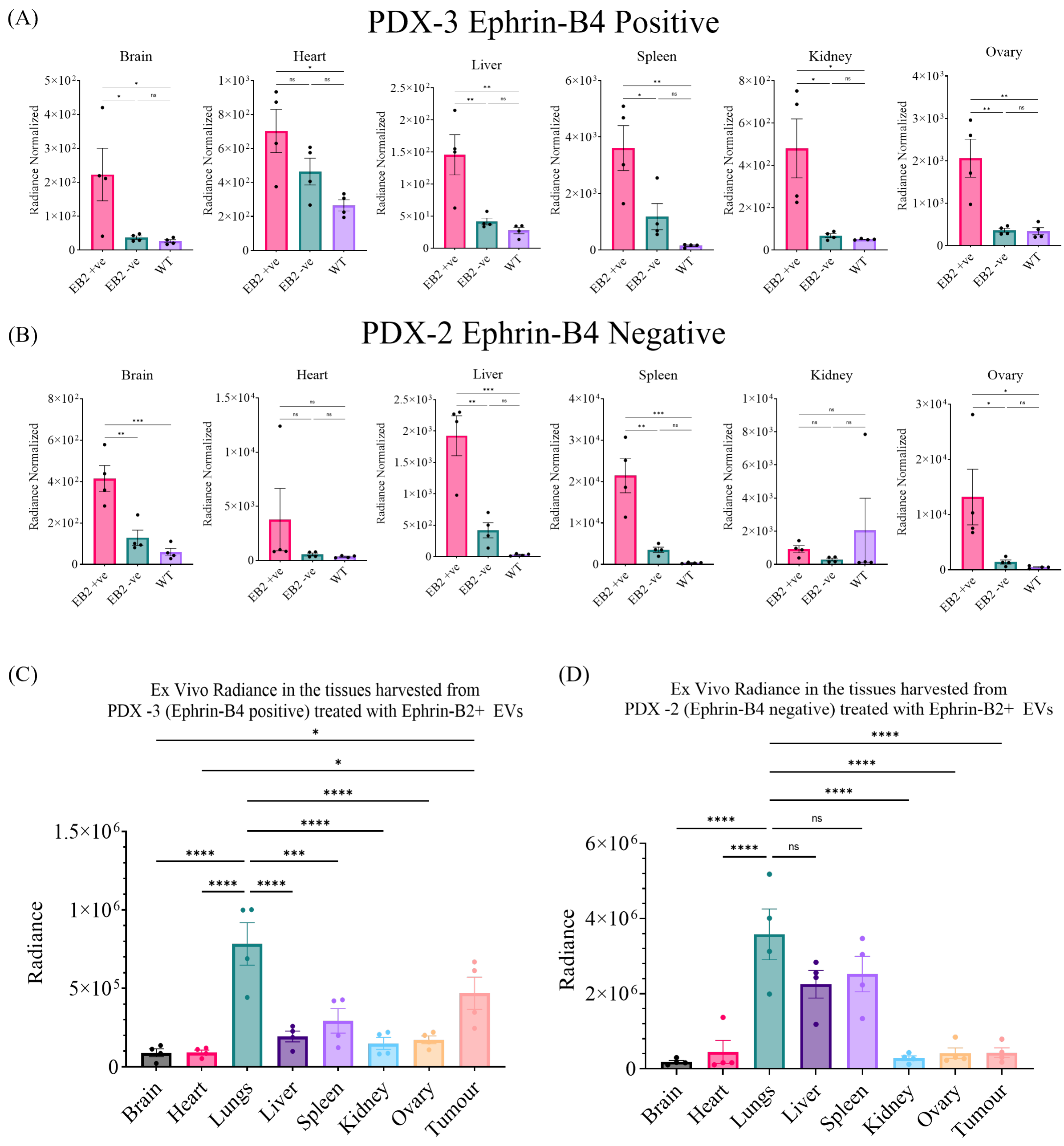

Figure S21: *Ex vivo* biodistribution analysis of the organs harvested from the PDX-bearing mice injected with the eEVs packaged with EGFP-NLuc. (A-B) Quantitative analysis of maximum radiance from *ex vivo* total luminescence imaging of harvested organs (brain, heart, liver, spleen, kidney, and ovary) to assess organ-specific localization of administered eEVs. (A) maximum radiance from *ex vivo* imaging of organs harvested from PDXs lacking the expression Ephrin-B4 receptor (PDX2). (B) maximum radiance from *ex vivo* imaging of organs harvested from PDXs lacking the expression Ephrin-B4 receptor (PDX2). (C) Quantitative analysis of *ex vivo* radiance emitted from the organs and tissues harvested from PDX expressing Ephrin-B4 receptor (PDX3). Lungs were identified to emit significantly higher radiance compared to other harvested organs and tissues. (D) Quantitative analysis of *ex vivo* radiance

emitted from the organs and tissues harvested from PDX lacking the expression Ephrin-B4 receptor (PDX2). Lungs were identified to emit significantly higher radiance compared to other harvested organs and tissues such as brain, heart, kidney, ovary, and tumor, but were comparable to liver and spleen, suggesting a broader nonspecific distribution pattern. Statistical significance was assessed using one-way ANOVA with multiple comparison performed using Tukey's tests, with $P < 0.05$ considered significant. * ($P \leq 0.05$), ** ($P \leq 0.01$), *** ($P \leq 0.001$), **** ($P \leq 0.0001$).

Supplementary Table 1. The translated sequences for all the transgenes used in this study

Plasmid Number	Translated Fusion Protein	Translated Amino Acid Sequence
Plasmid 1	EB2-PTGFRN-EGFP-Nluc<br>(~96kDa)	MAVRRDSVWKYCWGVLMVLCRTAISKSIVLEPIYWNSSNSKFLPGQGLVLYPQIGDKLDIICPK<br>VDSKTVGQYEYYKVYMVDKDQADRCTIKKENTPLLNCAKPDQDIKFTIKFQEFSPNLWGLEFQK<br>NKDYYIISTSNGSLEGLDNQEGGVCQTRAMKILMKVGQDASSAGSTRNKDPTRRPELEAGTNGR<br>SSTTSPFVKPNPGSSTDGNSAGHSGNNILGSEVALFAGGGGSEQKLISEEDLGGGGSKGGGGSP<br>IFNASVHSDTPSVTRGDLIKLFCIVTLEGAVLDPDDMAFDVSWFAVHSFGLDKDPVLLSSLDRK<br>GVVTTGQRDWKSTLSLERVSVLEFLLQVHGSEDQDFGNYYCSVTPWVRSPTGSWQREAEIHSRP<br>IFITVKMDVLNAFKYPLLIGVGLSTVIGLLSCLIGYCSSHWCCKKEVRETRRERRRLMSMEMDG<br>GGGSVDVSKGEELFTGVVPILVELDGDVNGHKFSVSGEGEGDATYGKLTLKFICTTGKLPVPWP<br>TLVTTLTYGVQCFSRYPDHMKQHDFFKSAMPEGYVQERTIFFKDDGNYKTRAEVKFEGDTLVNR<br>IELKGIDFKEDGNILGHKLEYNYNSHNVYIMADKQKNGIKVNFKIRHNIEDGSVQLADHYQQNT<br>PIGDGPVLLPDNHYLSTQSALSKDPNEKRDHMVLLEFVTAAGITLGMDELYKDISGGVFTLEDF<br>VGDWRQTAGYNLDQVLEQGGVSSLFQNLGVSVTPIQRIVLSGENGLKIDIHVIIPYEGLSGDQM<br>GQIEKIFKVVYPVDDHHFKVILHYGTLVIDGVTPNMIDYFGRPYEGIAVFDGKKITVTGTLWNG<br>NKIIDERLINPDGSLLFRVTINGVTGWRLCERILA***
Plasmid 2	EB2-CD63-EGFP-Nluc<br>(~100kDa)	MAVEGGMKCVKFLLYVLLLAFCACAVGLIAVGVGAQLVLSQTIIQGATPGSLLPVVIIAVGVFL<br>FLVAFVGCCGACKENYCLMITFAIFLSLIMLVEVAAAIAGYVFRDKVMSEFNNNFRQQMENYPK<br>NNHTASILDRMQADFKCCGAANYTDWEKIPSMSKNRVPDSCCINVTVGCGINFNEKAIHKEGCV<br>EKIGGWLGGGGSMAVRRDSVWKYCWGVLMVLCRTAISKSIVLEPIYWNSSNSKFLPGQGLVLYP<br>QIGDKLDIICPKVDSKTVGQYEYYKVYMVDKDQADRCTIKKENTPLLNCAKPDQDIKFTIKFQE<br>FSPNLWGLEFQKNKDYYIISTSNGSLEGLDNQEGGVCQTRAMKILMKVGQDASSAGSTRNKDPT<br>RRPELEAGTNGRSSTTSPFVKPNPGSSTDGNSAGHSGNNILGSEVALFAGGGGSEQKLISEEDL<br>GGGGSRKNVLVVAAAALGIAFVEVLGIVFACCLVKSIRSGYEVMGGGGSVDVSKGEELFTGVVP<br>ILVELDGDVNGHKFSVSGEGEGDATYGKLTLKFICTTGKLPVPWPTLVTTLTYGVQCFSRYPDH<br>MKQHDFFKSAMPEGYVQERTIFFKDDGNYKTRAEVKFEGDTLVNRIELKGIDFKEDGNILGHKL<br>EYNYNSHNVYIMADKQKNGIKVNFKIRHNIEDGSVQLADHYQQNTPIGDGPVLLPDNHYLSTQS<br>ALSKDPNEKRDHMVLLEFVTAAGITLGMDELYKDISGGVFTLEDFVGDWRQTAGYNLDQVLEQG<br>GVSSLFQNLGVSVTPIQRIVLSGENGLKIDIHVIIPYEGLSGDQMGQIEKIFKVVYPVDDHHFK<br>VILHYGTLVIDGVTPNMIDYFGRPYEGIAVFDGKKITVTGTLWNGNKIIDERLINPDGSLLFRV<br>TINGVTGWRLCERILA***

**Supplementary Table 1. The translated sequences for all the transgenes used in this study**		
**Plasmid Number**	**Translated Fusion Protein**	**Translated Amino Acid Sequence**
Plasmid 3	EB2-LAMP2B-EGFP-Nluc (~117kDa)	MVCFRLFPVPGSGLVLVCLVLGAVRSYALELNLTDSENATCLYLEMAVRRDSVWKYCWGVLMVL<br>CRTAISKSIVLEPIYWNSSNSKFLPGQGLVLYPQIGDKLDIICPKVDSKTVGQYEYYKVYMVDK<br>DQADRCTIKKENTPLLNCAKPDQDIKFTIKFQEFSPNLWGLEFQKNKDYYIISTSNGSLEGLDN<br>QEGGVCQTRAMKILMKVGQDASSAGSTRNKDPTRRPELEAGTNGRSSTTSPFVKPNPGSSTDGN<br>SAGHSGNNILGSEVALFASGGAKWQMNFTVRYETTNKTYKTVTISDHGTVTYNGSICGDDQNGP<br>KIAVQFGPGFSWIANFTKAASTYSIDSVSFSYNTGDNTTFPDAEDKGILTVDELLAIRIPLNDL<br>FRCNSLSTLEKNDVVQHYWDVLVQAFVQNGTVSTNEFLCDKDKTSTVAPTIHTTVPSPTTTPTP<br>KEKPEAGTYSVNNGNDTCLLATMGLQLNITQDKVASVININPNTTHSTGSCRSHTALLRLNSST<br>IKYLDFVFAVKNENRFYLKEVNISMYLVNGSVFSIANNNLSYWDAPLGSSYMCNKEQTVSVSGA<br>FQINTFDLRVQPFNVTQGKYSTAQECSLDDDTILIPIIVGAGLSGLIIVIVIAYVIGRRKSYAG<br>YQTLGSVDVSKGEELFTGVVPILVELDGDVNGHKFSVSGEGEGDATYGKLTLKFICTTGKLPVP<br>WPTLVTTLTYGVQCFSRYPDHMKQHDFFKSAMPEGYVQERTIFFKDDGNYKTRAEVKFEGDTLV<br>NRIELKGIDFKEDGNILGHKLEYNYNSHNVYIMADKQKNGIKVNFKIRHNIEDGSVQLADHYQQ<br>NTPIGDGPVLLPDNHYLSTQSALSKDPNEKRDHMVLLEFVTAAGITLGMDELYKDISGGVFTLE<br>DFVGDWRQTAGYNLDQVLEQGGVSSLFQNLGVSVTPIQRIVLSGENGLKIDIHVIIPYEGLSGD<br>QMGQIEKIFKVVYPVDDHHFKVILHYGTLVIDGVTPNMIDYFGRPYEGIAVFDGKKITVTGTLW<br>NGNKIIDERLINPDGSLLFRVTINGVTGWRLCERILA***
Plasmid 4	MYR(SRC)-EGFP-Nluc (~47kDa)	MGSNKSKPKVSKGEELFTGVVPILVELDGDVNGHKFSVSGEGEGDATYGKLTLKFICTTGKLPV<br>PWPTLVTTLTYGVQCFSRYPDHMKQHDFFKSAMPEGYVQERTIFFKDDGNYKTRAEVKFEGDTL<br>VNRIELKGIDFKEDGNILGHKLEYNYNSHNVYIMADKQKNGIKVNFKIRHNIEDGSVQLADHYQ<br>QNTPIGDGPVLLPDNHYLSTQSALSKDPNEKRDHMVLLEFVTAAGITLGMDELYKDISGGVFTL<br>EDFVGDWRQTAGYNLDQVLEQGGVSSLFQNLGVSVTPIQRIVLSGENGLKIDIHVIIPYEGLSG<br>DQMGQIEKIFKVVYPVDDHHFKVILHYGTLVIDGVTPNMIDYFGRPYEGIAVFDGKKITVTGTL<br>WNGNKIIDERLINPDGSLLFRVTINGVTGWRLCERILA***

Supplementary Table 1. The translated sequences for all the transgenes used in this study		
**Plasmid Number**	**Translated Fusion Protein**	**Translated Amino Acid Sequence**
Plasmid 5	MYR(CHMP6)-EGFP-Nluc (~47kDa)	MGNLFGRKKQSRVSKGEELFTGVVPILVELDGDVNGHKFSVSGEGEGDATYGKLTLKFICTTGK<br>LPVPWPTLVTTLTYGVQCFSRYPDHMKQHDFFKSAMPEGYVQERTIFFKDDGNYKTRAEVKFEG<br>DTLVNRIELKGIDFKEDGNILGHKLEYNYNSHNVYIMADKQKNGIKVNFKIRHNIEDGSVQLAD<br>HYQQNTPIGDGPVLLPDNHYLSTQSALSKDPNEKRDHMVLLEFVTAAGITLGMDELYKDISGGV<br>FTLEDFVGDWRQTAGYNLDQVLEQGGVSSLFQNLGVSVTPIQRIVLSGENGLKIDIHVIIPYEG<br>LSGDQMGQIEKIFKVVYPVDDHHFKVILHYGTLVIDGVTPNMIDYFGRPYEGIAVFDGKKITVT<br>GTLWNGNKIIDERLINPDGSLLFRVTINGVTGWRLCERILA***
Plasmid 6	EGFP-Nluc (~47kDa)	MVSKGEELFTGVVPILVELDGDVNGHKFSVSGEGEGDATYGKLTLKFICTTGKLPVPWPTLVTT<br>LTYGVQCFSRYPDHMKQHDFFKSAMPEGYVQERTIFFKDDGNYKTRAEVKFEGDTLVNRIELKG<br>IDFKEDGNILGHKLEYNYNSHNVYIMADKQKNGIKVNFKIRHNIEDGSVQLADHYQQNTPIGDG<br>PVLLPDNHYLSTQSALSKDPNEKRDHMVLLEFVTAAGITLGMDELYKDISGGVFTLEDFVGDWR<br>QTAGYNLDQVLEQGGVSSLFQNLGVSVTPIQRIVLSGENGLKIDIHVIIPYEGLSGDQMGQIEK<br>IFKVVYPVDDHHFKVILHYGTLVIDGVTPNMIDYFGRPYEGIAVFDGKKITVTGTLWNGNKIID<br>ERLINPDGSLLFRVTINGVTGWRLCERILA***
Plasmid 7	EB2-PTGFRN-EGFP (~75kDa)	MAVRRDSVWKYCWGVLMVLCRTAISKSIVLEPIYWNSSNSKFLPGQGLVLYPQIGDKLDIICPK<br>VDSKTVGQYEYYKVYMVDKDQADRCTIKKENTPLLNCAKPDQDIKFTIKFQEFSPNLWGLEFQK<br>NKDYYIISTSNGSLEGLDNQEGGVCQTRAMKILMKVGQDASSAGSTRNKDPTRRPELEAGTNGR<br>SSTTSPFVKPNPGSSTDGNSAGHSGNNILGSEVALFAGGGGSEQKLISEEDLGGGGSKGGGGSP<br>IFNASVHSDTPSVTRGDLIKLFCIVTLEGAVLDPDDMAFDVSWFAVHSFGLDKDPVLLSSLDRK<br>GVVTTGQRDWKSTLSLERVSVLEFLLQVHGSEDQDFGNYYCSVTPWVRSPTGSWQREAEIHSRP<br>IFITVKMDVLNAFKYPLLIGVGLSTVIGLLSCLIGYCSSHWCCKKEVRETRRERRRLMSMEMDG<br>GGGSVDVSKGEELFTGVVPILVELDGDVNGHKFSVSGEGEGDATYGKLTLKFICTTGKLPVPWP<br>TLVTTLTYGVQCFSRYPDHMKQHDFFKSAMPEGYVQERTIFFKDDGNYKTRAEVKFEGDTLVNR<br>IELKGIDFKEDGNILGHKLEYNYNSHNVYIMADKQKNGIKVNFKIRHNIEDGSVQLADHYQQNT<br>PIGDGPVLLPDNHYLSTQSALSKDPNEKRDHMVLLEFVTAAGITLGMDELYK***

**Supplementary Table 1. The translated sequences for all the transgenes used in this study**		
**Plasmid Number**	**Translated Fusion Protein**	**Translated Amino Acid Sequence**
Plasmid 8	PTGFRN-EGFP-Nluc (~71kDa)	MPIFNASVHSDTPSVTRGDLIKLFCIVTLEGAVLDPDDMAFDVSWFAVHSFGLDKDPVLLSSLD<br>RKGVVTTGQRDWKSTLSLERVSVLEFLLQVHGSEDQDFGNYYCSVTPWVRSPTGSWQREAEIHS<br>RPIFITVKMDVLNAFKYPLLIGVGLSTVIGLLSCLIGYCSSHWCCKKEVRETRRERRRLMSMEM<br>DGGGGSVDVSKGEELFTGVVPILVELDGDVNGHKFSVSGEGEGDATYGKLTLKFICTTGKLPVP<br>WPTLVTTLTYGVQCFSRYPDHMKQHDFFKSAMPEGYVQERTIFFKDDGNYKTRAEVKFEGDTLV<br>NRIELKGIDFKEDGNILGHKLEYNYNSHNVYIMADKQKNGIKVNFKIRHNIEDGSVQLADHYQQ<br>NTPIGDGPVLLPDNHYLSTQSALSKDPNEKRDHMVLLEFVTAAGITLGMDELYKDISGGVFTLE<br>DFVGDWRQTAGYNLDQVLEQGGVSSLFQNLGVSVTPIQRIVLSGENGLKIDIHVIIPYEGLSGD<br>QMGQIEKIFKVVYPVDDHHFKVILHYGTLVIDGVTPNMIDYFGRPYEGIAVFDGKKITVTGTLW<br>NGNKIIDERLINPDGSLLFRVTINGVTGWRLCERILA***

**Supplementary Table 2** Primary Antibodies used in this study for immunoblotting					
**Primary Antibody**	**Supplier**	**Catalog No.**	**Molecular weight (kDa)**	**Isotype**	**Concentration**
Alix	Abcam	ab186728	95	Rb	1:1000 (V/V)
CD9	CS	13174	22, 24, 25	Rb	
Flotillin-1	CS	18634	49	Rb	
TSG101	Abcam	ab125011	44	Rb	
GAPDH	CS	2118	37	Rb	
Beta-Actin	CS	4967	45	Rb	
GRP94	CS	20292	100	Rb	
EGFP	Abcam	ab184601	27	Ms	
In the above table CS: Cell Signalling, Rb: Rabbit, and Ms: Mouse					
